# Identification of four key genes related to the diagnosis of chronic obstructive pulmonary disease using bioinformatics analysis

**DOI:** 10.3389/fgene.2025.1499996

**Published:** 2025-03-05

**Authors:** Jinxia Li, Xiuming Liu, Yonghu Liu

**Affiliations:** Department of Respiratory and Critical Care Medicine, General Hospital of Ningxia Medical University, Yinchuan, China

**Keywords:** COPD, enrichment analysis, machine learning, immune infiltration analysis, drug prediction

## Abstract

**Introduction:**

Chronic obstructive pulmonary disease (COPD) is projected to become the third leading cause of death worldwide. Despite extensive research over the past few decades, effective treatments remain elusive, making disease prevention and control a global challenge.

**Methods:**

This study aimed to identify diagnostic key genes for COPD. We utilized the Gene Expression Omnibus database to obtain gene expression data specific to COPD. Differentially expressed genes (DEGs) were identified and analyzed through Gene Ontology, Kyoto Encyclopedia of Genes and Genomes, and Gene Set Enrichment Analysis. Integrated weighted gene co-expression network analysis was employed to examine related gene modules. To pinpoint key genes, we used SVM-RFE, RF, and LASSO.

**Results:**

A total of 1782 DEGs were discovered, many of which were enriched in various biological pathways and activities. Four key genes—*MRC1*, *BCL2A1*, *GYPC*, and *SLC2A3*—were identified. We observed a significant difference in immune infiltration between COPD and normal groups, indicating potential interactions between immune cells and these genes. The identified key genes were further validated using external datasets.

**Discussion:**

Our findings suggest that *MRC1*, *BCL2A1*, *GYPC*, and *SLC2A3* are potential biomarkers for COPD. Targeting these diagnostic genes with specific drugs may potentially offer new avenues for COPD management; however, this hypothesis remains preliminary and requires further investigation, as the study does not directly assess therapeutic interventions.

## 1 Introduction

Chronic obstructive pulmonary disease (COPD) is a progressive lung condition marked by airflow limitation and chronic inflammation (McDonough et al., 2011; Vestbo et al., 2013). It results from a combination of genetic factors, such as α1-antitrypsin deficiency, and environmental factors, particularly smoking (Leap et al., 2021). COPD is common and has high rates of disability and mortality, creating a significant economic burden worldwide (Iheanacho et al., 2020). Early diagnosis and treatment are crucial for slowing lung function decline and improving long-term outcomes. However, current diagnostic methods, such as pulmonary function tests and imaging, are insufficient for detecting early-stage COPD, making accurate diagnosis challenging. This highlights the need to understand genetic differences between COPD patients and healthy individuals, identify high-risk markers, and find effective treatment targets.

In recent years, high-throughput sequencing and bioinformatics have become key tools in COPD research, helping identify disease-related genes and potential molecular targets for precision therapy. For example, genes like *HIF1A*, *CDKN1A*, *BAG3*, *ERBB2,* and *ATG16L1* influence COPD through autophagy regulation (Sun et al., 2021). However, the lack of objective diagnostic methods continues to make COPD diagnosis and treatment selection difficult. Therefore, developing reliable biomarkers for COPD is essential for improving treatment outcomes.

In this study, we analyzed gene expression data from four RNA-seq datasets (GSE11906, GSE20257, GSE5058, and GSE8545) containing airway epithelial cells from COPD patients and healthy individuals. Our goal was to identify gene expression changes involved in COPD and discover potential diagnostic biomarkers. We identified 1782 differentially expressed genes (DEGs) and key COPD-related modules through analysis of two Gene Expression Omnibus (GEO) datasets. Using algorithms like SVM-RFE, random forest (RF), and LASSO, we pinpointed four key genes-*MRC1*, *BCL2A1*, *GYPC*, and *SLC2A3*-that could improve COPD diagnosis in high-risk patients. Targeting these genes with specific drugs may also enhance clinical management of COPD.

## 2 Materials and methods

### 2.1 Raw data acquisition

Datasets for four COPD airway tissues [GSE11906 (Raman et al., 2009), GSE20257 (Shaykhiev et al., 2011), GSE5058 (Carolan et al., 2006), and GSE8545 (Ammous et al., 2008)] were downloaded from the GEO database (https://www.ncbi.nlm.nih.gov/geo/). All datasets are gene expression arrays generated using the GPL570 (HG-U133_Plus_2) Affymetrix Human Genome U133 Plus 2.0 Array.

**FIGURE 1:**
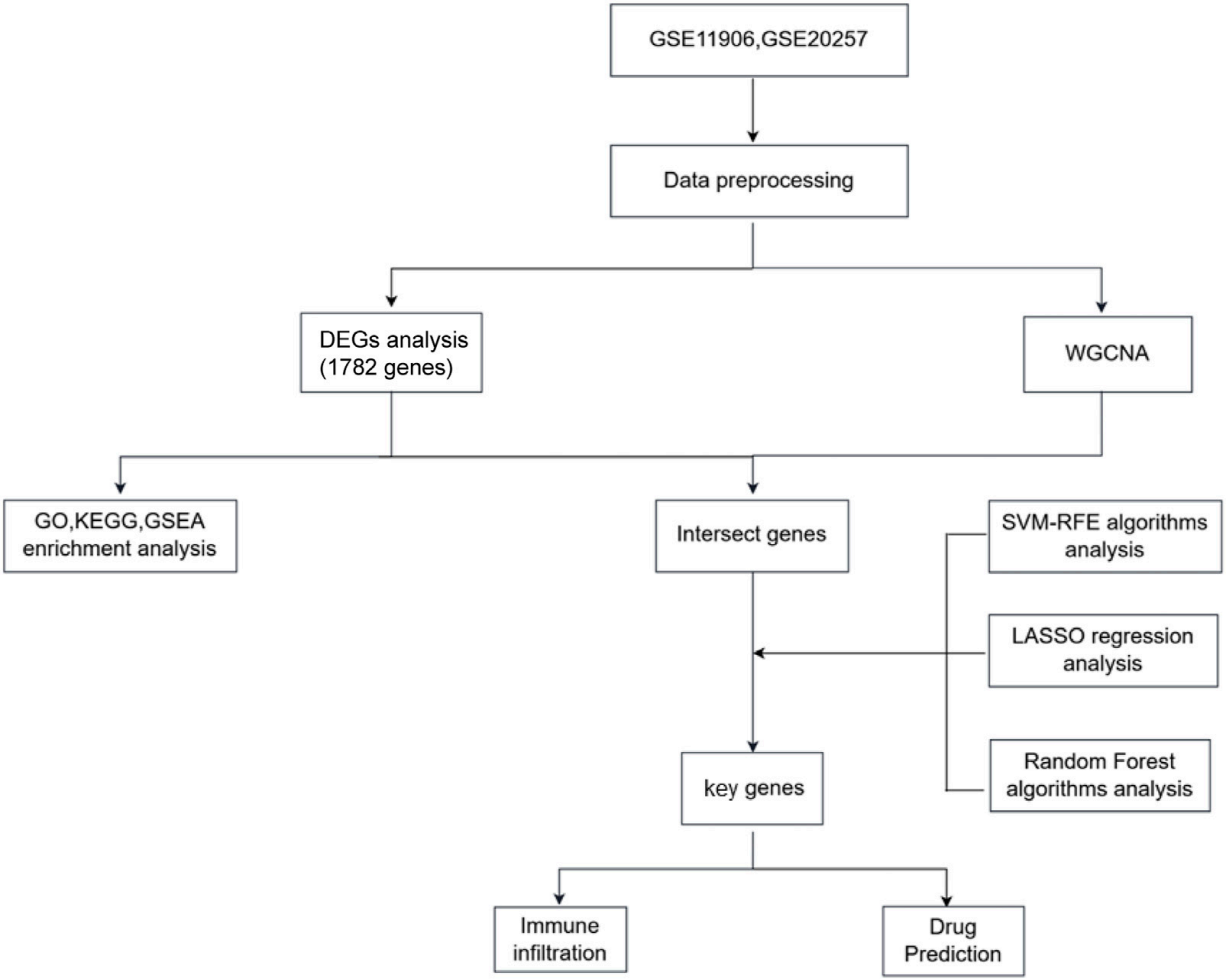
Study work flow.

GSE11906 and GSE20257 were used as the training set for airway tissue, the set contains 90 healthy and 28 COPD samples; While GSE5058 and GSE8545 were used as the validation set, the set contains 19 healthy and 21 COPD samples. The normalizeBetweenArrays function in the limma package (version 3.58.1) and sva (version 3.50.0) were applied for data combination and normalization. Probes not matching any known gene were eliminated, and if multiple probes matched a single gene, their average expression was calculated. The Perl programming language was used to remove lncRNA profiles and identify mRNA matrix files. The R package ggplot2 (version 3.2.1) was employed to normalize the processed data. Detailed information about the datasets is provided in Supplementary Table 1, and the study’s flow diagram is shown in Figure 1.

### 2.2 Differentially expressed genes identification

Principal Coordinates Analysis (PCoA), a multivariate statistical method used to assess the similarity and dissimilarity between samples, was performed based on the Bray-Curtis distance metric. PCoA was performed to confirm that the genes could effectively differentiate between healthy individuals and COPD patients. A total of 22,836 genes were tested for differential expressions, from which 1,782 were identified as significantly differentially expressed genes (DEGs) using the limma R package. The cutoff criteria for DEGs were set to an adjusted P-value <0.05 and |log fold change (FC)| > 0.5. Heatmaps and volcano plots were generated using the ggplot2 package to visualize the results.

### 2.3 Enrichment analysis

To elucidate the biological implications of the identified genes and their functions, differentially expressed genes (DEGs) were subjected to both Over-Representation Analysis (ORA) and Gene Set Enrichment Analysis (GSEA).

For ORA, enrichment analyses were performed using the Gene Ontology (GO) and Kyoto Encyclopedia of Genes and Genomes (KEGG) pathways. This analysis was conducted on the 1,782 DEGs identified after correcting the log2FC calculation error. Fisher’s Exact Test was applied for statistical analysis, and the False Discovery Rate (FDR) method was used to control the false positive rate. The analysis was performed using the clusterProfiler R package, with a significant cutoff at a P-value of less than 0.05. The terms “Molecular Function” (MF), “Biological Process” (BP), and “Cellular Component” (CC) refer to categories within the Gene Ontology classification system.

For GSEA, enrichment of predefined gene sets was determined using the reference gene set “c2. cp.kegg.v6.2. symbols.gmt” from the Molecular Signature Database (MSigDB). Enrichment sets containing fewer than 10 or more than 200 genes were excluded from the analysis. Pathways with a normalized enrichment score (NES) greater than zero were considered upregulated, while those with an NES less than zero were considered downregulated. The five most significant pathways were identified with an FDR threshold of <0.05. The weighted Kolmogorov-Smirnov statistics were employed to calculate the enrichment score (ES), with genes ranked based on log fold change (logFC) values.

### 2.4 Weighted gene co-expression network analysis

Data from GSE11906 and GSE20257 were combined and batch processed. Weighted gene co-expression network analysis (WGCNA) was used to identify trait-related modules. A topological overlap matrix was constructed from the expression profiles, with a soft-thresholding power of 18 and a minimum module size of 30 to identify core modules. A height limit of 0.25 was set for module merging. Pearson’s correlation test was then used to evaluate the modules, with a significance threshold of *P* < 0.05.

### 2.5 Support vector machine, random forest, and least absolute shrinkage and selection operator model construction

Candidate genes were identified by intersecting DEGs with genes from the WGCNA hub module. Hub genes were then classified by overlapping genes from the SVM-RFE method using the e1071 package (Noble, 2006), the RF algorithm using the randomForest R package (Paul et al., 2018), and the LASSO using the glmnet package (Vasquez et al., 2016). For Random Forest (RF), we set ntree = 1,000 and selected features with an importance score greater than 2. In LASSO, we used 10-fold cross-validation (nfolds = 10) and set the regularization parameter alpha = 1. For SVM-RFE, we applied 5-fold cross-validation (k = 5). These settings ensure the robustness and consistency of our results across different algorithms.

### 2.6 Immune infiltration analysis

To verify the association of identified genes with disease immune infiltration, the CIBERSORT algorithm was used to evaluate the proportion of 22 immune cell types in normal and COPD samples based on transcriptome data. The correlation between the identified genes and the 22 types of immune cells was subsequently analyzed.

### 2.7 Prediction of drug-gene interactions

The Drug-Gene Interaction Database (DGIdb, http://www.dgidb.org/) aggregates drug-gene interaction data from various sources, including DrugBank, PharmGKB, ChEMBL, clinical trial databases, and PubMed literature. Information on over 40,000 genes and 10,000 drugs, involving over 100,000 drug-gene interactions, was collected and organized. Key genes identified as potential pharmaceutical targets for COPD treatment were imported into DGIdb to explore existing drugs or small organic compounds. The reliability of each drug-gene interaction was evaluated based on evidence from relevant drug databases such as DrugBank. Potential therapeutic drugs for COPD were selected based on the interaction score. Results were visualized using the “ggplot2 (3.2.1)” and “ggalluvial (0.11.1)” R packages.

### 2.8 Statistical analysis

All data analyses were performed using R software (version 4.4.0). The Wilcoxon test was used for group comparisons, with *P* < 0.05 considered statistically significant.

## 3 Results

### 3.1 Differentially expressed genes identification in COPD and healthy control groups

**FIGURE 2:**
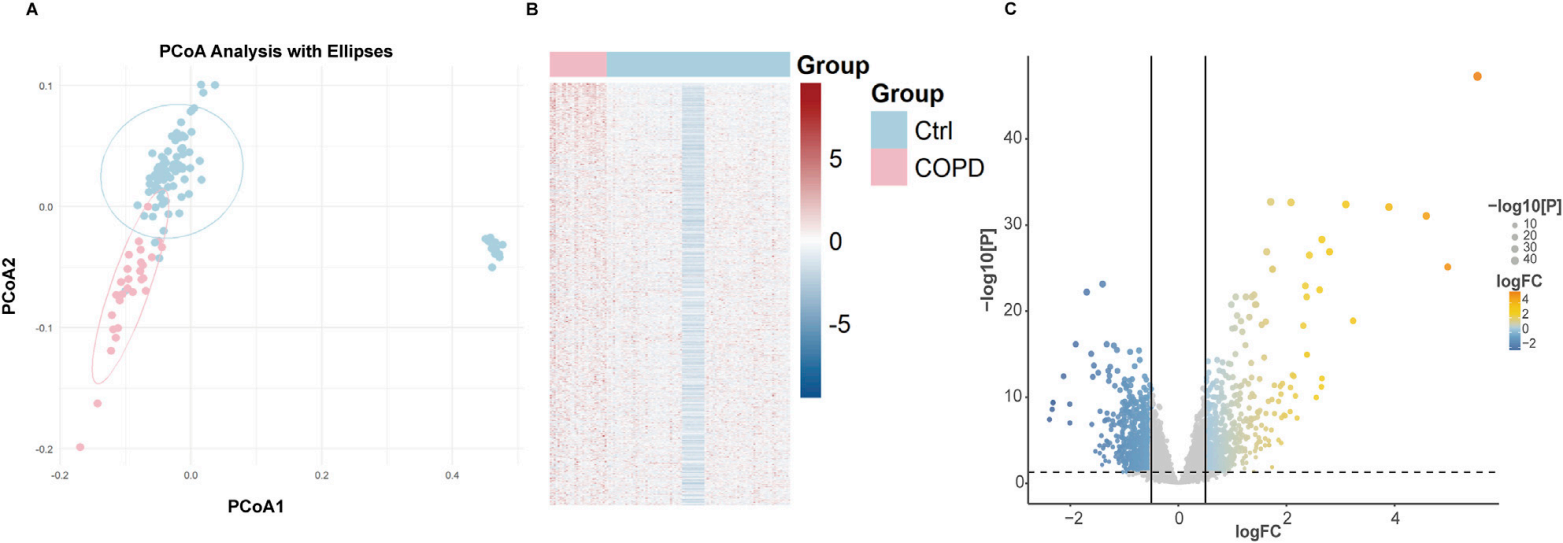
**(A)** PCoA analysis of DEGs among normal and COPD samples. **(B)** Heatmap of DEGs among normal and COPD samples. **(C)** Volcano of DEGs among normal and COPD samples.

In this study, two airway datasets (GSE11906 and GSE20257) were used to analyze differential gene expressions. The expression matrix is presented in Supplementary Table 1. To verify the stability and consistency of clustering in classifying COPD patients, Principal Coordinates Analysis (PCoA) was employed, with results displayed in Figure 2A. The integrated expression matrix revealed 1782 DEGs, of which 920 were upregulated and 862 were downregulated, as shown in Figure 2B. The volcano plot highlights DEGs with significant changes in expression levels in Figure 2C. The differentially expressed genes are detailed in Supplementary Table 2.

### 3.2 Functional analysis

**FIGURE 3:**
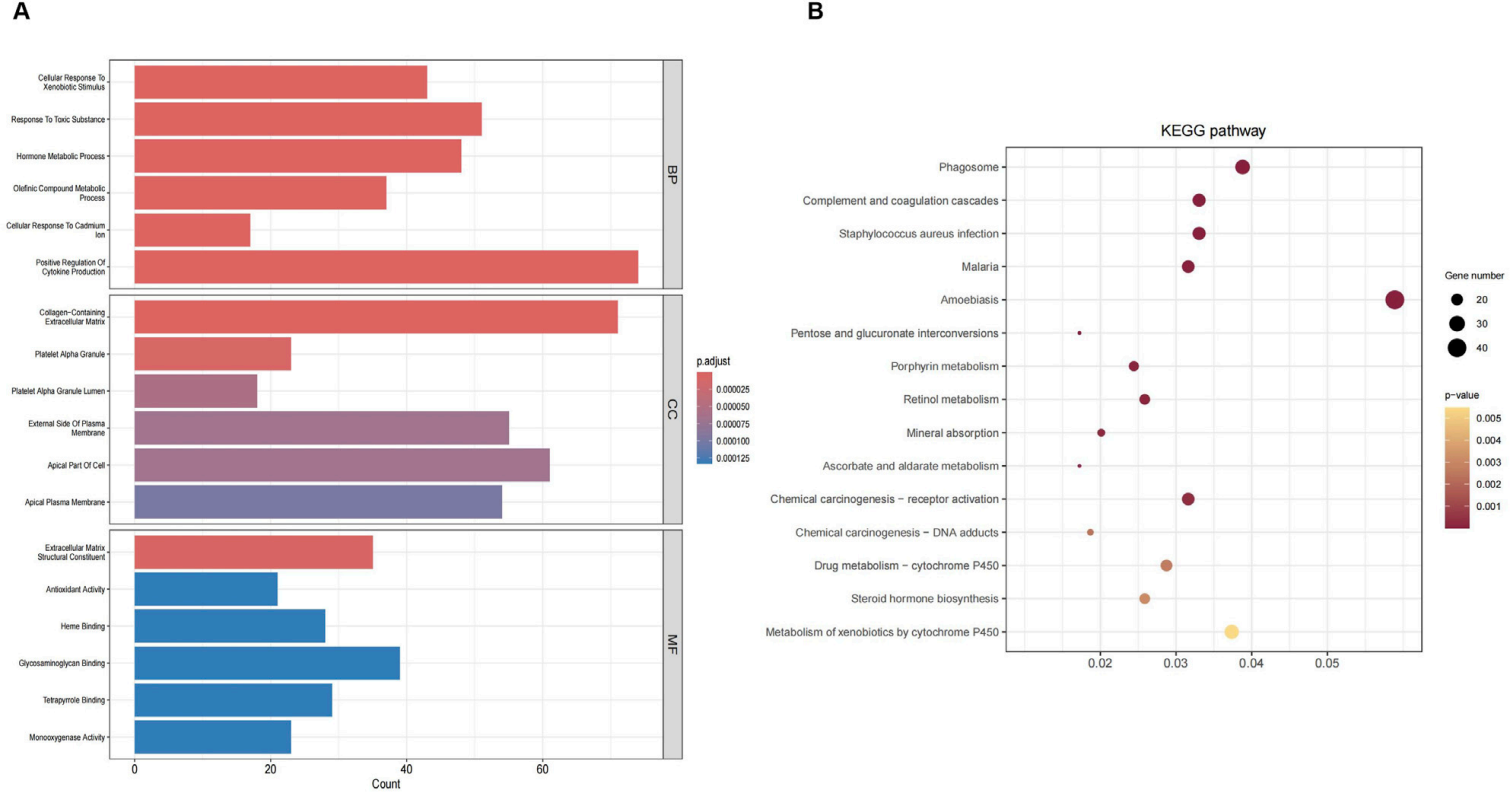
Functional DEGs enrichment. **(A)** GO analysis. **(B)** KEGG pathway analysis.

Gene Ontology (GO) analysis identified 673 biological processes (BP), 30 cellular components (CC), and 61 molecular functions (MF), as detailed in Supplementary Table 3. The top six GO items are listed in Figure 3A. The DEGs were significantly enriched in processes such as responses to xenobiotics, toxic substances, and cytokine production, as well as metabolic and hormonal regulation. They were also associated with the extracellular matrix, platelet granules, and plasma membrane components, with functions including antioxidant activity, enzyme binding, and structural roles. According to the KEGG analysis, the DEGs were enriched in various pathways, as shown in Figure 3B.

**FIGURE 4:**
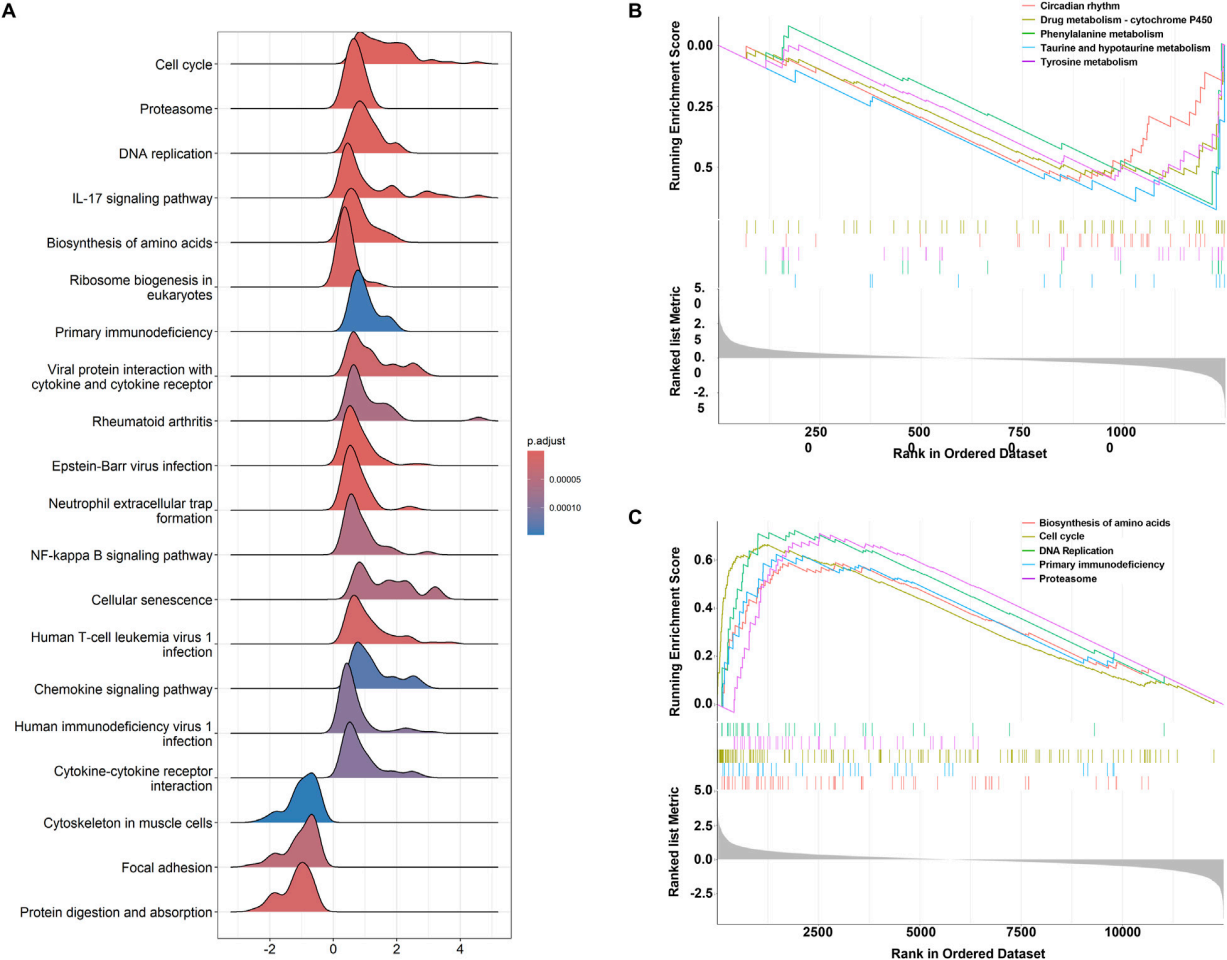
GSEA results for pathway enrichment. **(A)** Ridgeline plot of GSEA analysis results. **(B)** Top five enrichment terms for downregulated genes. **(C)** Top five enrichment terms for upregulated genes.

GSEA analysis (Supplementary Table 4) revealed distinct pathway enrichment patterns for upregulated and downregulated genes. Figure 4A shows the ridge plot of GSEA results, highlighting pathways such as the cell cycle, proteasome, DNA replication, and IL-17 signaling. Downregulated genes were enriched in circadian rhythm, drug metabolism-cytochrome P450, phenylalanine metabolism, and taurine and hypotaurine metabolism (Figure 4B). In contrast, upregulated genes were associated with amino acid biosynthesis, cell cycle, proteasome, primary immunodeficiency, and DNA replication (Figure 4C). These findings emphasize the critical roles of metabolic and immune-related pathways in the studied biological processes.

### 3.3 Overlap between COPD-Related module genes and differentially expressed genes

**FIGURE 5:**
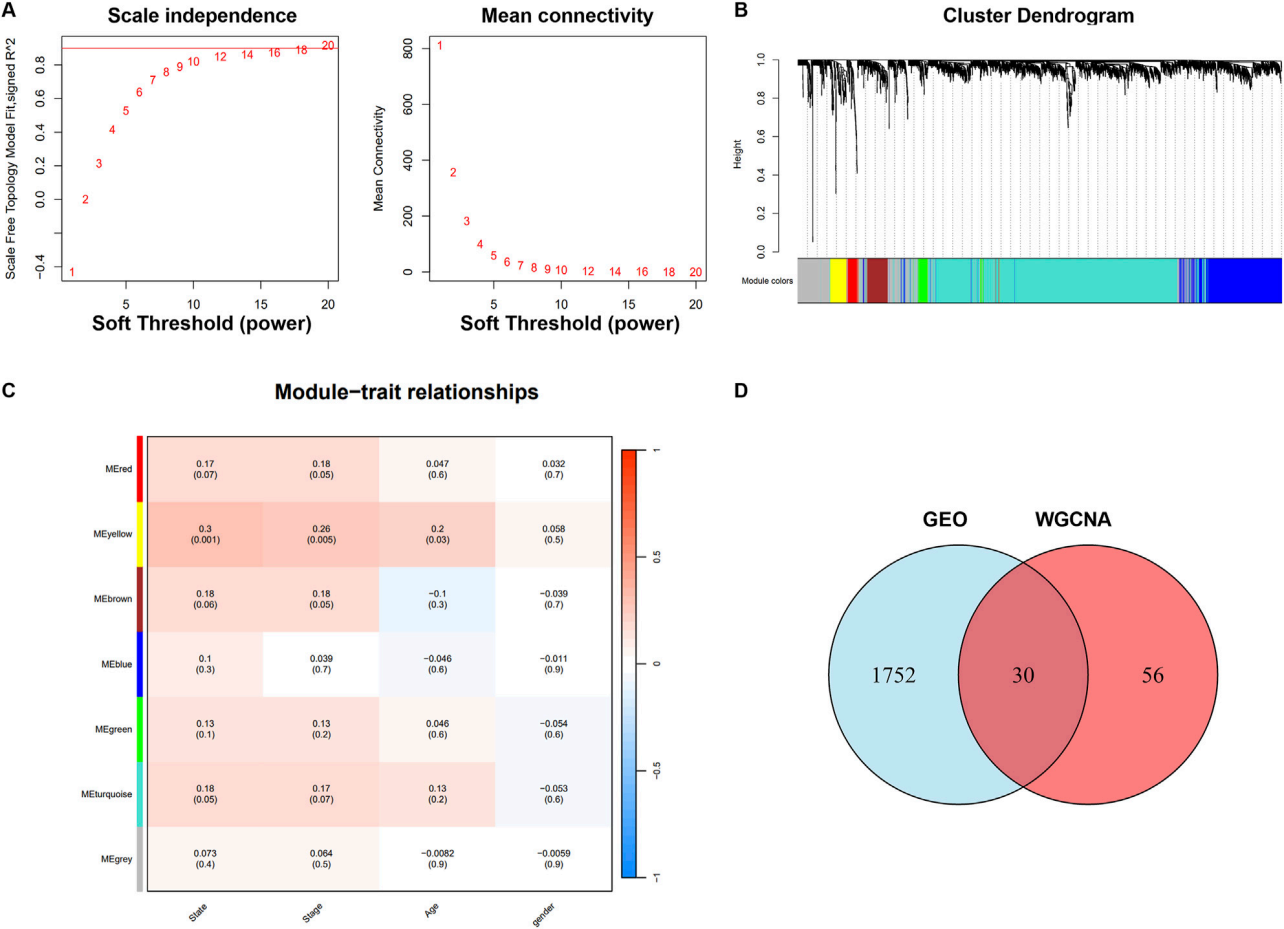
Identification of critical modules by WGCNA. **(A)** Scale-free fit index and mean connectivity for different soft-thresholding powers. **(B)** Topological overlap dissimilarity aggregation of DEGs clusters. **(C)** Module-feature correlations Each row represents a module list, whereas each column represents a clinical characteristic. The first line of each cell includes the associated correlation, while the second line gives the *P*-value. **(D)** Venn diagram for overlapped genes.

A scale-free network with a soft threshold of 18 (R^2^ = 0.9) was constructed, as shown in Figure 5A. We then computed module eigengenes, representing the overall gene expression level of each module, and grouped them based on their associations. Seven modules were identified, as depicted in Figure 5B. The yellow module was found to be correlated with COPD (cor = 0.3, P = 0.001). This module contained 86 COPD-related genes, which were retained for further investigation, as shown in Figure 5C. Ultimately, 30 genes were identified as overlapping between the DEGs and the selected module genes, as illustrated in Figure 5D.

### 3.4 Key gene identification

**FIGURE 6:**
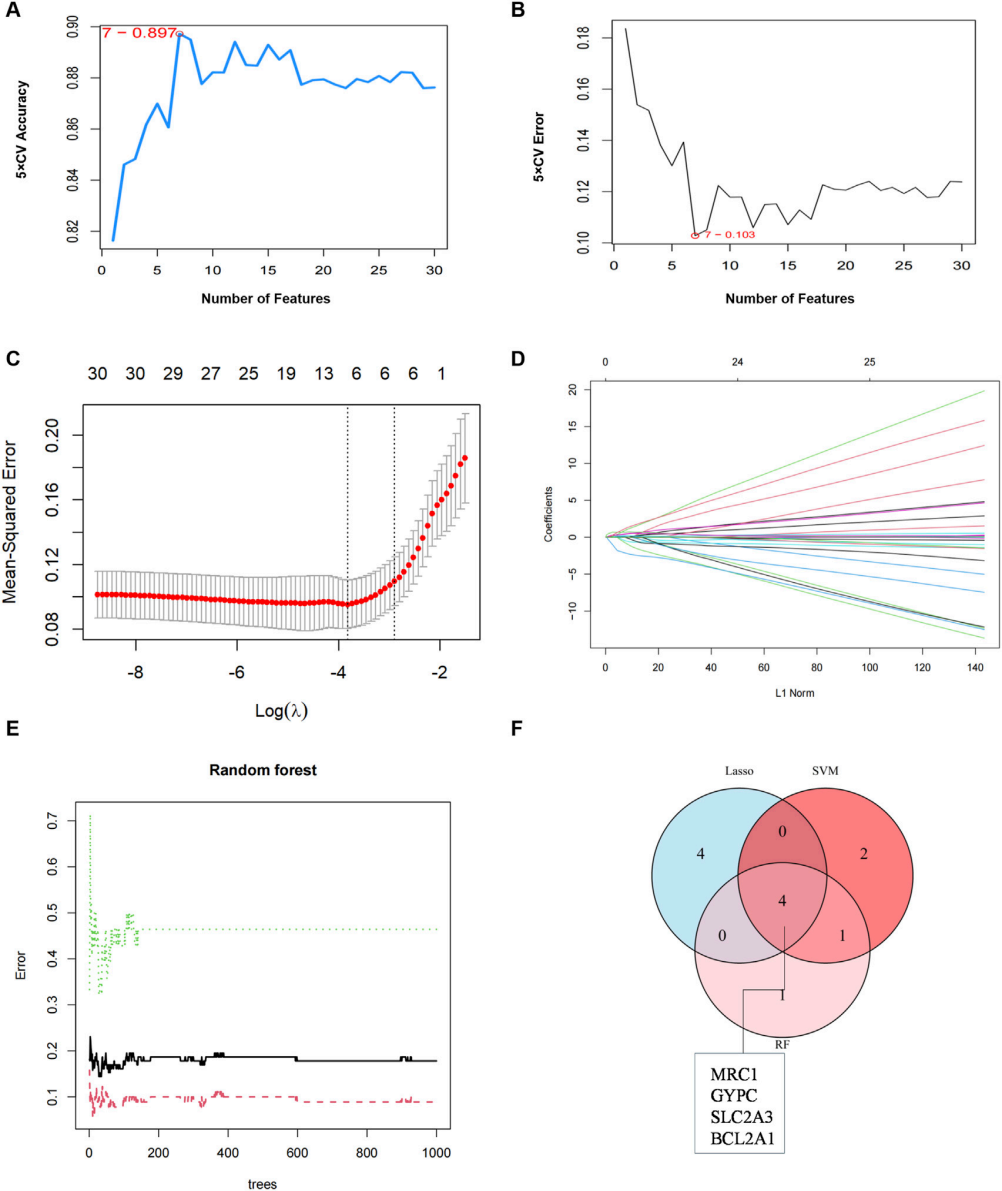
Key gene identification. **(A)** 7 gene signatures were identified by SVM-RFE analysis with an accuracy of 0.897. **(B)** Error of 0.103. **(C)** Cross-validation to select the optimal tuning parameter log(Lambda) in LASSO analysis. **(D)** LASSO coefficient profiles of candidate genes. **(E)** RF analyses identified six gene signatures **(F)** Venn diagram of four key genes shared by the SVM-RFE, RF, and LASSO algorithms.

**FIGURE 7:**
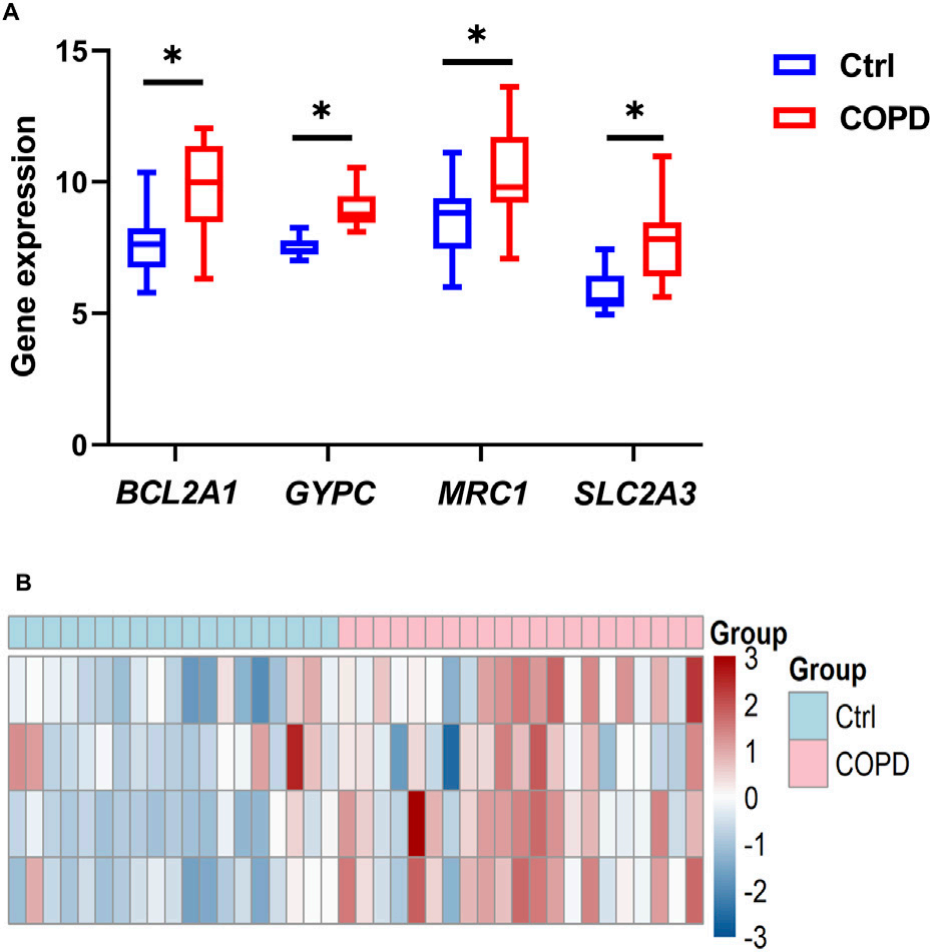
Expression analysis of key genes. **(A)** Expression of four key genes in COPD and control groups. **(B)** Heatmap of key genes expressions. *P < 0.05 vs Ctrl.

To identify gene signatures, the 30 candidate genes were analyzed using SVM-RFE, RF, and LASSO methods. Using SVM-RFE, we identified a 7-gene signature with a precision of 0.897, as shown in Figures 6A, B. LASSO analysis identified an 8-gene signature, as depicted in Figures 6C, D. RF analysis identified a 6-gene signature, as shown in Figure 6E. To establish a robust gene signature for COPD, we determined the overlapping genes from these methods, resulting in the identification of four key genes: *MRC1*, *BCL2A1*, *GYPC* and *SLC2A3*, as illustrated in Figure 6F. These four genes were significantly upgraded in COPD samples compared to controls, as shown in Figure 7A. External validation using the GSE5058 and GSE8545 datasets confirmed this trend, as shown in Figure 7B.

### 3.5 Correlation of key genes and immune cell infiltration

**FIGURE 8:**
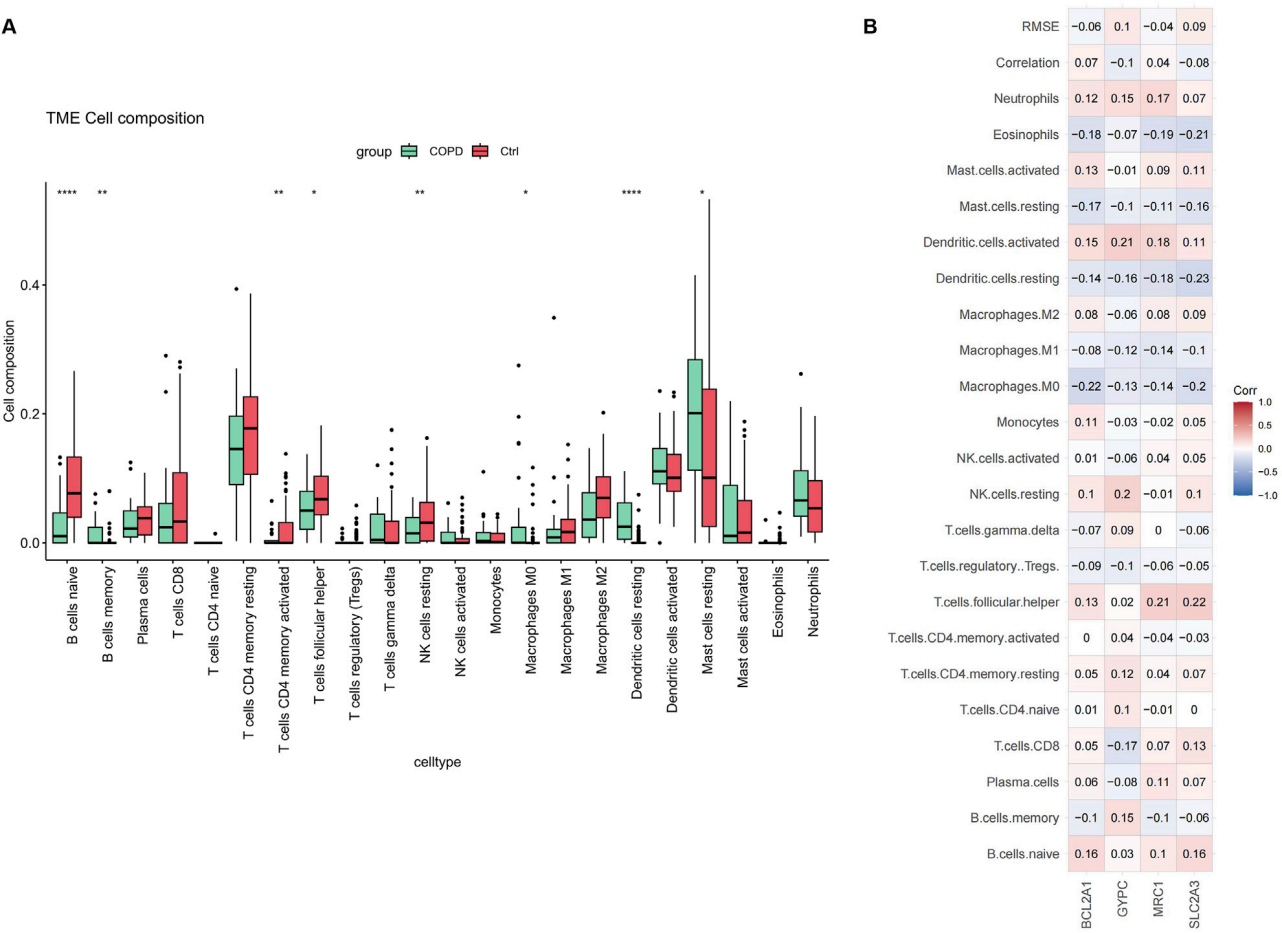
Immune cell distribution in COPD. **(A)** Differences in infiltrated immune cells between COPD and control groups. **(B)** Correlation analysis between key genes and immune cells.

Chronic inflammation of the airways, lung parenchyma, and pulmonary vasculature is a hallmark of COPD, involving inflammatory cells such as neutrophils, macrophages, and T-lymphocytes in the disease’s pathogenesis. We examined the pattern of immune cell infiltration and found that the abundance of resting mast cells, M0 macrophages, and memory B cells was significantly higher in COPD samples compared to normal samples. In contrast, native B cells, activated memory CD4 T cells, follicular helper T cells, and resting NK cells were significantly reduced, as shown in Figure 8A.

Additionally, we calculated the correlation between key gene expression and infiltrating immune cells. The results indicated that most immune cells had a positive correlation with key gene expressions, as shown in Figure 8B. These findings suggest that inflammatory components play a crucial role in the development of COPD, and that key genes may have a novel regulatory role in immune function.

### 3.6 Potential drugs targeting the diagnostic genes

**FIGURE 9:**
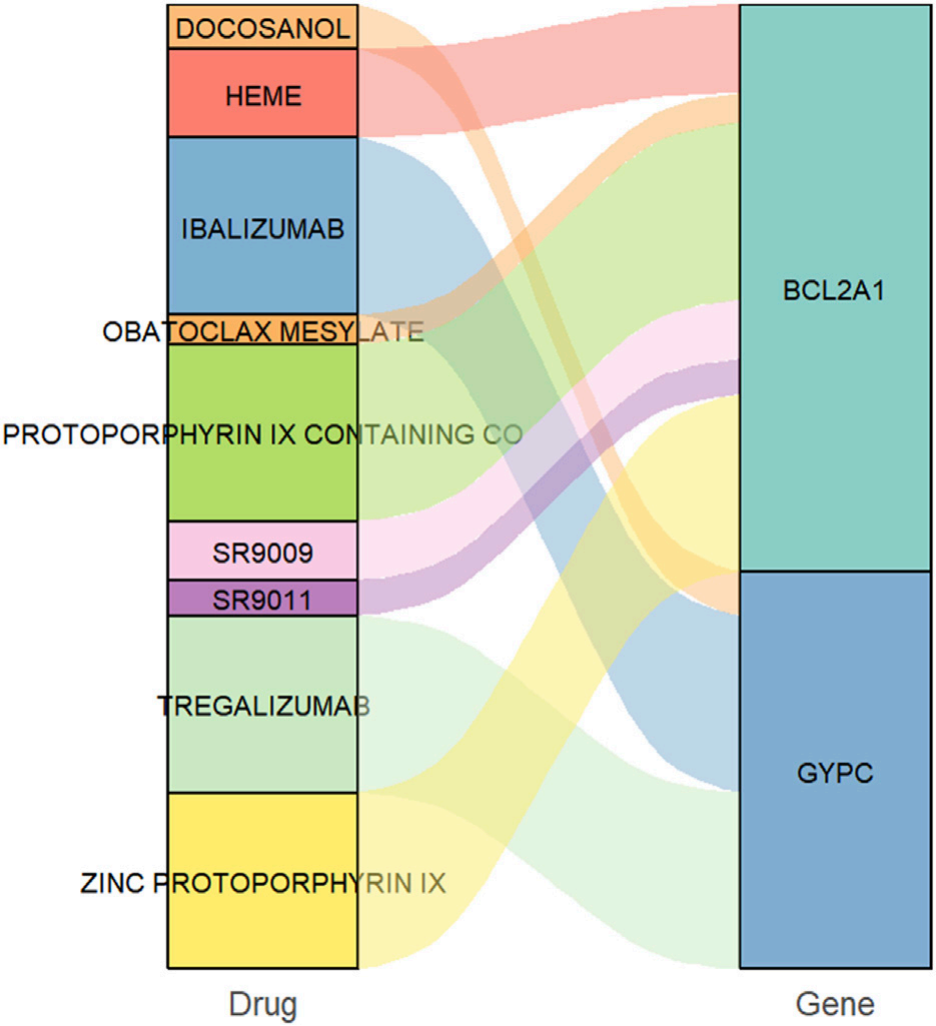
Predication of drug-gene interaction.

To identify potential drugs for COPD therapy, we searched for drugs targeting the biomarkers using the DGIdb database. As shown in Figure 9, six drugs targeting *BCL2A1* and three drugs targeting *GYPC* were identified.

## 4 Discussion

COPD is a leading cause of morbidity and mortality worldwide, with approximately 70%–80% of adults with COPD being undiagnosed (Soriano et al., 2009; Lamprecht et al., 2015; Martinez et al., 2015; Casas Herrera et al., 2016; Echazarreta et al., 2018; Gershon et al., 2018; Soriano et al., 2021). Undiagnosed patients are at increased risk of poor outcomes and a worsened quality of life, making early detection crucial for mitigating the impact of COPD and reducing the burden on healthcare systems (Larsson et al., 2019; Kostikas et al., 2020). Over the past decade, there has been growing interest in developing effective strategies and instruments for COPD detection (Lin et al., 2023). Understanding critical pathways and gene signatures in COPD could aid in risk assessment, pathogenesis elucidation, and personalized therapy development.

In this study, the top three differentially expressed genes (DEGs) identified were ME1 (Malic Enzyme 1), NQO1 (NAD(P)H Quinone Dehydrogenase 1), and CYP1B1 (Cytochrome P450 Family 1 Subfamily B Member 1), all of which have well-established roles in COPD pathogenesis. ME1 is a key enzyme involved in cellular metabolism and oxidative stress, both critical factors in the progression of COPD (Ryan et al., 2023). NQO1, an important antioxidant enzyme, plays a pivotal role in regulating oxidative stress, a hallmark feature of COPD (Li et al., 2022a). CYP1B1, on the other hand, is implicated in the metabolism of environmental toxins and xenobiotics, making it particularly relevant to COPD (Yang et al., 2020). These genes are not only highly differentially expressed but are also enriched in biological pathways central to COPD pathology, including the oxidative stress response and xenobiotic metabolism. Together, these findings highlight the potential of these genes as biomarkers or therapeutic targets in COPD research.

Advancements in bioinformatics have significantly enhanced our ability to use microarray data to uncover key genes, interaction networks, and pathways involved in COPD. In this study, both ORA and GSEA were applied to explore the biological processes influencing COPD progression. Enrichment analysis highlighted several key biological processes, including responses to xenobiotics and toxic substances, cytokine production, as well as metabolic and hormonal regulation, all of which are highly relevant to COPD pathogenesis. The response to xenobiotics and toxic substances reflects the lungs' defense mechanisms against environmental pollutants, cigarette smoke, and other harmful exposures, all of which trigger oxidative stress and inflammation-hallmarks of COPD. Previous studies have established the importance of these responses in exacerbating the disease (Christenson et al., 2022). The cytokine production pathway, crucial in amplifying the inflammatory response, also emerged as a significant factor in COPD. This process contributes to tissue damage and airway remodeling, which are central features of the disease (Barnes, 2009). Furthermore, metabolic and hormonal regulation emphasizes the systemic nature of COPD, suggesting that metabolic dysregulation and hormonal imbalances may exacerbate disease progression. Recent research supports targeted reprogramming of metabolism as a promising therapeutic approach for respiratory diseases like COPD (Gan et al., 2024). Together, these findings corroborate previous studies and underscore the importance of these biological processes as potential diagnostic, prognostic, and therapeutic targets in COPD.

In our study, GSEA provided a deeper insight into the specific biological pathways enriched among DEGs. Notably, GSEA revealed that genes were primarily enriched in the *IL-17* signaling pathway, circadian rhythm, and drug metabolism-cytochrome P450. *IL-17* plays a crucial role in lung lymphoid neogenesis in COPD, contributing to airway inflammation, remodeling, and mucus hypersecretion (Kramer and Gaffen, 2007; Xiong et al., 2020; Henen et al., 2023). Preclinical studies have shown that anti-*IL-17* antibodies can reduce airway inflammation and remodeling in COPD models, supporting *IL-17* as a potential therapeutic target (Yousuf et al., 2019). Additionally, the circadian rhythm pathway emerged as significant in COPD pathogenesis. Disruption of circadian rhythms has been linked to various lung diseases, and the circadian clock gene Clock-Bmal1 has been shown to regulate cellular responses to inflammation and immune activation in the lungs. This pathway may hold therapeutic potential for improving COPD outcomes by restoring circadian regulation (Li et al., 2022b). Although both ORA and GSEA identified pathways related to inflammation and immune response, their approaches provided complementary perspectives. ORA helped pinpoint over-represented functional categories among the most significantly differentially expressed genes, while GSEA offered a broader view by analyzing the entire ranked gene list. This allowed GSEA to identify pathways enriched at both ends of the gene expression spectrum, capturing subtle shifts in pathway activation that ORA might have missed. For example, GSEA highlighted pathways like the *IL-17* signaling pathway and circadian rhythm, which, while not dominated by a small number of highly differentially expressed genes, represent important, biologically significant alterations in COPD. These insights underscore the value of using both enrichment methods in combination to gain a more comprehensive understanding of the molecular mechanisms driving COPD.

Recent research has confirmed that innate and adaptive immune mechanisms play essential roles in COPD progression (Caramori et al., 2016; Bu et al., 2020). In this study, resting mast cells, M0 macrophages, and memory B cells were found to be upregulated in COPD samples. Macrophages and B cells are critical immune cells in COPD pathogenesis (Seys et al., 2015; Lee et al., 2016; Kapellos et al., 2018; Sullivan et al., 2019), and mast cells may also play an important role. Increased reticular basement membrane and lamina propria mast cells, as well as perivascular mast cells involved in angiogenesis, have been observed in COPD patients (Soltani et al., 2012). Understanding biology, heterogeneity, activation mechanisms, and signaling cascades of immune cells could lead to novel therapies for COPD.

In our study, four key genes were identified as being related to COPD. Mannose receptor C-type 1 (*MRC1*) is a critical regulator in macrophage-mediated immune responses (van der Zande et al., 2021). This receptor plays a significant role in several biological processes, including the regulation of circulating reproductive hormones, homeostasis, innate immunity, and infection responses (Cummings, 2022). Recent studies have highlighted the role of *MRC1* in macrophage activation (Gantzel et al., 2020), a process crucial for chronic inflammation and tissue remodeling in COPD. Our findings suggest that *MRC1* may serve as a potential biomarker for COPD progression, particularly in immune regulation and the inflammatory pathways associated with the disease.

B-cell lymphoma 2-related protein A1 (*BCL2A1*), a highly regulated *NF-κB* target gene, is known for its pro-survival roles in the hematopoietic system and is overexpressed in various cancers, contributing to tumor progression (Vogler, 2012; Yue et al., 2021; Gao et al., 2023). *BCL2A1* has also been implicated in protecting against acute lung injury (Ren et al., 2024), although its direct role in COPD remains underexplored. Our study reveals that *BCL2A1* is highly expressed in the airway epithelial cells of COPD patients, suggesting that it may play an important role in the pathogenesis of COPD and could serve as a potential therapeutic target.

Glycophorin C (*GYPC*) is a membrane protein primarily expressed in red blood cells, where it is involved in cell adhesion and maintaining structural integrity. Although its role in pulmonary diseases is not well understood, previous studies have proposed the red blood cell as a biosensor for monitoring oxidative stress and imbalance in COPD (Lucantoni et al., 2006). N our study, *GYPC* expression was significantly upregulated in COPD patients, indicating its potential involvement in the altered immune landscape in COPD and its promise as a biomarker for disease progression.

Solute carrier family 2 member 3 (*SLC2A3*), also known as *GLUT3*, is a high-affinity glucose transporter involved in cellular energy metabolism. Overexpression of *SLC2A3* has been shown to promote cell survival and growth in cancer (Yao et al., 2020; Yan et al., 2023). Our analysis, which focused on the immune microenvironment of COPD patients, revealed that *SLC2A3* was expressed in macrophages from COPD patients and was upregulated in THP-M cells and lung tissues of these patients (Zhang et al., 2023). In COPD, *SLC2A3* appears to play a crucial role in maintaining energy homeostasis under conditions of chronic inflammation and hypoxic stress. These findings suggest that SLC2A3 could be a promising biomarker for COPD diagnosis and therapy, particularly in the context of metabolic reprogramming during disease progression.

To uncover diagnostic indications for COPD, we applied SVM-RFE, LASSO, and RF algorithms, and used CIBERSORT to examine immune cell infiltration. This study identified *MRC1*, *BCL2A1*, *GYPC* and *SLC2A3* as COPD diagnostic indicators. However, studying has several limitations. Firstly, the key genes should be validated by qPCR, and their localization and distribution should be verified. Secondly, the study scope did not include detailed *in vivo* and *in vitro* validation. Finally, our findings were derived from bioinformatics analysis, and the specific mechanisms by which key genes affect COPD prognosis need further experimental confirmation.

One limitation of this study is the relatively small sample size, with the validation set comprising only 21 COPD patients and 19 controls. This may limit the statistical power and generalizability of the findings. However, despite the small sample size, we ensured the robustness of our results by validating the identified hub genes and pathways across multiple independent datasets. These datasets consistently supported our findings, which enhances the reliability of our conclusions and suggests that the observed gene expression patterns may be applicable to other cohorts.

Another limitation is the use of older datasets, with one microarray dataset being nearly 20 years old. Although these datasets are still widely cited, advances in sequencing technologies and metadata standards may impact their generalizability. Therefore, future studies should incorporate updated datasets and experimental validation to further confirm our findings.

To address the sample size limitation, we emphasize the need for future studies to utilize larger validation cohorts. A larger sample size would not only improve statistical power but also increase the generalizability of our findings across different patient populations. We believe these efforts will provide a more solid foundation for confirming the clinical relevance of the identified genes and pathways.

## Data Availability

The original contributions presented in the study are included in the article/Supplementary Material, further inquiries can be directed to the corresponding author.
