# Supplementary material for: Identification of four key genes related to the diagnosis of chronic obstructive pulmonary disease using bioinformatics analysis
Source: Front Genet. 2025 Mar 5;16:1499996. doi: 10.3389/fgene.2025.1499996 (PMC11919834; doi:10.3389/fgene.2025.1499996)
Supplement: Supplementary file 1 [file DataSheet2.pdf]

Supplementary table 3. GO enrichment

ONTOLOGY	ID	pvalue	p.adjust	qvalue	Count
BP	GO:0071466	3.01E-12	1.15E-08	8.88E-09	43
BP	GO:0009636	4.19E-12	1.15E-08	8.88E-09	51
BP	GO:0042445	8.69E-12	1.59E-08	1.23E-08	48
BP	GO:0120254	3.89E-11	5.35E-08	4.13E-08	37
BP	GO:0071276	5.11E-11	5.62E-08	4.34E-08	17
BP	GO:0001819	1.39E-10	1.27E-07	9.81E-08	74
BP	GO:0061687	1.64E-10	1.29E-07	9.95E-08	13
BP	GO:0042330	2.76E-10	1.89E-07	1.46E-07	70
BP	GO:0098754	3.20E-10	1.95E-07	1.50E-07	35
BP	GO:0009410	4.58E-10	2.52E-07	1.94E-07	67
BP	GO:0006935	5.78E-10	2.89E-07	2.23E-07	69
BP	GO:0097501	1.45E-09	6.65E-07	5.13E-07	12
BP	GO:0033559	1.96E-09	8.30E-07	6.40E-07	28
BP	GO:0002526	2.53E-09	9.95E-07	7.67E-07	27
BP	GO:0006690	3.38E-09	1.24E-06	9.55E-07	29
BP	GO:0006805	4.11E-09	1.41E-06	1.09E-06	29
BP	GO:0071248	5.72E-09	1.85E-06	1.43E-06	38
BP	GO:0046686	7.41E-09	2.26E-06	1.75E-06	19
BP	GO:0002237	1.01E-08	2.93E-06	2.26E-06	56
BP	GO:0002274	1.32E-08	3.64E-06	2.81E-06	42
BP	GO:0019748	1.93E-08	5.05E-06	3.90E-06	18
BP	GO:0032496	2.22E-08	5.55E-06	4.28E-06	53
BP	GO:0072593	2.76E-08	6.37E-06	4.91E-06	41
BP	GO:0010038	2.78E-08	6.37E-06	4.91E-06	54
BP	GO:0042060	2.95E-08	6.48E-06	5.00E-06	61
BP	GO:0071280	3.47E-08	7.33E-06	5.66E-06	12
BP	GO:0050900	5.03E-08	1.02E-05	7.90E-06	57
BP	GO:1990748	7.98E-08	1.55E-05	1.20E-05	26
BP	GO:0003018	8.20E-08	1.55E-05	1.20E-05	44
BP	GO:0097237	1.16E-07	2.12E-05	1.64E-05	27
BP	GO:0001822	1.48E-07	2.63E-05	2.03E-05	48
BP	GO:0002768	1.73E-07	2.97E-05	2.29E-05	52
BP	GO:0010273	2.14E-07	3.46E-05	2.67E-05	9
BP	GO:1990169	2.14E-07	3.46E-05	2.67E-05	9
BP	GO:0071241	2.39E-07	3.76E-05	2.90E-05	38
BP	GO:0097529	2.62E-07	3.99E-05	3.08E-05	39
BP	GO:0006959	2.71E-07	4.03E-05	3.11E-05	41
BP	GO:0006882	3.12E-07	4.50E-05	3.47E-05	13
BP	GO:0018108	3.19E-07	4.50E-05	3.47E-05	42
BP	GO:0052695	3.44E-07	4.72E-05	3.64E-05	10
BP	GO:0016101	3.57E-07	4.78E-05	3.69E-05	21
BP	GO:0072001	3.79E-07	4.96E-05	3.82E-05	48
BP	GO:0018212	3.92E-07	5.01E-05	3.86E-05	42
BP	GO:0006766	4.35E-07	5.43E-05	4.19E-05	24
BP	GO:0045785	4.50E-07	5.49E-05	4.23E-05	63
BP	GO:0060326	4.93E-07	5.69E-05	4.39E-05	47
BP	GO:1901654	4.96E-07	5.69E-05	4.39E-05	36
BP	GO:0002429	4.97E-07	5.69E-05	4.39E-05	48
BP	GO:0098869	5.75E-07	6.34E-05	4.89E-05	22
BP	GO:0001523	5.77E-07	6.34E-05	4.89E-05	20

BP	GO:0010466	6.89E-07	7.29E-05	5.63E-05	26
BP	GO:0006721	6.90E-07	7.29E-05	5.63E-05	22
BP	GO:0002685	7.54E-07	7.82E-05	6.03E-05	37
BP	GO:0042573	8.18E-07	8.32E-05	6.42E-05	12
BP	GO:0019369	9.33E-07	9.32E-05	7.19E-05	16
BP	GO:0071216	1.46E-06	0.000143659	0.000110794	40
BP	GO:0030595	1.59E-06	0.000152891	0.000117914	37
BP	GO:0045807	1.62E-06	0.000153745	0.000118573	28
BP	GO:0042743	2.38E-06	0.000215177	0.000165951	15
BP	GO:0071706	2.39E-06	0.000215177	0.000165951	32
BP	GO:1903555	2.39E-06	0.000215177	0.000165951	32
BP	GO:0010951	2.53E-06	0.000224291	0.000172979	24
BP	GO:0071674	3.23E-06	0.00028187	0.000217386	33
BP	GO:0050853	3.39E-06	0.000291371	0.000224714	18
BP	GO:0006063	3.79E-06	0.000311345	0.000240118	10
BP	GO:0019585	3.79E-06	0.000311345	0.000240118	10
BP	GO:0032640	3.85E-06	0.000311345	0.000240118	31
BP	GO:0032680	3.85E-06	0.000311345	0.000240118	31
BP	GO:0051897	3.98E-06	0.000317019	0.000244493	29
BP	GO:0006909	4.17E-06	0.000327327	0.000252443	36
BP	GO:0045926	4.30E-06	0.000330237	0.000254688	37
BP	GO:0010232	4.39E-06	0.000330237	0.000254688	19
BP	GO:0150104	4.39E-06	0.000330237	0.000254688	19
BP	GO:0046688	4.55E-06	0.000336996	0.000259901	12
BP	GO:0071219	4.60E-06	0.000336996	0.000259901	36
BP	GO:0031589	4.71E-06	0.000340231	0.000262396	48
BP	GO:0050920	4.90E-06	0.000349976	0.000269911	35
BP	GO:0007584	5.08E-06	0.00035808	0.000276161	28
BP	GO:0001503	5.54E-06	0.000385256	0.00029712	56
BP	GO:0031960	5.73E-06	0.00039039	0.000301079	27
BP	GO:0031667	5.75E-06	0.00039039	0.000301079	60
BP	GO:0070371	6.47E-06	0.000433362	0.00033422	45
BP	GO:0002697	6.86E-06	0.000454229	0.000350314	50
BP	GO:0009812	7.63E-06	0.000499064	0.000384892	7
BP	GO:1903131	7.89E-06	0.000510262	0.000393528	59
BP	GO:0009595	8.26E-06	0.000528072	0.000407263	12
BP	GO:0022407	8.42E-06	0.000531616	0.000409996	60
BP	GO:0043410	8.94E-06	0.000558132	0.000430447	58
BP	GO:0006775	9.43E-06	0.000575534	0.000443867	13
BP	GO:0006953	9.43E-06	0.000575534	0.000443867	13
BP	GO:0042180	1.04E-05	0.000627638	0.000484052	34
BP	GO:0032102	1.07E-05	0.00064135	0.000494627	56
BP	GO:0051960	1.15E-05	0.000679267	0.00052387	57
BP	GO:0071294	1.49E-05	0.000871539	0.000672155	9
BP	GO:0050678	1.52E-05	0.000876051	0.000675635	51
BP	GO:0071695	1.53E-05	0.000876051	0.000675635	38
BP	GO:0019852	1.57E-05	0.000881349	0.00067972	6
BP	GO:1901334	1.57E-05	0.000881349	0.00067972	6
BP	GO:0050766	1.95E-05	0.001080111	0.000833011	16
BP	GO:0006693	1.97E-05	0.001080111	0.000833011	13
BP	GO:1903557	2.02E-05	0.001090522	0.00084104	21
BP	GO:0042440	2.02E-05	0.001090522	0.00084104	17

BP	GO:0070374	2.13E-05	0.001136029	0.000876137	32
BP	GO:0071222	2.17E-05	0.0011452	0.000883209	33
BP	GO:0050878	2.28E-05	0.001195096	0.000921691	47
BP	GO:0009612	2.34E-05	0.001204351	0.000928828	32
BP	GO:0006801	2.35E-05	0.001204351	0.000928828	16
BP	GO:0071675	2.42E-05	0.001228963	0.00094781	22
BP	GO:0006692	2.48E-05	0.001248222	0.000962663	13
BP	GO:0070372	2.52E-05	0.00125446	0.000967474	41
BP	GO:0008202	2.53E-05	0.00125446	0.000967474	43
BP	GO:0010721	2.67E-05	0.001310502	0.001010695	39
BP	GO:0050673	2.72E-05	0.001322052	0.001019603	57
BP	GO:0050921	2.79E-05	0.001344391	0.001036831	24
BP	GO:0007162	2.94E-05	0.001403923	0.001082744	41
BP	GO:0031214	3.04E-05	0.001440175	0.001110703	28
BP	GO:0072073	3.08E-05	0.001442946	0.00111284	25
BP	GO:0010517	3.10E-05	0.001442946	0.00111284	13
BP	GO:0002065	3.13E-05	0.001445113	0.001114511	22
BP	GO:0051384	3.16E-05	0.001446693	0.001115729	23
BP	GO:0052548	3.21E-05	0.001457385	0.001123976	38
BP	GO:0150076	3.35E-05	0.001509606	0.00116425	17
BP	GO:0030100	3.38E-05	0.001510354	0.001164826	39
BP	GO:0050764	3.59E-05	0.00158899	0.001225473	19
BP	GO:0071214	3.65E-05	0.001589704	0.001226024	43
BP	GO:0104004	3.65E-05	0.001589704	0.001226024	43
BP	GO:0032760	3.82E-05	0.001652116	0.001274157	20
BP	GO:0052547	4.11E-05	0.001763664	0.001360186	40
BP	GO:0050731	4.14E-05	0.001763664	0.001360186	26
BP	GO:0032354	4.24E-05	0.001790168	0.001380627	7
BP	GO:0006631	4.27E-05	0.001790168	0.001380627	49
BP	GO:0051896	4.35E-05	0.001809898	0.001395843	35
BP	GO:1902105	5.07E-05	0.002094505	0.00161534	42
BP	GO:0048469	5.25E-05	0.002126294	0.001639857	30
BP	GO:0034113	5.29E-05	0.002126294	0.001639857	14
BP	GO:0050866	5.30E-05	0.002126294	0.001639857	31
BP	GO:0050730	5.30E-05	0.002126294	0.001639857	32
BP	GO:0048732	5.41E-05	0.002152508	0.001660073	53
BP	GO:0002444	5.71E-05	0.002256569	0.001740328	20
BP	GO:0090596	6.17E-05	0.002409102	0.001857966	37
BP	GO:0043491	6.19E-05	0.002409102	0.001857966	39
BP	GO:0045730	6.23E-05	0.002409102	0.001857966	11
BP	GO:0007159	6.60E-05	0.002537865	0.001957271	50
BP	GO:0008544	7.06E-05	0.002692869	0.002076814	47
BP	GO:0002757	7.60E-05	0.002878859	0.002220255	57
BP	GO:0006911	7.74E-05	0.002912096	0.002245889	12
BP	GO:0002275	7.88E-05	0.002944026	0.002270513	18
BP	GO:0071774	8.39E-05	0.003102788	0.002392955	21
BP	GO:0002689	8.41E-05	0.003102788	0.002392955	8
BP	GO:0007160	8.69E-05	0.003147215	0.002427219	33
BP	GO:0061900	8.71E-05	0.003147215	0.002427219	13
BP	GO:0170040	8.71E-05	0.003147215	0.002427219	13
BP	GO:0045861	8.84E-05	0.003176663	0.002449929	32
BP	GO:0010810	8.95E-05	0.003179005	0.002451736	31

BP	GO:0002443	8.97E-05	0.003179005	0.002451736	54
BP	GO:0043524	9.05E-05	0.00318826	0.002458874	25
BP	GO:0032732	9.22E-05	0.003204864	0.002471679	15
BP	GO:0071260	9.22E-05	0.003204864	0.002471679	15
BP	GO:0050727	9.46E-05	0.00324174	0.002500119	50
BP	GO:0044344	9.50E-05	0.00324174	0.002500119	20
BP	GO:0002263	9.55E-05	0.00324174	0.002500119	39
BP	GO:0050729	9.56E-05	0.00324174	0.002500119	24
BP	GO:0016486	9.72E-05	0.003277688	0.002527843	10
BP	GO:0006720	0.000103766	0.003476797	0.002681401	22
BP	GO:0002830	0.000104701	0.003486873	0.002689172	7
BP	GO:0097530	0.00010609	0.003511838	0.002708426	24
BP	GO:0042446	0.000109198	0.003571695	0.002754589	14
BP	GO:0042531	0.000109198	0.003571695	0.002754589	14
BP	GO:0030099	0.000111284	0.003618363	0.002790581	51
BP	GO:0043588	0.00011208	0.003622806	0.002794007	40
BP	GO:0006636	0.000117397	0.003752437	0.002893982	12
BP	GO:0051346	0.000117456	0.003752437	0.002893982	29
BP	GO:1903035	0.000118578	0.003766405	0.002904755	18
BP	GO:0001558	0.000120857	0.003804207	0.002933909	49
BP	GO:0001676	0.000121153	0.003804207	0.002933909	19
BP	GO:0021700	0.000122813	0.003834417	0.002957207	42
BP	GO:0008207	0.00012522	0.00388749	0.002998139	10
BP	GO:0043616	0.00012683	0.003915337	0.003019615	13
BP	GO:0034329	0.00012977	0.003983712	0.003072348	52
BP	GO:0071621	0.000133919	0.004088261	0.003152979	21
BP	GO:0007596	0.000135921	0.004100793	0.003162644	31
BP	GO:1903707	0.000136671	0.004100793	0.003162644	20
BP	GO:0010720	0.000137183	0.004100793	0.003162644	52
BP	GO:0042116	0.000137315	0.004100793	0.003162644	19
BP	GO:0030198	0.000138834	0.004122393	0.003179303	41
BP	GO:0001763	0.000139718	0.004122393	0.003179303	29
BP	GO:0045664	0.000140289	0.004122393	0.003179303	28
BP	GO:0042572	0.00014332	0.004189052	0.003230712	12
BP	GO:0043062	0.000148238	0.004309894	0.003323908	41
BP	GO:0046456	0.000152002	0.004357712	0.003360787	13
BP	GO:1905517	0.000152002	0.004357712	0.003360787	13
BP	GO:0002366	0.00015245	0.004357712	0.003360787	38
BP	GO:0045766	0.000153055	0.004357712	0.003360787	27
BP	GO:0045229	0.000158214	0.004481379	0.003456162	41
BP	GO:0002687	0.000169425	0.004774297	0.003682069	23
BP	GO:1903706	0.000170704	0.004785809	0.003690947	49
BP	GO:1902106	0.000175418	0.004893016	0.003773628	19
BP	GO:0098883	0.000177485	0.004925665	0.003798808	6
BP	GO:0022409	0.00017894	0.004928936	0.00380133	40
BP	GO:0007204	0.000179989	0.004928936	0.00380133	25
BP	GO:0060191	0.000180294	0.004928936	0.00380133	14
BP	GO:0170035	0.000181353	0.004933329	0.003804719	13
BP	GO:1903034	0.000182345	0.004935884	0.003806689	26
BP	GO:0051962	0.000197136	0.005310121	0.004095311	37
BP	GO:0030308	0.000199884	0.005331852	0.004112071	27
BP	GO:1904018	0.000199884	0.005331852	0.004112071	27

BP	GO:0050817	0.000202801	0.005383522	0.00415192	31
BP	GO:0048545	0.000204459	0.005401444	0.004165742	41
BP	GO:0002548	0.000211529	0.005561491	0.004289175	14
BP	GO:0032731	0.000215436	0.005625232	0.004338333	13
BP	GO:0002688	0.000216826	0.005625232	0.004338333	20
BP	GO:0030856	0.000217024	0.005625232	0.004338333	25
BP	GO:0009065	0.000223358	0.00570861	0.004402637	8
BP	GO:0042537	0.000223358	0.00570861	0.004402637	8
BP	GO:0098581	0.000223358	0.00570861	0.004402637	8
BP	GO:0002577	0.000226209	0.005754714	0.004438193	7
BP	GO:0048608	0.000228672	0.005771217	0.004450921	38
BP	GO:0060560	0.000228958	0.005771217	0.004450921	32
BP	GO:0042476	0.000232262	0.005824285	0.004491848	21
BP	GO:0043299	0.000233183	0.005824285	0.004491848	15
BP	GO:0140353	0.000235907	0.005859492	0.004519001	11
BP	GO:0007599	0.000236896	0.005859492	0.004519001	31
BP	GO:0002695	0.000237792	0.005859492	0.004519001	27
BP	GO:0033627	0.000246766	0.006053483	0.004668612	16
BP	GO:0030857	0.000252374	0.006120006	0.004719916	12
BP	GO:0001953	0.000253025	0.006120006	0.004719916	10
BP	GO:0140448	0.000253025	0.006120006	0.004719916	10
BP	GO:0034308	0.000253933	0.006120006	0.004719916	18
BP	GO:0030638	0.00026597	0.006272566	0.004837575	5
BP	GO:0030647	0.00026597	0.006272566	0.004837575	5
BP	GO:0033212	0.00026597	0.006272566	0.004837575	5
BP	GO:0044598	0.00026597	0.006272566	0.004837575	5
BP	GO:0071372	0.00026597	0.006272566	0.004837575	5
BP	GO:1901653	0.000272911	0.006408748	0.004942602	44
BP	GO:0002430	0.000278904	0.00646656	0.004987189	6
BP	GO:0060099	0.000278904	0.00646656	0.004987189	6
BP	GO:1905153	0.000278904	0.00646656	0.004987189	6
BP	GO:1901655	0.000286325	0.006610737	0.005098382	18
BP	GO:0002673	0.000287543	0.006611083	0.005098649	11
BP	GO:0061458	0.000296929	0.006782586	0.005230917	38
BP	GO:0050890	0.000297836	0.006782586	0.005230917	39
BP	GO:0150146	0.000298705	0.006782586	0.005230917	8
BP	GO:1901661	0.000314563	0.007113273	0.005485952	10
BP	GO:0050804	0.000318994	0.007167964	0.005528131	54
BP	GO:0042359	0.000319591	0.007167964	0.005528131	7
BP	GO:0048762	0.00032392	0.007235538	0.005580246	34
BP	GO:0099177	0.000335246	0.007458199	0.005751968	54
BP	GO:0060562	0.000336802	0.007462615	0.005755374	40
BP	GO:0002819	0.000353593	0.007803198	0.00601804	28
BP	GO:0043086	0.000357184	0.007850896	0.006054826	50
BP	GO:0001818	0.000363893	0.007966494	0.006143979	44
BP	GO:2001237	0.00036794	0.008004622	0.006173384	17
BP	GO:2001236	0.000369846	0.008004622	0.006173384	23
BP	GO:0016049	0.000370004	0.008004622	0.006173384	54
BP	GO:0050767	0.000378493	0.008156147	0.006290244	45
BP	GO:0009064	0.000387473	0.008317057	0.006414343	14
BP	GO:0061041	0.000389125	0.008318063	0.006415118	21
BP	GO:0002675	0.000393575	0.008318063	0.006415118	8

BP	GO:0034698	0.000393575	0.008318063	0.006415118	8
BP	GO:0120255	0.000393575	0.008318063	0.006415118	8
BP	GO:0032612	0.000413824	0.008679251	0.006693676	20
BP	GO:0032652	0.000413824	0.008679251	0.006693676	20
BP	GO:0099024	0.000425063	0.008881063	0.00684932	12
BP	GO:0060541	0.000432919	0.009010948	0.00694949	29
BP	GO:0061138	0.000434806	0.009016073	0.006953443	26
BP	GO:0035584	0.000441518	0.009083324	0.007005308	7
BP	GO:1902644	0.000441518	0.009083324	0.007005308	7
BP	GO:0002696	0.000444661	0.009083324	0.007005308	42
BP	GO:1901342	0.000444661	0.009083324	0.007005308	42
BP	GO:2001233	0.000446505	0.009087201	0.007008299	45
BP	GO:0034350	0.000460451	0.009244396	0.007129532	5
BP	GO:0042373	0.000460451	0.009244396	0.007129532	5
BP	GO:0061370	0.000460451	0.009244396	0.007129532	5
BP	GO:0031099	0.000460958	0.009244396	0.007129532	27
BP	GO:2000177	0.000467204	0.009335591	0.007199864	17
BP	GO:0010518	0.000475406	0.009430885	0.007273357	10
BP	GO:0042554	0.000475406	0.009430885	0.007273357	10
BP	GO:0006956	0.000480126	0.00949026	0.007319149	13
BP	GO:0010043	0.000502807	0.009902952	0.007637428	11
BP	GO:0045765	0.000505708	0.009924069	0.007653714	41
BP	GO:0001704	0.000507491	0.009924069	0.007653714	20
BP	GO:0043523	0.00053236	0.01033682	0.007972039	31
BP	GO:2001234	0.00053236	0.01033682	0.007972039	31
BP	GO:0030278	0.000538946	0.010427854	0.008042247	19
BP	GO:0009266	0.000576717	0.011112711	0.008570428	24
BP	GO:0120178	0.000578387	0.011112711	0.008570428	10
BP	GO:0048592	0.000581526	0.011134098	0.008586922	23
BP	GO:0001706	0.000588509	0.011227123	0.008658665	12
BP	GO:0061045	0.000590471	0.011227123	0.008658665	14
BP	GO:0071229	0.000602239	0.01141139	0.008800777	16
BP	GO:0002703	0.000611996	0.011540088	0.008900033	31
BP	GO:0036005	0.000613231	0.011540088	0.008900033	6
BP	GO:0050680	0.000627626	0.011770672	0.009077865	24
BP	GO:0098742	0.000631602	0.01180495	0.009104301	34
BP	GO:0071496	0.000654092	0.012145294	0.009366784	39
BP	GO:0060348	0.000654232	0.012145294	0.009366784	28
BP	GO:0043434	0.000661959	0.01224735	0.009445492	48
BP	GO:0046942	0.000671264	0.012374732	0.009543733	41
BP	GO:0001906	0.000674166	0.012374732	0.009543733	29
BP	GO:0042509	0.0006756	0.012374732	0.009543733	14
BP	GO:0070665	0.000692	0.012608908	0.009724336	23
BP	GO:0060485	0.000692974	0.012608908	0.009724336	39
BP	GO:0097028	0.000699137	0.012679072	0.009778448	10
BP	GO:0002064	0.00070432	0.012706246	0.009799405	28
BP	GO:0015849	0.000709622	0.012706246	0.009799405	41
BP	GO:0097305	0.000710317	0.012706246	0.009799405	32
BP	GO:0060193	0.000710634	0.012706246	0.009799405	11
BP	GO:0001667	0.000712197	0.012706246	0.009799405	53
BP	GO:1903037	0.00071854	0.012777913	0.009854677	43
BP	GO:0001952	0.000732292	0.012980472	0.010010896	19

BP	GO:0097306	0.000736397	0.013011255	0.010034637	17
BP	GO:0050918	0.000743819	0.013087131	0.010093154	13
BP	GO:0045628	0.000745454	0.013087131	0.010093154	5
BP	GO:0002062	0.000779504	0.013641322	0.010520562	18
BP	GO:0002573	0.000792462	0.01378364	0.010630321	30
BP	GO:0034114	0.000795162	0.01378364	0.010630321	7
BP	GO:0050855	0.000795162	0.01378364	0.010630321	7
BP	GO:0032635	0.000804567	0.013859232	0.01068862	24
BP	GO:0032675	0.000804567	0.013859232	0.01068862	24
BP	GO:0034762	0.000810571	0.013919031	0.010734738	52
BP	GO:0030593	0.00082141	0.013972192	0.010775738	17
BP	GO:0010977	0.000832357	0.013972192	0.010775738	21
BP	GO:0001516	0.000833034	0.013972192	0.010775738	8
BP	GO:0010575	0.000833034	0.013972192	0.010775738	8
BP	GO:0036336	0.000833034	0.013972192	0.010775738	8
BP	GO:0042744	0.000833034	0.013972192	0.010775738	8
BP	GO:0046457	0.000833034	0.013972192	0.010775738	8
BP	GO:0022898	0.000834009	0.013972192	0.010775738	29
BP	GO:0030900	0.000840759	0.014042461	0.010829931	45
BP	GO:0003208	0.000855106	0.014238803	0.010981355	13
BP	GO:0006957	0.000867496	0.014272131	0.011007059	6
BP	GO:0019373	0.000867496	0.014272131	0.011007059	6
BP	GO:0071371	0.000867496	0.014272131	0.011007059	6
BP	GO:2000811	0.000867496	0.014272131	0.011007059	6
BP	GO:0030098	0.00087493	0.014351469	0.011068246	47
BP	GO:0003231	0.000892033	0.01453723	0.01121151	19
BP	GO:0030282	0.000892033	0.01453723	0.01121151	19
BP	GO:0097191	0.000894192	0.01453723	0.01121151	29
BP	GO:0048754	0.000904973	0.014669103	0.011313215	22
BP	GO:1901890	0.00091466	0.014754756	0.011379273	17
BP	GO:0051216	0.000915627	0.014754756	0.011379273	27
BP	GO:0042742	0.000935755	0.015035011	0.011595413	38
BP	GO:0097242	0.000961383	0.015401741	0.011878245	9
BP	GO:0002067	0.000980023	0.015602275	0.012032902	13
BP	GO:0042698	0.000980023	0.015602275	0.012032902	13
BP	GO:0002448	0.000985257	0.015602275	0.012032902	11
BP	GO:0002686	0.000985257	0.015602275	0.012032902	11
BP	GO:0062012	0.000990041	0.015632979	0.012056582	38
BP	GO:0010837	0.001003164	0.01579481	0.01218139	10
BP	GO:0032412	0.001008381	0.015831578	0.012209747	28
BP	GO:0032331	0.001040489	0.016196842	0.012491449	7
BP	GO:0042178	0.001040489	0.016196842	0.012491449	7
BP	GO:0050927	0.001040489	0.016196842	0.012491449	7
BP	GO:0033032	0.00104559	0.016230274	0.012517232	8
BP	GO:0032609	0.00105733	0.016320305	0.012586667	18
BP	GO:0032649	0.00105733	0.016320305	0.012586667	18
BP	GO:0072080	0.001068824	0.016451514	0.012687859	16
BP	GO:1990266	0.001080751	0.016588627	0.012793604	19
BP	GO:0033273	0.001107824	0.016956807	0.013077555	15
BP	GO:1903039	0.001112004	0.0169735	0.013090429	33
BP	GO:0007260	0.00112745	0.017161605	0.0132355	14
BP	GO:0036006	0.001144128	0.017271935	0.01332059	5

BP	GO:0060100	0.001144128	0.017271935	0.01332059	5
BP	GO:1905155	0.001144128	0.017271935	0.01332059	5
BP	GO:0002455	0.001152354	0.017348457	0.013379606	11
BP	GO:0008210	0.001167035	0.017473723	0.013476214	9
BP	GO:0045923	0.001167035	0.017473723	0.013476214	9
BP	GO:0048880	0.001171311	0.017490091	0.013488838	44
BP	GO:0050867	0.001181121	0.017588786	0.013564954	42
BP	GO:0001774	0.001191486	0.017606067	0.013578281	10
BP	GO:0043277	0.001191486	0.017606067	0.013578281	10
BP	GO:0032755	0.001191894	0.017606067	0.013578281	16
BP	GO:0098543	0.001195824	0.017616757	0.013586526	6
BP	GO:0010324	0.001239523	0.018072786	0.013938228	12
BP	GO:0010812	0.001239523	0.018072786	0.013938228	12
BP	GO:0045682	0.001239523	0.018072786	0.013938228	12
BP	GO:0015711	0.001239935	0.018072786	0.013938228	48
BP	GO:0009913	0.001263471	0.018352835	0.01415421	30
BP	GO:0002699	0.001265828	0.018352835	0.01415421	31
BP	GO:0042692	0.001269834	0.018362474	0.014161644	46
BP	GO:0051146	0.001283197	0.018452077	0.014230748	36
BP	GO:0046660	0.001286105	0.018452077	0.014230748	18
BP	GO:0072009	0.001286105	0.018452077	0.014230748	18
BP	GO:0006536	0.001299337	0.018593375	0.014339721	8
BP	GO:0007200	0.00132672	0.018909711	0.014583688	16
BP	GO:0007202	0.001341537	0.018909711	0.014583688	7
BP	GO:0018904	0.001341537	0.018909711	0.014583688	7
BP	GO:0033622	0.001341537	0.018909711	0.014583688	7
BP	GO:0050926	0.001341537	0.018909711	0.014583688	7
BP	GO:0002886	0.001342091	0.018909711	0.014583688	11
BP	GO:0016053	0.001377664	0.019361282	0.014931952	38
BP	GO:0042475	0.001390681	0.019494363	0.015034588	15
BP	GO:0036230	0.001407666	0.019682257	0.015179497	10
BP	GO:0062013	0.001422243	0.0198356	0.015297759	20
BP	GO:0006898	0.001434631	0.019860043	0.01531661	31
BP	GO:0002437	0.001434838	0.019860043	0.01531661	14
BP	GO:0046209	0.001434838	0.019860043	0.01531661	14
BP	GO:0070167	0.001474173	0.020353214	0.015696957	16
BP	GO:0048568	0.001490704	0.02052987	0.015833199	48
BP	GO:0018958	0.001527718	0.020916765	0.016131583	17
BP	GO:0045137	0.001528023	0.020916765	0.016131583	29
BP	GO:0007188	0.001530216	0.020916765	0.016131583	30
BP	GO:0150063	0.001546967	0.021093256	0.016267697	43
BP	GO:0001889	0.001550824	0.021093507	0.016267891	20
BP	GO:0009100	0.001597621	0.021513043	0.016591449	42
BP	GO:0002828	0.001599707	0.021513043	0.016591449	8
BP	GO:0006691	0.001599707	0.021513043	0.016591449	8
BP	GO:0010863	0.001599707	0.021513043	0.016591449	8
BP	GO:0006700	0.001611373	0.021513043	0.016591449	6
BP	GO:0051900	0.001611373	0.021513043	0.016591449	6
BP	GO:0007492	0.001612989	0.021513043	0.016591449	14
BP	GO:2001057	0.001612989	0.021513043	0.016591449	14
BP	GO:0006979	0.001624115	0.02160899	0.016665446	43
BP	GO:0061326	0.001635165	0.021703459	0.016738303	16

BP	GO:0009111	0.001681152	0.022040396	0.016998158	5
BP	GO:0034116	0.001681152	0.022040396	0.016998158	5
BP	GO:0050930	0.001681152	0.022040396	0.016998158	5
BP	GO:0042092	0.001684616	0.022040396	0.016998158	9
BP	GO:0042119	0.001684616	0.022040396	0.016998158	9
BP	GO:0060563	0.001684616	0.022040396	0.016998158	9
BP	GO:0035929	0.001706528	0.022074482	0.017024446	7
BP	GO:0042730	0.001706528	0.022074482	0.017024446	7
BP	GO:0051043	0.001706528	0.022074482	0.017024446	7
BP	GO:1903859	0.001706528	0.022074482	0.017024446	7
BP	GO:0010811	0.001707308	0.022074482	0.017024446	18
BP	GO:0051250	0.001744977	0.022508559	0.017359218	22
BP	GO:0045604	0.001798601	0.023091852	0.017809069	11
BP	GO:0090497	0.001798601	0.023091852	0.017809069	11
BP	GO:0048864	0.001809167	0.023119474	0.017830373	14
BP	GO:0071230	0.001809167	0.023119474	0.017830373	14
BP	GO:0050679	0.00183794	0.023432669	0.018071917	27
BP	GO:0006809	0.001856363	0.023558227	0.018168751	13
BP	GO:0032729	0.001856363	0.023558227	0.018168751	13
BP	GO:0008217	0.001873468	0.023720517	0.018293914	24
BP	GO:0002269	0.001935604	0.024394824	0.018813957	10
BP	GO:0035987	0.001935604	0.024394824	0.018813957	10
BP	GO:0030224	0.001952473	0.024551121	0.018934498	8
BP	GO:0061008	0.001997414	0.025035986	0.019308439	20
BP	GO:0002532	0.00200163	0.025035986	0.019308439	16
BP	GO:0016266	0.002004701	0.025035986	0.019308439	9
BP	GO:0061351	0.002037139	0.025383394	0.01957637	22
BP	GO:0014009	0.002070254	0.025679566	0.019804786	11
BP	GO:0043030	0.002070254	0.025679566	0.019804786	11
BP	GO:0009914	0.002100605	0.025997357	0.020049875	36
BP	GO:0007548	0.002122238	0.026102298	0.020130808	33
BP	GO:0061448	0.002122238	0.026102298	0.020130808	33
BP	GO:0045056	0.002128085	0.026102298	0.020130808	6
BP	GO:0060973	0.002128085	0.026102298	0.020130808	6
BP	GO:0021544	0.002144179	0.02612475	0.020148124	7
BP	GO:0035116	0.002144179	0.02612475	0.020148124	7
BP	GO:0090025	0.002144179	0.02612475	0.020148124	7
BP	GO:0046879	0.002151749	0.026158985	0.020174527	35
BP	GO:0007369	0.002200626	0.026694128	0.020587244	25
BP	GO:0008406	0.00223813	0.027089257	0.020891978	28
BP	GO:0043303	0.002253782	0.027218755	0.02099185	10
BP	GO:0015718	0.002268188	0.027332654	0.021079693	23
BP	GO:0072006	0.002280495	0.027353433	0.021095718	21
BP	GO:0007178	0.002283875	0.027353433	0.021095718	44
BP	GO:0042113	0.002284845	0.027353433	0.021095718	32
BP	GO:0070663	0.002319811	0.027711654	0.021371988	31
BP	GO:0031345	0.002356011	0.027911744	0.021526303	25
BP	GO:1900274	0.002363726	0.027911744	0.021526303	8
BP	GO:2000352	0.002363726	0.027911744	0.021526303	8
BP	GO:1905521	0.002371468	0.027911744	0.021526303	9
BP	GO:0008347	0.002374302	0.027911744	0.021526303	11
BP	GO:0009404	0.002382276	0.027911744	0.021526303	5

BP	GO:0016322	0.002382276	0.027911744	0.021526303	5
BP	GO:0060572	0.002382276	0.027911744	0.021526303	5
BP	GO:0070207	0.002382276	0.027911744	0.021526303	5
BP	GO:0050922	0.002397994	0.028036127	0.02162223	12
BP	GO:0090130	0.002434656	0.028404314	0.021906186	41
BP	GO:0040013	0.002514097	0.029268988	0.022573046	45
BP	GO:2000146	0.002579031	0.029961475	0.023107111	42
BP	GO:0033674	0.002588529	0.030008364	0.023143274	34
BP	GO:0002066	0.002612654	0.030097554	0.023212059	10
BP	GO:0007157	0.002612654	0.030097554	0.023212059	10
BP	GO:0032330	0.002612654	0.030097554	0.023212059	10
BP	GO:0060349	0.002624955	0.030176005	0.023272563	15
BP	GO:0014032	0.002639137	0.030271192	0.023345974	13
BP	GO:0051402	0.002648836	0.030271192	0.023345974	33
BP	GO:0003401	0.002663651	0.030271192	0.023345974	7
BP	GO:0007263	0.002663651	0.030271192	0.023345974	7
BP	GO:0002822	0.002665866	0.030271192	0.023345974	24
BP	GO:0098773	0.002666289	0.030271192	0.023345974	18
BP	GO:0002460	0.002675833	0.030316907	0.02338123	41
BP	GO:0098739	0.002694925	0.030429885	0.023468362	25
BP	GO:0071677	0.002711802	0.030429885	0.023468362	12
BP	GO:0000768	0.002713493	0.030429885	0.023468362	11
BP	GO:0140253	0.002713493	0.030429885	0.023468362	11
BP	GO:2000351	0.002713493	0.030429885	0.023468362	11
BP	GO:0002705	0.002758037	0.030706907	0.023682009	20
BP	GO:0021756	0.002760548	0.030706907	0.023682009	6
BP	GO:0030449	0.002760548	0.030706907	0.023682009	6
BP	GO:0042953	0.002760548	0.030706907	0.023682009	6
BP	GO:1901606	0.002802513	0.031110723	0.023993443	14
BP	GO:0010092	0.002839855	0.031461698	0.024264125	8
BP	GO:0030324	0.002854428	0.031559518	0.024339566	24
BP	GO:0044703	0.002879331	0.031770926	0.02450261	25
BP	GO:0050864	0.002903992	0.031978827	0.024662948	18
BP	GO:0032611	0.00294192	0.032267165	0.024885323	16
BP	GO:0032651	0.00294192	0.032267165	0.024885323	16
BP	GO:0090092	0.002952279	0.03231628	0.024923202	35
BP	GO:0032409	0.002966472	0.032407086	0.024993234	30
BP	GO:0070661	0.002972729	0.032411006	0.024996257	38
BP	GO:0038093	0.00301583	0.032750957	0.025258436	10
BP	GO:1904645	0.00301583	0.032750957	0.025258436	10
BP	GO:0051347	0.003048359	0.033036437	0.025478606	40
BP	GO:0002040	0.003054142	0.033036437	0.025478606	24
BP	GO:0051965	0.003090695	0.033366152	0.025732892	11
BP	GO:0006702	0.003147766	0.03352127	0.025852523	4
BP	GO:0034351	0.003147766	0.03352127	0.025852523	4
BP	GO:0050861	0.003147766	0.03352127	0.025852523	4
BP	GO:0071578	0.003147766	0.03352127	0.025852523	4
BP	GO:0097267	0.003147766	0.03352127	0.025852523	4
BP	GO:1901329	0.003147766	0.03352127	0.025852523	4
BP	GO:2000425	0.003147766	0.03352127	0.025852523	4
BP	GO:0120162	0.003197417	0.033822092	0.026084525	15
BP	GO:0035296	0.003221369	0.033822092	0.026084525	20

BP	GO:0097746	0.003221369	0.033822092	0.026084525	20
BP	GO:0030203	0.003226824	0.033822092	0.026084525	16
BP	GO:0002468	0.003273866	0.033822092	0.026084525	5
BP	GO:0016045	0.003273866	0.033822092	0.026084525	5
BP	GO:0030810	0.003273866	0.033822092	0.026084525	5
BP	GO:0034349	0.003273866	0.033822092	0.026084525	5
BP	GO:0042448	0.003273866	0.033822092	0.026084525	5
BP	GO:0050665	0.003273866	0.033822092	0.026084525	5
BP	GO:0051770	0.003273866	0.033822092	0.026084525	5
BP	GO:0071895	0.003273866	0.033822092	0.026084525	5
BP	GO:1900373	0.003273866	0.033822092	0.026084525	5
BP	GO:0002438	0.003274496	0.033822092	0.026084525	7
BP	GO:0008209	0.003274496	0.033822092	0.026084525	7
BP	GO:0043032	0.003274496	0.033822092	0.026084525	7
BP	GO:0007405	0.003297309	0.033993836	0.026216979	13
BP	GO:0045936	0.003345803	0.034364833	0.026503102	40
BP	GO:0090132	0.003345803	0.034364833	0.026503102	40
BP	GO:0031281	0.003387519	0.034728386	0.026783484	8
BP	GO:0045665	0.003439465	0.035195274	0.027143561	12
BP	GO:0002279	0.003467068	0.035270543	0.02720161	10
BP	GO:0035150	0.003476374	0.035270543	0.02720161	20
BP	GO:0006022	0.003501324	0.035270543	0.02720161	17
BP	GO:0010563	0.003503636	0.035270543	0.02720161	40
BP	GO:0022617	0.003508891	0.035270543	0.02720161	11
BP	GO:0030888	0.003508891	0.035270543	0.02720161	11
BP	GO:0034612	0.003516372	0.035270543	0.02720161	30
BP	GO:0031290	0.003523845	0.035270543	0.02720161	6
BP	GO:0044872	0.003523845	0.035270543	0.02720161	6
BP	GO:0045723	0.003523845	0.035270543	0.02720161	6
BP	GO:0046058	0.003523845	0.035270543	0.02720161	6
BP	GO:0060143	0.003523845	0.035270543	0.02720161	6
BP	GO:0046822	0.003534104	0.035308909	0.027231199	16
BP	GO:0050671	0.003610407	0.036005783	0.027768648	19
BP	GO:0021537	0.0036379	0.036214237	0.027929413	31
BP	GO:0014902	0.003725311	0.036772589	0.028360029	18
BP	GO:0043271	0.003725311	0.036772589	0.028360029	18
BP	GO:0030323	0.003725383	0.036772589	0.028360029	24
BP	GO:0007416	0.003727449	0.036772589	0.028360029	25
BP	GO:0050851	0.003727449	0.036772589	0.028360029	25
BP	GO:0006509	0.003799196	0.037413229	0.028854108	9
BP	GO:0014033	0.003810168	0.037454158	0.028885674	14
BP	GO:0030336	0.003838513	0.037665412	0.029048599	40
BP	GO:0046394	0.00386404	0.037848307	0.029189652	36
BP	GO:0010565	0.003902437	0.038156394	0.029427258	19
BP	GO:0062197	0.003924186	0.038232981	0.029486324	34
BP	GO:0071375	0.003924186	0.038232981	0.029486324	34
BP	GO:0002931	0.00397117	0.038554022	0.02973392	11
BP	GO:0090303	0.00397117	0.038554022	0.02973392	11
BP	GO:0060070	0.003995519	0.038624925	0.029788602	35
BP	GO:0016055	0.004005341	0.038624925	0.029788602	47
BP	GO:0046883	0.004010522	0.038624925	0.029788602	29
BP	GO:0033028	0.004013618	0.038624925	0.029788602	8

BP	GO:0051349	0.004013618	0.038624925	0.029788602	8
BP	GO:0032418	0.004083013	0.039155599	0.030197872	13
BP	GO:1990849	0.004083013	0.039155599	0.030197872	13
BP	GO:0045165	0.004161183	0.039835716	0.030722397	32
BP	GO:0030216	0.004196568	0.040104594	0.030929763	22
BP	GO:0043648	0.004205103	0.040116394	0.030938864	14
BP	GO:0001823	0.004245095	0.040427726	0.031178971	15
BP	GO:0001666	0.004337475	0.041203754	0.031777465	34
BP	GO:0198738	0.004341578	0.041203754	0.031777465	47
BP	GO:0002449	0.004371525	0.041306418	0.031856643	39
BP	GO:0032488	0.004382464	0.041306418	0.031856643	5
BP	GO:0033631	0.004382464	0.041306418	0.031856643	5
BP	GO:0070206	0.004382464	0.041306418	0.031856643	5
BP	GO:0003148	0.004433406	0.041431235	0.031952905	6
BP	GO:0010839	0.004433406	0.041431235	0.031952905	6
BP	GO:0040037	0.004433406	0.041431235	0.031952905	6
BP	GO:0060571	0.004433406	0.041431235	0.031952905	6
BP	GO:2000209	0.004433406	0.041431235	0.031952905	6
BP	GO:0006949	0.004480725	0.041660884	0.032130017	11
BP	GO:0031100	0.004480725	0.041660884	0.032130017	11
BP	GO:1901224	0.004480725	0.041660884	0.032130017	11
BP	GO:0001755	0.004529453	0.041690694	0.032153007	10
BP	GO:0006749	0.004529453	0.041690694	0.032153007	10
BP	GO:0031663	0.004529453	0.041690694	0.032153007	10
BP	GO:0060986	0.004529453	0.041690694	0.032153007	10
BP	GO:0070542	0.004529453	0.041690694	0.032153007	10
BP	GO:1901616	0.004529453	0.041690694	0.032153007	10
BP	GO:0032946	0.00454535	0.041697323	0.03215812	19
BP	GO:0051592	0.00454535	0.041697323	0.03215812	19
BP	GO:0006066	0.004573136	0.041743159	0.032193469	39
BP	GO:0044282	0.004573136	0.041743159	0.032193469	39
BP	GO:0090287	0.004573136	0.041743159	0.032193469	39
BP	GO:1904035	0.004603345	0.041949219	0.032352388	16
BP	GO:0060395	0.004632599	0.042076248	0.032450356	14
BP	GO:0071901	0.004632599	0.042076248	0.032450356	14
BP	GO:0003139	0.004683375	0.042258037	0.032590558	4
BP	GO:0060601	0.004683375	0.042258037	0.032590558	4
BP	GO:0072178	0.004683375	0.042258037	0.032590558	4
BP	GO:0099550	0.004683375	0.042258037	0.032590558	4
BP	GO:0043269	0.004701687	0.042284398	0.032610888	47
BP	GO:0045859	0.004701687	0.042284398	0.032610888	47
BP	GO:0048246	0.004725264	0.042357792	0.032667491	8
BP	GO:0150077	0.004725264	0.042357792	0.032667491	8
BP	GO:0044706	0.004776418	0.042746609	0.032967357	25
BP	GO:0034694	0.004810068	0.042781554	0.032994308	7
BP	GO:0061037	0.004810068	0.042781554	0.032994308	7
BP	GO:0006023	0.004818421	0.042781554	0.032994308	12
BP	GO:1902107	0.004819251	0.042781554	0.032994308	23
BP	GO:1903708	0.004819251	0.042781554	0.032994308	23
BP	GO:0022604	0.004839431	0.042891413	0.033079034	28
BP	GO:0170039	0.004898153	0.043341946	0.033426498	19
BP	GO:0010631	0.005000139	0.044173252	0.034067624	39

BP	GO:0048678	0.005013342	0.044181302	0.034073832	13
BP	GO:0007189	0.005017131	0.044181302	0.034073832	20
BP	GO:0042326	0.005026229	0.044190604	0.034081006	34
BP	GO:0034764	0.00507405	0.04432581	0.034185281	25
BP	GO:0030195	0.00507447	0.04432581	0.034185281	9
BP	GO:0033628	0.00507447	0.04432581	0.034185281	9
BP	GO:0042551	0.00507447	0.04432581	0.034185281	9
BP	GO:0046545	0.005087518	0.04432581	0.034185281	15
BP	GO:0051251	0.005090007	0.04432581	0.034185281	35
BP	GO:0090288	0.005110613	0.044434842	0.034269369	18
BP	GO:0006575	0.005138416	0.044555135	0.034362143	23
BP	GO:0042306	0.005148774	0.044555135	0.034362143	10
BP	GO:0061005	0.005148774	0.044555135	0.034362143	10
BP	GO:0003014	0.005299288	0.045713641	0.035255614	17
BP	GO:0003206	0.005299288	0.045713641	0.035255614	17
BP	GO:0045669	0.005365262	0.046210211	0.035638582	12
BP	GO:0002070	0.005504854	0.047110403	0.036332835	6
BP	GO:0007190	0.005504854	0.047110403	0.036332835	6
BP	GO:0051882	0.005504854	0.047110403	0.036332835	6
BP	GO:0071676	0.005504854	0.047110403	0.036332835	6
BP	GO:0070555	0.005515941	0.047110403	0.036332835	18
BP	GO:0050670	0.005523492	0.047110403	0.036332835	27
BP	GO:0008543	0.005538366	0.047110403	0.036332835	13
BP	GO:0071277	0.005538366	0.047110403	0.036332835	13
BP	GO:0030316	0.005556818	0.047194307	0.036397544	15
BP	GO:0048638	0.005593679	0.04743405	0.03658244	35
BP	GO:0045576	0.005654913	0.047615783	0.036722598	11
BP	GO:0051926	0.005654913	0.047615783	0.036722598	11
BP	GO:0072577	0.005654913	0.047615783	0.036722598	11
BP	GO:0023061	0.005658461	0.047615783	0.036722598	48
BP	GO:2000377	0.005671442	0.047615783	0.036722598	19
BP	GO:0045619	0.005715769	0.047615783	0.036722598	25
BP	GO:1901888	0.005715769	0.047615783	0.036722598	25
BP	GO:0002524	0.005734375	0.047615783	0.036722598	5
BP	GO:0010755	0.005734375	0.047615783	0.036722598	5
BP	GO:0042481	0.005734375	0.047615783	0.036722598	5
BP	GO:0045064	0.005734375	0.047615783	0.036722598	5
BP	GO:0045346	0.005734375	0.047615783	0.036722598	5
BP	GO:0051917	0.005734375	0.047615783	0.036722598	5
BP	GO:0002761	0.005736424	0.047615783	0.036722598	17
BP	GO:0045671	0.005755265	0.047700122	0.036787643	7
BP	GO:0043154	0.005825159	0.047975121	0.03699973	9
BP	GO:1900047	0.005825159	0.047975121	0.03699973	9
BP	GO:0003229	0.005832482	0.047975121	0.03699973	10
BP	GO:0045620	0.005832482	0.047975121	0.03699973	10
BP	GO:1903409	0.005832482	0.047975121	0.03699973	10
BP	GO:0048588	0.005840829	0.047975121	0.03699973	27
BP	GO:0050830	0.005922945	0.048576988	0.037463906	16
BP	GO:0001933	0.005934018	0.048595277	0.037478011	32
BP	GO:0001101	0.00594715	0.048630342	0.037505053	18
BP	GO:0046546	0.006093997	0.049757076	0.038374022	19
CC	GO:0062023	1.70E-12	9.05E-10	8.27E-10	71

CC	GO:0031091	2.60E-08	6.92E-06	6.33E-06	23
CC	GO:0031093	3.12E-07	5.54E-05	5.06E-05	18
CC	GO:0009897	6.29E-07	6.92E-05	6.33E-05	55
CC	GO:0045177	6.51E-07	6.92E-05	6.33E-05	61
CC	GO:0016324	1.12E-06	9.92E-05	9.07E-05	54
CC	GO:0005788	7.67E-06	0.000582709	0.000532671	43
CC	GO:0031983	2.12E-05	0.001395793	0.001275932	43
CC	GO:0030667	2.36E-05	0.001395793	0.001275932	42
CC	GO:0034774	3.43E-05	0.001823878	0.001667257	42
CC	GO:0060205	4.26E-05	0.002062307	0.001885212	42
CC	GO:0009925	5.33E-05	0.002179477	0.001992319	38
CC	GO:0045121	5.33E-05	0.002179477	0.001992319	38
CC	GO:0098857	5.75E-05	0.002183727	0.001996204	38
CC	GO:0045178	9.92E-05	0.003517618	0.003215551	39
CC	GO:0016323	0.00012022	0.00399733	0.003654069	34
CC	GO:0042581	0.000198686	0.006217704	0.005683774	24
CC	GO:0031253	0.000288301	0.008520904	0.007789192	42
CC	GO:0042599	0.000618205	0.017155382	0.015682205	6
CC	GO:0031045	0.000644939	0.017155382	0.015682205	9
CC	GO:0070820	0.000707779	0.017930411	0.01639068	23
CC	GO:0043020	0.000874447	0.021145723	0.019329886	6
CC	GO:0098992	0.001049874	0.024284047	0.022198713	7
CC	GO:0030139	0.001542853	0.034199917	0.031263082	39
CC	GO:0072562	0.001721843	0.03664081	0.033494369	20
CC	GO:0005581	0.001836061	0.037568627	0.034342512	14
CC	GO:0001533	0.002095785	0.041294722	0.037748638	11
CC	GO:0033162	0.002781598	0.049327009	0.045091172	6
CC	GO:0045009	0.002781598	0.049327009	0.045091172	6
CC	GO:0090741	0.002781598	0.049327009	0.045091172	6
MF	GO:0005201	3.27E-09	3.37E-06	2.93E-06	35
MF	GO:0016209	2.77E-07	0.000133149	0.000116072	21
MF	GO:0020037	5.16E-07	0.000133149	0.000116072	28
MF	GO:0005539	6.34E-07	0.000133149	0.000116072	39
MF	GO:0046906	6.69E-07	0.000133149	0.000116072	29
MF	GO:0004497	7.76E-07	0.000133149	0.000116072	23
MF	GO:0015020	1.72E-06	0.000221108	0.000192749	12
MF	GO:0038187	1.72E-06	0.000221108	0.000192749	12
MF	GO:0061135	1.97E-06	0.000225197	0.000196313	32
MF	GO:0005125	3.96E-06	0.00040772	0.000355427	37
MF	GO:0030414	4.94E-06	0.000408766	0.000356339	30
MF	GO:0070330	5.19E-06	0.000408766	0.000356339	10
MF	GO:0004857	5.26E-06	0.000408766	0.000356339	52
MF	GO:0061134	5.56E-06	0.000408766	0.000356339	36
MF	GO:0004866	6.17E-06	0.000419814	0.00036597	29
MF	GO:0016709	6.52E-06	0.000419814	0.00036597	12
MF	GO:0016705	7.01E-06	0.000424621	0.00037016	30
MF	GO:0005506	7.48E-06	0.000428102	0.000373194	27
MF	GO:1901681	1.01E-05	0.000545953	0.00047593	39
MF	GO:0140375	1.72E-05	0.000886689	0.000772965	26
MF	GO:0004867	1.98E-05	0.000930437	0.000811101	20
MF	GO:0016614	2.08E-05	0.000930437	0.000811101	24
MF	GO:0019838	2.08E-05	0.000930437	0.000811101	24

MF	GO:0030283	3.98E-05	0.001708252	0.001489156	6
MF	GO:0016684	4.31E-05	0.001774509	0.001546915	14
MF	GO:0004601	0.000124052	0.004811848	0.004194692	13
MF	GO:0043394	0.000130171	0.004811848	0.004194692	10
MF	GO:0019763	0.000130808	0.004811848	0.004194692	6
MF	GO:0008194	0.000186751	0.006632879	0.005782162	23
MF	GO:0008201	0.000199313	0.006843082	0.005965405	26
MF	GO:0050661	0.000241197	0.007795806	0.006795935	12
MF	GO:0001540	0.0002422	0.007795806	0.006795935	16
MF	GO:0001664	0.000317374	0.009905918	0.00863541	37
MF	GO:0005044	0.00037916	0.011486306	0.010013101	8
MF	GO:0008083	0.000392046	0.011537347	0.010057595	24
MF	GO:0016616	0.000425608	0.012177126	0.010615318	20
MF	GO:0038024	0.000476905	0.013275992	0.011573246	14
MF	GO:0005543	0.000546014	0.014799854	0.012901661	54
MF	GO:0005178	0.000602494	0.015740782	0.013721908	23
MF	GO:0004713	0.000615995	0.015740782	0.013721908	21
MF	GO:0016712	0.000626575	0.015740782	0.013721908	10
MF	GO:0008106	0.000738062	0.017306234	0.015086579	7
MF	GO:0030169	0.000739295	0.017306234	0.015086579	6
MF	GO:0034185	0.000739295	0.017306234	0.015086579	6
MF	GO:0005126	0.000788864	0.018056228	0.01574038	34
MF	GO:0030291	0.000821342	0.018390918	0.016032145	9
MF	GO:0004032	0.000875543	0.019187423	0.016726491	5
MF	GO:0001786	0.000894293	0.019190047	0.016728779	13
MF	GO:0008391	0.00104361	0.02149837	0.018741042	6
MF	GO:0043395	0.00104361	0.02149837	0.018741042	6
MF	GO:0016757	0.001135246	0.022927507	0.019986882	33
MF	GO:0001784	0.00131046	0.025957198	0.022627992	10
MF	GO:0033293	0.001393423	0.02688391	0.023435846	14
MF	GO:0043177	0.001423473	0.02688391	0.023435846	27
MF	GO:0004303	0.001435549	0.02688391	0.023435846	6
MF	GO:0072341	0.001713539	0.031516874	0.027474598	16
MF	GO:0019825	0.001999789	0.036136538	0.031501754	8
MF	GO:0016758	0.002661682	0.046952938	0.04093087	25
MF	GO:0004714	0.002724547	0.046952938	0.04093087	11
MF	GO:0019955	0.002761218	0.046952938	0.04093087	20
MF	GO:0004089	0.002780708	0.046952938	0.04093087	5
