# Supplementary material for: Identification of four key genes related to the diagnosis of chronic obstructive pulmonary disease using bioinformatics analysis
Source: Front Genet. 2025 Mar 5;16:1499996. doi: 10.3389/fgene.2025.1499996 (PMC11919834; doi:10.3389/fgene.2025.1499996)
Supplement: Supplementary file 2 [file Table1.docx]

Supplementary Material

**Supplementary table 1. GEO datasets of COPD for analysis.**

| **GEO ID** | **Application** | **Platform** | **Tissue types** | **Patients** | **Control** |
| --- | --- | --- | --- | --- | --- |
| GSE11906, GSE20257 | training set | GPL570 | airway | 28 | 90 |
| GSE5058, GSE8545 | validation set | GPL570 | airway | 21 | 19 |
| Total |  |  |  | 49 | 109 |
