# Supplementary material for: Identification of four key genes related to the diagnosis of chronic obstructive pulmonary disease using bioinformatics analysis
Source: Front Genet. 2025 Mar 5;16:1499996. doi: 10.3389/fgene.2025.1499996 (PMC11919834; doi:10.3389/fgene.2025.1499996)
Supplement: Supplementary file 3 [file DataSheet3.pdf]

Supplementary Table 4. GSEA Results for Pathway Enrichment

ID	Description	NES	pvalue
hsa04110	Cell cycle	0.663755	2.832899
hsa03050	Proteasome	0.709478	2.45301
hsa04657	IL-17 signaling pathway	0.562209	2.208137
hsa03030	DNA replication	0.722768	2.368337
hsa05169	Epstein-Barr virus infection	0.433501	1.925816
hsa01230	Biosynthesis of amino acids	0.589051	2.181961
hsa05166	Human T-cell leukemia virus 1 infection	0.389361	1.740335
hsa04613	Neutrophil extracellular trap formation	0.449657	1.881672
hsa03008	Ribosome biogenesis in eukaryotes	0.573707	2.09154
hsa04061	Viral protein interaction with cytokine and cytokine receptor	0.498752	1.954787
hsa04974	Protein digestion and absorption	-0.53034	-1.90976
hsa05323	Rheumatoid arthritis	0.496508	1.93402
hsa04510	Focal adhesion	-0.41992	-1.71226
hsa05170	Human immunodeficiency virus 1 infection	0.371115	1.632797
hsa04218	Cellular senescence	0.411595	1.750796
hsa04064	NF-kappa B signaling pathway	0.468828	1.868782
hsa04062	Chemokine signaling pathway	0.376972	1.649462
hsa04060	Cytokine-cytokine receptor interaction	0.339379	1.557508
hsa05340	Primary immunodeficiency	0.622965	2.068996
hsa04820	Cytoskeleton in muscle cells	-0.39919	-1.6355
hsa04114	Oocyte meiosis	0.439685	1.762231
hsa04650	Natural killer cell mediated cytotoxicity	0.410918	1.678382
hsa04115	p53 signaling pathway	0.474384	1.784401
hsa04612	Antigen processing and presentation	0.486356	1.815106
hsa05152	Tuberculosis	0.374515	1.639743
hsa04660	T cell receptor signaling pathway	0.401374	1.662333
hsa04066	HIF-1 signaling pathway	0.415408	1.681585
hsa00982	Drug metabolism - cytochrome P450	-0.53981	-1.78006
hsa04668	TNF signaling pathway	0.408419	1.673696
hsa04151	PI3K-Akt signaling pathway	-0.33661	-1.44463
hsa04620	Toll-like receptor signaling pathway	0.410483	1.655702
hsa05020	Prion disease	0.331599	1.48674
hsa04512	ECM-receptor interaction	-0.46216	-1.66876
hsa03013	Nucleocytoplasmic transport	0.422167	1.682789
hsa05203	Viral carcinogenesis	0.352386	1.541726
hsa05332	Graft-versus-host disease	0.554349	1.841108
hsa05164	Influenza A	0.345505	1.506294
hsa05162	Measles	0.374167	1.560248
hsa01240	Biosynthesis of cofactors	0.385605	1.580205
hsa05219	Bladder cancer	0.521353	1.782726
hsa04914	Progesterone-mediated oocyte maturation	0.428291	1.661015
hsa00350	Tyrosine metabolism	-0.5726	-1.71676
hsa05230	Central carbon metabolism in cancer	0.439316	1.675511
hsa05322	Systemic lupus erythematosus	0.423721	1.624044
hsa01200	Carbon metabolism	0.387377	1.571201
hsa05410	Hypertrophic cardiomyopathy	-0.43741	-1.61432
hsa04210	Apoptosis	0.356037	1.488052
hsa04923	Regulation of lipolysis in adipocytes	-0.49416	-1.66272
hsa05330	Allograft rejection	0.528493	1.731743
hsa04340	Hedgehog signaling pathway	-0.50874	-1.62797
hsa05235	PD-L1 expression and PD-1 checkpoint pathway in cancer	0.404126	1.574171
hsa00270	Cysteine and methionine metabolism	0.479762	1.657449
hsa00970	Aminoacyl-tRNA biosynthesis	0.515062	1.687735
hsa05414	Dilated cardiomyopathy	-0.409	-1.52044
hsa05168	Herpes simplex virus 1 infection	-0.32573	-1.38806
hsa04152	AMPK signaling pathway	-0.39808	-1.49708

hsa04930	Type II diabetes mellitus	-0.49683	-1.58988
hsa04710	Circadian rhythm	-0.55736	-1.66249
hsa03430	Mismatch repair	0.579179	1.696418
hsa05110	Vibrio cholerae infection	0.446157	1.546774
hsa04726	Serotonergic synapse	-0.38374	-1.42423
hsa05160	Hepatitis C	0.330273	1.413859
hsa04360	Axon guidance	-0.35788	-1.42047
hsa00601	Glycosphingolipid biosynthesis - lacto and neolacto series	0.520122	1.593337
hsa03410	Base excision repair	0.474143	1.607775
hsa04814	Motor proteins	0.326676	1.404851
hsa05133	Pertussis	0.408234	1.530922
hsa03440	Homologous recombination	0.475615	1.597241
hsa00430	Taurine and hypotaurine metabolism	-0.67409	-1.61211
hsa00190	Oxidative phosphorylation	0.347621	1.409418
hsa05217	Basal cell carcinoma	-0.43728	-1.46552
hsa03460	Fanconi anemia pathway	0.452859	1.541351
hsa01232	Nucleotide metabolism	0.400337	1.477829
hsa04371	Apelin signaling pathway	-0.3591	-1.36187
hsa00360	Phenylalanine metabolism	-0.65273	-1.63264
hsa04621	NOD-like receptor signaling pathway	0.321082	1.383284
hsa05163	Human cytomegalovirus infection	0.292534	1.30661
hsa04940	Type I diabetes mellitus	0.431682	1.469732
hsa04960	Aldosterone-regulated sodium reabsorption	-0.48226	-1.46712
hsa00980	Metabolism of xenobiotics by cytochrome P450	-0.44758	-1.4938
hsa05012	Parkinson disease	0.288013	1.286779
hsa04211	Longevity regulating pathway	-0.39812	-1.42353
hsa04020	Calcium signaling pathway	-0.3156	-1.30157
hsa00100	Steroid biosynthesis	0.56033	1.591798
hsa04145	Phagosome	0.312813	1.319156
hsa04022	cGMP-PKG signaling pathway	-0.34034	-1.33638
hsa04610	Complement and coagulation cascades	-0.39638	-1.42735
hsa04622	RIG-I-like receptor signaling pathway	0.356108	1.339506
hsa05204	Chemical carcinogenesis - DNA adducts	-0.45258	-1.48452
hsa04640	Hematopoietic cell lineage	0.342755	1.364783
hsa04910	Insulin signaling pathway	-0.34382	-1.31341
hsa04010	MAPK signaling pathway	-0.29684	-1.24954
hsa05320	Autoimmune thyroid disease	0.407263	1.413486
hsa04350	TGF-beta signaling pathway	-0.3685	-1.3672
hsa00830	Retinol metabolism	-0.44361	-1.41957
hsa05417	Lipid and atherosclerosis	0.279378	1.241873
hsa04625	C-type lectin receptor signaling pathway	0.32997	1.31901
hsa01523	Antifolate resistance	0.481248	1.496051
hsa00520	Amino sugar and nucleotide sugar metabolism	0.422391	1.444332
hsa04920	Adipocytokine signaling pathway	-0.40011	-1.39335
hsa04724	Glutamatergic synapse	-0.36459	-1.35267
hsa00650	Butanoate metabolism	-0.53444	-1.46026
