# Supplementary material for: Identification of four key genes related to the diagnosis of chronic obstructive pulmonary disease using bioinformatics analysis
Source: Front Genet. 2025 Mar 5;16:1499996. doi: 10.3389/fgene.2025.1499996 (PMC11919834; doi:10.3389/fgene.2025.1499996)
Supplement: Supplementary file 4 [file DataSheet1.pdf]

Supplementary table 2. DEGs

Gene Name	logFC	AveExpr	t	P.Value	adj.P.Val	Regulation
CYP1B1	5.535261783	7.015466632	26.87136574	2.39E-52	5.45E-48	up
NQO1	1.708734319	12.60573994	18.82517768	1.79E-37	2.04E-33	up
ME1	2.084252641	8.873535071	18.70601065	3.15E-37	2.40E-33	up
SLC7A11	3.099939919	7.071525015	18.53375521	7.17E-37	4.10E-33	up
UCHL1	3.896629145	7.163980127	18.34199822	1.80E-36	8.21E-33	up
AKR1B10	4.58760676	8.658349011	17.8165131	2.29E-35	8.70E-32	up
CABYR	2.656244901	7.451911688	16.50991984	1.48E-32	4.82E-29	up
AKR1C3	1.634964819	12.46116615	15.81727864	4.97E-31	1.30E-27	up
MUCL1	2.797540532	10.41178004	15.81184189	5.11E-31	1.30E-27	up
GPX2	2.42449565	9.307416147	15.61245594	1.42E-30	3.24E-27	up
CYP1A1	4.984984089	5.343407278	14.98593935	3.64E-29	7.55E-26	up
AKR1C2	1.742893923	12.09888213	14.83756767	7.89E-29	1.39E-25	up
LOC101930400	1.742893923	12.09888213	14.83756767	7.89E-29	1.39E-25	up
NT5E	-1.401834237	8.158563225	-14.07554636	4.37E-27	7.12E-24	down
AHRR	2.350297842	6.471464641	13.96816092	7.72E-27	1.18E-23	up
LOC284825	2.615116485	7.007315321	13.75672395	2.38E-26	3.40E-23	up
PPP1R16B	-1.695548841	7.892014329	-13.63336793	4.60E-26	6.19E-23	down
C2orf70	1.402274415	8.45659707	13.48441504	1.02E-25	1.30E-22	up
SRXN1	1.249391412	9.440887651	13.36224051	1.97E-25	2.24E-22	up
ALDH3A1	2.374885111	13.0187522	13.35939782	2.00E-25	2.24E-22	up
HTATIP2	1.061604345	9.856585498	13.35365299	2.06E-25	2.24E-22	up
CLDN10	1.358794944	10.31397323	13.345097	2.16E-25	2.24E-22	up
DTNA	1.439427487	6.184131325	12.95419546	1.77E-24	1.75E-21	up
TALDO1	0.980806559	13.35019853	12.9438892	1.88E-24	1.75E-21	up
AKR1C1	1.42084351	11.92005732	12.94034969	1.91E-24	1.75E-21	up
TXNRD1	1.087651081	11.4806913	12.39261336	3.73E-23	3.28E-20	up
PIR	1.313850624	10.9402926	12.31024316	5.85E-23	4.94E-20	up
SPP1	3.234061335	7.683140045	12.12168004	1.63E-22	1.33E-19	up
PHLDA1	1.158666764	6.614817893	12.09724339	1.87E-22	1.47E-19	up
ELFN2	1.618254866	6.658095659	12.06464474	2.23E-22	1.70E-19	up
ST3GAL4-AS1	1.54179223	6.297698905	11.91055644	5.18E-22	3.82E-19	up
GAD1	2.312830789	5.980446387	11.85786634	6.91E-22	4.93E-19	up
AKR1B1	1.045543547	10.37946323	11.73877479	1.33E-21	9.18E-19	up
SLC2A1	0.998523532	8.873848073	11.70624587	1.59E-21	1.06E-18	up
TMEM200C	1.183755561	6.785260081	11.54576177	3.82E-21	2.49E-18	up
C3	-1.898841698	12.82853689	-10.92867663	1.13E-19	7.06E-17	down
PLK2	-1.325660883	8.825108569	-10.92689881	1.14E-19	7.06E-17	down
CLIP4	1.241518416	9.446468957	10.87643248	1.51E-19	9.07E-17	up
SLITRK6	-1.192876866	10.90976106	-10.85227945	1.72E-19	1.01E-16	down
STEAP4	-1.13584155	11.35112477	-10.63380931	5.73E-19	3.27E-16	down
GLI3	-0.727840686	8.304151236	-10.6061774	6.67E-19	3.71E-16	down
EPB41L2	-0.892715798	7.303448206	-10.53410447	9.91E-19	5.39E-16	down
CX3CL1	-1.612888687	9.489476488	-10.43619178	1.70E-18	9.01E-16	down
CEACAM6	1.0557536	13.58291988	10.41810547	1.87E-18	9.73E-16	up
ELMOD1	2.380686543	6.447962447	10.38067336	2.30E-18	1.14E-15	up
LOC643923	2.380686543	6.447962447	10.38067336	2.30E-18	1.14E-15	up
PHEX	1.587656945	7.534493453	10.23738432	5.06E-18	2.46E-15	up
PLEKHF2	-0.713441671	11.13141954	-10.11370955	9.98E-18	4.71E-15	down
VPS13D	0.718255676	8.824355388	10.11136962	1.01E-17	4.71E-15	up
PLPP5	0.546851249	10.6051125	10.04703987	1.44E-17	6.57E-15	up
MIR1204	0.817839626	6.932984167	9.987571966	1.99E-17	8.93E-15	up
CBR3	1.347923896	8.92710892	9.955737427	2.37E-17	1.04E-14	up
FKBP11	0.931473997	9.953406396	9.926070569	2.79E-17	1.20E-14	up
MAP1B	0.965455532	10.35457014	9.894095436	3.33E-17	1.41E-14	up
FAM65C	-1.562453597	6.12746733	-9.819400193	5.01E-17	2.08E-14	down
LOC729970	-0.808106014	7.259733765	-9.783447041	6.10E-17	2.49E-14	down
MAOB	-1.261096603	9.172213669	-9.749368681	7.35E-17	2.95E-14	down
PVT1	0.757800453	6.739514486	9.565398996	2.01E-16	7.92E-14	up
THSD7A	-1.296894655	6.37652329	-9.556742809	2.11E-16	8.16E-14	down

FMO2	-1.126651501	11.24159321	-9.543999953	2.26E-16	8.60E-14	down
TMEM45B	0.826810489	11.82814642	9.497178516	2.92E-16	1.09E-13	up
C2CD3	0.559239978	9.551418526	9.451200045	3.75E-16	1.38E-13	up
SHISA9	-1.482874615	6.066073684	-9.444425056	3.89E-16	1.41E-13	down
NMRAL1P1	2.112630575	6.159042157	9.325409003	7.45E-16	2.66E-13	up
ABHD2	0.878632367	11.02941454	9.301888467	8.46E-16	2.97E-13	up
WFDC6	-1.273575988	9.286918147	-9.286630059	9.20E-16	3.18E-13	down
EGF	2.136385978	5.608421341	9.257902067	1.08E-15	3.67E-13	up
SEC14L3	-2.124048551	9.637833539	-9.254163525	1.10E-15	3.69E-13	down
PGRMC1	-0.622518747	12.70242327	-9.242495083	1.17E-15	3.87E-13	down
ITM2A	-1.582808396	9.161811483	-9.220432412	1.32E-15	4.30E-13	down
ABCB6	1.009173113	8.03536445	9.206974295	1.42E-15	4.56E-13	up
CBR1	1.35525802	10.49598315	9.1862148	1.59E-15	5.04E-13	up
TNNT3	0.932579902	8.241603626	9.177220675	1.67E-15	5.22E-13	up
FTH1	0.522183073	14.44768823	9.162070332	1.81E-15	5.59E-13	up
CEACAM5	2.657148319	10.63914079	9.125827346	2.20E-15	6.71E-13	up
DEFB1	1.504650393	8.206379689	9.102116948	2.51E-15	7.53E-13	up
SCGB3A1	-0.855160897	15.33312232	-9.085898326	2.74E-15	8.12E-13	down
CCDC81	-0.992447194	10.92317985	-9.067442831	3.03E-15	8.86E-13	down
CDKN1C	-0.59287612	7.991088839	-9.064110592	3.08E-15	8.91E-13	down
KLHDC7A	-0.902245014	8.801105339	-9.015778908	4.00E-15	1.14E-12	down
CNGB1	1.229451734	5.840820787	8.986968129	4.68E-15	1.32E-12	up
PROS1	-1.287039198	12.75466685	-8.981899461	4.81E-15	1.34E-12	down
CHST15	0.707497824	8.966302497	8.980109669	4.86E-15	1.34E-12	up
PACSIN2	0.521823978	10.84957781	8.959364413	5.44E-15	1.48E-12	up
MSRB1	0.806727658	10.50232825	8.896869482	7.62E-15	2.05E-12	up
TLE1	0.645793925	7.771729488	8.861984099	9.20E-15	2.44E-12	up
MEP1A	1.925020678	4.952419201	8.842670726	1.02E-14	2.66E-12	up
POU2AF1	-1.009167649	10.95497867	-8.842272238	1.02E-14	2.66E-12	down
SIX3	1.29724873	5.177729526	8.827844617	1.11E-14	2.84E-12	up
PANK1	-0.991303714	8.464042122	-8.818888618	1.16E-14	2.95E-12	down
IDS	0.629248142	9.789048424	8.789725873	1.36E-14	3.41E-12	up
YAP1	-0.527084985	10.89615891	-8.776307788	1.46E-14	3.63E-12	down
G6PD	1.16939325	8.210378952	8.75296997	1.66E-14	4.07E-12	up
SFRP2	1.777051901	6.113036497	8.747982125	1.70E-14	4.14E-12	up
MB	-0.88185465	10.30760171	-8.740637298	1.77E-14	4.26E-12	down
SLC29A1	-1.173998609	7.93762685	-8.715198103	2.03E-14	4.82E-12	down
MTHFD2	1.068382661	8.029582959	8.713698452	2.05E-14	4.82E-12	up
AADAT	-0.949640527	8.542476781	-8.697753841	2.23E-14	5.20E-12	down
LRRC31	1.887349845	7.038913774	8.693154398	2.29E-14	5.28E-12	up
DIO1	1.139797688	9.172883641	8.673140636	2.55E-14	5.82E-12	up
CLEC5A	2.647150537	5.182503804	8.662561601	2.70E-14	6.10E-12	up
MMP12	2.075475141	6.687203334	8.627024194	3.27E-14	7.31E-12	up
TLR5	-0.635396975	10.04070241	-8.618762926	3.41E-14	7.53E-12	down
ELL3	0.713827005	10.27551056	8.617969474	3.43E-14	7.53E-12	up
COA6	0.59514296	9.920269618	8.607701285	3.62E-14	7.88E-12	up
S100A10	1.005505718	11.63819562	8.605913802	3.66E-14	7.88E-12	up
GSR	0.785712924	10.43174913	8.583958655	4.12E-14	8.78E-12	up
MT1F	-0.965510567	10.37658761	-8.579143064	4.22E-14	8.93E-12	down
KIAA0430	-0.501448995	9.739736699	-8.539449948	5.23E-14	1.08E-11	down
IRF2BP2	-0.599365709	10.89393682	-8.487925445	6.89E-14	1.42E-11	down
RABEP2	0.566892614	8.921396482	8.47442925	7.40E-14	1.51E-11	up
D2HGDH	-1.079048165	8.784520834	-8.472872654	7.47E-14	1.51E-11	down
SPA17	0.507006797	13.19920322	8.443302433	8.75E-14	1.75E-11	up
APPL2	-0.902252434	11.6908096	-8.397485346	1.12E-13	2.22E-11	down
NEK6	0.800020119	7.651154608	8.358452804	1.38E-13	2.71E-11	up
KIF21A	-0.709332728	11.64174529	-8.33637683	1.55E-13	3.02E-11	down
HGD	1.145570177	7.546317562	8.307918211	1.80E-13	3.49E-11	up
GPAT3	1.364048027	8.705249586	8.283633311	2.05E-13	3.94E-11	up
ADH7	1.898995844	11.21736641	8.265856392	2.25E-13	4.29E-11	up
SYPL1	-0.528356092	11.97532167	-8.263856736	2.28E-13	4.30E-11	down

HRK	1.078350973	6.565959767	8.22935331	2.74E-13	5.13E-11	up
TENM4	0.79711884	8.318243233	8.202529506	3.16E-13	5.86E-11	up
PYCR1	1.037998066	8.113831532	8.178469574	3.59E-13	6.56E-11	up
FAM114A1	0.546313557	10.7759211	8.173640706	3.68E-13	6.67E-11	up
TPRXL	2.16712262	6.692053555	8.161908667	3.92E-13	7.05E-11	up
ASCL3	1.401506784	7.7616311	8.143911498	4.31E-13	7.69E-11	up
TXNDC16	-0.907821502	8.304580018	-8.127433328	4.70E-13	8.33E-11	down
SUSD4	-0.854674574	9.177973358	-8.124509447	4.78E-13	8.39E-11	down
CREB3L4	0.641885787	9.842089353	8.108741327	5.19E-13	9.02E-11	up
IL6ST	-0.688103055	9.799081481	-8.107985912	5.21E-13	9.02E-11	down
GCLM	0.664951811	10.9282027	8.100182021	5.43E-13	9.33E-11	up
GALNT6	1.331031755	10.62832975	8.087900705	5.80E-13	9.88E-11	up
RIMKLA	0.680820024	5.93253917	8.085862853	5.86E-13	9.92E-11	up
PPP1R3B	-0.604959016	7.674892177	-8.081512109	6.00E-13	1.01E-10	down
PNP	0.734883868	9.465213534	8.078117712	6.11E-13	1.02E-10	up
ARFGEF3	-0.578437849	10.58614617	-8.073987559	6.24E-13	1.03E-10	down
NAV1	0.849893633	7.611764066	8.072328505	6.30E-13	1.03E-10	up
CCL2	2.551589985	6.388837823	8.063108105	6.61E-13	1.08E-10	up
FHOD1	0.808238731	8.325341076	8.041977779	7.40E-13	1.20E-10	up
FUBP1	-0.56330931	8.997018014	-8.01097661	8.71E-13	1.39E-10	down
CCDC184	1.144624385	7.237945229	7.988895519	9.79E-13	1.55E-10	up
FMOD	1.14000162	8.37850424	7.980310693	1.02E-12	1.61E-10	up
SHANK3	-0.827516703	6.755933366	-7.971485683	1.07E-12	1.68E-10	down
KLF13	-0.68991474	7.725570882	-7.953918673	1.18E-12	1.83E-10	down
CDON	-0.636532266	8.017802218	-7.950410514	1.20E-12	1.85E-10	down
B3GNT6	1.731601136	6.061106912	7.945196631	1.23E-12	1.89E-10	up
ZNF608	-0.679688598	8.895883713	-7.907109531	1.51E-12	2.28E-10	down
EFEMP1	-0.655068617	11.51078859	-7.886442	1.68E-12	2.52E-10	down
AFAP1L1	-0.870417847	7.500957659	-7.862971371	1.90E-12	2.84E-10	down
TMEM178A	-0.989881839	11.00407322	-7.853818847	1.99E-12	2.94E-10	down
MUC5AC	1.85408911	11.65494791	7.849149059	2.04E-12	2.95E-10	up
GMDS	0.747565818	8.922921549	7.848960193	2.04E-12	2.95E-10	up
DNER	1.08916882	10.4342341	7.848035679	2.05E-12	2.95E-10	up
MUC12	1.665125535	5.734145603	7.847919292	2.06E-12	2.95E-10	up
EPAS1	-0.886835348	12.22676794	-7.822246497	2.35E-12	3.34E-10	down
USP13	-0.651957196	8.173627699	-7.820944943	2.37E-12	3.34E-10	down
TCF7L1	-0.77492337	8.891156618	-7.814497021	2.45E-12	3.43E-10	down
THBS3	-0.816073633	8.171732813	-7.808506626	2.53E-12	3.52E-10	down
TPM2	-1.030765926	6.676169649	-7.779714458	2.94E-12	4.07E-10	down
SAA1	-2.3188723	9.379892988	-7.769896459	3.09E-12	4.23E-10	down
SAA2	-2.3188723	9.379892988	-7.769896459	3.09E-12	4.23E-10	down
CD109	0.982634926	7.037510479	7.766616067	3.15E-12	4.25E-10	up
AGR2	0.543525441	14.04297141	7.765832466	3.16E-12	4.25E-10	up
ATP13A4	-0.783396983	7.672731131	-7.737962806	3.66E-12	4.88E-10	down
LHX6	1.036187066	6.469582034	7.715427033	4.12E-12	5.46E-10	up
HMG20A	-0.53358239	8.931923163	-7.71131737	4.20E-12	5.55E-10	down
ABL2	0.50484827	8.158578527	7.692734016	4.63E-12	6.05E-10	up
MT1M	-2.009074274	5.76663515	-7.683383673	4.86E-12	6.31E-10	down
PLPP3	-0.95234219	9.830765843	-7.677611364	5.01E-12	6.43E-10	down
CDK5RAP2	0.573329704	8.743896959	7.665464139	5.34E-12	6.79E-10	up
RNFT2	0.982756096	7.594093255	7.664991179	5.35E-12	6.79E-10	up
NXN	-0.834358638	10.8462731	-7.661523846	5.45E-12	6.84E-10	down
PER2	-0.602389103	10.77349283	-7.657266439	5.57E-12	6.96E-10	down
FBN1	-0.920252034	6.633711791	-7.633638745	6.30E-12	7.78E-10	down
NOL3	0.649421628	8.320181937	7.630194705	6.42E-12	7.88E-10	up
C17orf62	0.615183948	8.896114035	7.628603411	6.47E-12	7.90E-10	up
ZDHHC9	0.579282416	8.631067705	7.616782274	6.88E-12	8.36E-10	up
PRUNE2	0.830241815	10.14362936	7.603247208	7.38E-12	8.88E-10	up
LINC00643	1.114558624	5.960409075	7.596999936	7.63E-12	9.12E-10	up
PIP5K1B	-0.662109638	9.188529331	-7.592510426	7.81E-12	9.29E-10	down
FCER1A	0.907739526	7.641426468	7.587620811	8.01E-12	9.48E-10	up

DLL1	-0.941096665	9.311118899	-7.585850016	8.08E-12	9.52E-10	down
SEMA5A	-0.990167654	8.97106995	-7.584504922	8.14E-12	9.53E-10	down
CDKN2A	0.687213109	7.061950591	7.574252274	8.59E-12	1.00E-09	up
FOCAD	0.510626549	10.19804908	7.563124155	9.10E-12	1.05E-09	up
SCD5	-0.636938456	8.80336519	-7.543425615	1.01E-11	1.16E-09	down
OSBPL6	-0.723618494	11.40922655	-7.533546198	1.06E-11	1.21E-09	down
IL4R	-0.66231127	9.573963571	-7.525001177	1.11E-11	1.26E-09	down
RCOR3	-0.502045098	9.518481374	-7.510145107	1.20E-11	1.35E-09	down
DLGAP1-AS1	0.554985971	8.412237724	7.470978259	1.47E-11	1.64E-09	up
SLC7A2	-0.655622735	10.58207359	-7.468313742	1.49E-11	1.66E-09	down
IGSF6	1.310990825	6.30981892	7.456969751	1.58E-11	1.75E-09	up
BCL2	-0.535289389	7.09757432	-7.455269503	1.59E-11	1.75E-09	down
CKAP4	0.672709308	9.708571836	7.414627426	1.96E-11	2.15E-09	up
ST6GALNAC1	0.575246837	12.66047306	7.41266973	1.98E-11	2.17E-09	up
SULF1	-0.966494881	8.042461286	-7.39376951	2.18E-11	2.38E-09	down
ADGRA3	-0.624343493	8.606871816	-7.382528633	2.31E-11	2.51E-09	down
NPAS3	-0.768253446	6.976170306	-7.377434409	2.38E-11	2.55E-09	down
TMCO4	-0.626146831	8.766378716	-7.377075419	2.38E-11	2.55E-09	down
APELA	-2.33427532	5.735061147	-7.373494759	2.43E-11	2.58E-09	down
C4orf48	0.826970795	9.422664578	7.369602247	2.47E-11	2.62E-09	up
ABCA13	-0.829417483	11.36567426	-7.364611966	2.54E-11	2.67E-09	down
TRIM16	0.856896834	10.0667781	7.360933763	2.59E-11	2.71E-09	up
CYP2F1	-1.040775375	11.61200942	-7.340963467	2.87E-11	2.99E-09	down
FAM46C	-0.642722447	10.4019637	-7.325486883	3.10E-11	3.22E-09	down
MFSD2A	0.86579683	7.726014161	7.322830967	3.15E-11	3.25E-09	up
CYP4F11	1.509396274	7.585747563	7.315341495	3.27E-11	3.36E-09	up
PIEZO2	-0.977321126	6.851059225	-7.293840122	3.65E-11	3.74E-09	down
MET	-0.586432861	10.14482545	-7.292620457	3.67E-11	3.75E-09	down
MLKL	0.949659819	7.869161164	7.281846593	3.88E-11	3.94E-09	up
KLHDC8A	-1.448249431	6.927328359	-7.262881388	4.28E-11	4.32E-09	down
HS3ST3A1	2.068587038	4.565608018	7.24568192	4.67E-11	4.70E-09	up
MT1E	-0.773196552	11.70312241	-7.239289073	4.83E-11	4.84E-09	down
KDELR3	0.829243312	9.095358943	7.222239272	5.27E-11	5.21E-09	up
USP51	-0.623073285	9.368068124	-7.220268234	5.32E-11	5.22E-09	down
LOC101926951	-0.903631532	8.536673816	-7.219448806	5.34E-11	5.22E-09	down
PCDH20	-0.903631532	8.536673816	-7.219448806	5.34E-11	5.22E-09	down
DNAJC12	1.46303478	7.77321059	7.215579659	5.45E-11	5.28E-09	up
TKT	0.873191698	10.52205779	7.213188947	5.52E-11	5.32E-09	up
FAH	0.537992436	7.339955687	7.202228433	5.84E-11	5.57E-09	up
ZNF467	0.877885604	6.791883076	7.201732807	5.85E-11	5.57E-09	up
LINC00942	1.48350681	5.150489574	7.179226167	6.56E-11	6.22E-09	up
KCNH2	0.692031415	7.10343564	7.178281776	6.60E-11	6.22E-09	up
ITGA2	-0.668210076	11.24883065	-7.171498671	6.83E-11	6.42E-09	down
MT1X	-0.974338976	11.50096645	-7.167550374	6.97E-11	6.52E-09	down
MUC5B	-1.319556473	11.02337446	-7.15539074	7.41E-11	6.91E-09	down
MREG	0.677928584	7.876051778	7.152134759	7.54E-11	7.00E-09	up
AKAP12	0.79915376	6.03109082	7.143101056	7.89E-11	7.27E-09	up
PRKAR2B	-0.912517594	9.183420171	-7.140847758	7.98E-11	7.32E-09	down
MIR6778	-0.705770608	8.777901483	-7.133923497	8.27E-11	7.53E-09	down
TFF1	1.621686648	9.281045623	7.133833801	8.27E-11	7.53E-09	up
DUOX2	1.780069064	7.142377986	7.122657345	8.76E-11	7.93E-09	up
BAGE4	-1.059568073	7.479996458	-7.097038222	9.97E-11	8.97E-09	down
CALCA	1.160170527	5.246441274	7.095770455	1.00E-10	8.99E-09	up
ANOS1	-0.851148893	10.34377108	-7.085609217	1.06E-10	9.39E-09	down
ZBTB44	-0.517188199	10.29397378	-7.083163948	1.07E-10	9.44E-09	down
NAV3	-1.20262196	6.235641602	-7.078360716	1.10E-10	9.63E-09	down
CDC14B	0.505870881	9.21171516	7.06728741	1.16E-10	1.01E-08	up
LINC00265	0.939065668	6.259188008	7.053509555	1.24E-10	1.08E-08	up
CAPN13	-0.725060254	8.957047439	-7.043408118	1.31E-10	1.13E-08	down
LOC102724094	1.955023063	7.334842756	7.042444181	1.32E-10	1.13E-08	up
CBX7	-0.544448132	9.863694713	-7.039294372	1.34E-10	1.15E-08	down

TMEM150C	-0.719219985	9.524644412	-7.00761234	1.57E-10	1.34E-08	down
FGFR2	-0.607615202	8.762695408	-6.998219066	1.65E-10	1.39E-08	down
OBSCN	-0.771576604	6.217941191	-6.9953271	1.67E-10	1.41E-08	down
JAKMIP3	1.977135745	5.410943321	6.994618758	1.68E-10	1.41E-08	up
CCDC117	-0.618888481	8.96680993	-6.99302152	1.69E-10	1.41E-08	down
DPYSL3	0.980866908	7.922162647	6.986113535	1.75E-10	1.45E-08	up
DNAH5	-0.647853565	12.56324217	-6.985441071	1.76E-10	1.45E-08	down
CYP4X1	-0.852863182	11.8903792	-6.973379971	1.87E-10	1.54E-08	down
CROT	0.591052214	9.677206553	6.966813201	1.93E-10	1.58E-08	up
RHOU	-0.583457296	10.60471836	-6.934360886	2.27E-10	1.84E-08	down
FAM107A	-0.99830765	8.962228221	-6.932780507	2.29E-10	1.85E-08	down
PTPN3	-0.529406159	10.18933229	-6.926030281	2.37E-10	1.90E-08	down
FXYD2	0.722424089	6.224441882	6.92546421	2.37E-10	1.90E-08	up
CLDN1	-0.698868249	10.83629699	-6.922648357	2.41E-10	1.92E-08	down
GRM5	-1.115399133	6.585801104	-6.918225254	2.46E-10	1.96E-08	down
LOC728196	-0.638112383	11.26754372	-6.910790712	2.56E-10	2.03E-08	down
SRPX2	1.464821831	7.444322016	6.907236781	2.60E-10	2.06E-08	up
ANGPT1	-0.901344974	6.503274017	-6.901427744	2.68E-10	2.11E-08	down
HNRNPF	-0.558968056	11.35835895	-6.900474566	2.69E-10	2.11E-08	down
HEG1	-0.608688513	8.245048799	-6.887687175	2.87E-10	2.25E-08	down
GCOM1	-0.501250485	8.758375735	-6.88444577	2.92E-10	2.27E-08	down
POLR2M	-0.501250485	8.758375735	-6.88444577	2.92E-10	2.27E-08	down
BPIFB2	1.909995038	6.106833359	6.881675924	2.96E-10	2.29E-08	up
NEURL1B	-0.668450757	10.14334995	-6.880892843	2.97E-10	2.29E-08	down
UGT1A1	1.343261648	8.891592918	6.863936186	3.23E-10	2.49E-08	up
SYTL5	0.676740962	8.909292343	6.861113098	3.28E-10	2.51E-08	up
HOTS	2.197037053	5.299161074	6.85527996	3.38E-10	2.58E-08	up
VCAN	0.948146323	8.448047029	6.854677069	3.39E-10	2.58E-08	up
SMARCA1	-0.671291356	8.503404306	-6.839584484	3.65E-10	2.76E-08	down
WFDC1	1.406191759	6.79872741	6.836241513	3.72E-10	2.80E-08	up
CA8	-0.804139867	6.584232195	-6.834980232	3.74E-10	2.81E-08	down
GPT2	0.818697727	7.651002866	6.82001682	4.03E-10	3.02E-08	up
NR0B1	1.252178628	5.444778401	6.81039029	4.23E-10	3.15E-08	up
PTGER4	-0.664400261	11.05783664	-6.810146291	4.23E-10	3.15E-08	down
MT1H	-0.732332927	10.53095606	-6.805828832	4.33E-10	3.21E-08	down
HIST1H2BK	0.655687239	11.28832308	6.802009523	4.41E-10	3.26E-08	up
CD86	1.131755943	6.884075305	6.801003492	4.43E-10	3.27E-08	up
RHOBTB3	-0.667320953	9.201219061	-6.788439438	4.72E-10	3.47E-08	down
UGT1A6	1.342402714	9.306541459	6.781958833	4.88E-10	3.53E-08	up
STEAP2	-0.776904073	9.761305765	-6.777193733	4.99E-10	3.61E-08	down
UGT1A8	1.342237352	9.070787694	6.767668058	5.24E-10	3.76E-08	up
UGT1A9	1.342237352	9.070787694	6.767668058	5.24E-10	3.76E-08	up
ITLN1	-2.384239143	8.043501102	-6.765355039	5.30E-10	3.79E-08	down
FGFR3	-1.293023552	10.12022197	-6.763063414	5.36E-10	3.82E-08	down
LOC115110	-0.558543101	7.957999983	-6.762422445	5.37E-10	3.82E-08	down
TXN	0.600605689	12.59825016	6.751579718	5.67E-10	4.02E-08	up
KCNA1	-1.338259972	7.659628109	-6.74842489	5.76E-10	4.07E-08	down
ALAS1	0.534792947	10.2061762	6.74088398	5.98E-10	4.22E-08	up
PDE7A	-0.541063442	8.23800383	-6.735643162	6.14E-10	4.32E-08	down
FLRT3	-1.012632018	10.02241899	-6.732507377	6.24E-10	4.37E-08	down
UGT1A10	1.351207764	9.522748018	6.720020561	6.64E-10	4.62E-08	up
UGT1A4	1.351207764	9.522748018	6.720020561	6.64E-10	4.62E-08	up
TIMP1	0.811245156	11.6424422	6.690863868	7.67E-10	5.31E-08	up
HLF	-1.214057888	8.455571486	-6.681489718	8.04E-10	5.55E-08	down
TFPI2	-1.362289306	6.768267963	-6.677435667	8.20E-10	5.64E-08	down
ALDH1A3	0.707085221	10.19890081	6.673028978	8.38E-10	5.74E-08	up
BCL2L14	0.62812941	6.654252702	6.668834333	8.56E-10	5.82E-08	up
BAGE2	-0.960664587	6.778747904	-6.660877134	8.90E-10	6.01E-08	down
CPE	1.273446244	7.131756055	6.655066325	9.16E-10	6.15E-08	up
CST6	1.204250082	8.460992904	6.644640922	9.65E-10	6.44E-08	up
SLIT2	-1.05565339	9.631277954	-6.638503853	9.95E-10	6.62E-08	down

MAP1A	0.708297246	9.777534279	6.627985058	1.05E-09	6.95E-08	up
IRX5	-0.663278722	10.419655	-6.618904069	1.10E-09	7.25E-08	down
APCDD1	-0.623225119	9.991354552	-6.613087783	1.13E-09	7.44E-08	down
CRLF1	1.035113695	8.291225604	6.611666879	1.14E-09	7.46E-08	up
SHROOM1	0.794545174	6.519242576	6.611361791	1.14E-09	7.46E-08	up
LMO4	-0.860253008	9.32389431	-6.606864856	1.16E-09	7.59E-08	down
FAR2P2	-1.086451387	8.171137657	-6.591521152	1.25E-09	8.11E-08	down
FAR2P3	-1.086451387	8.171137657	-6.591521152	1.25E-09	8.11E-08	down
SH3BGRL	-0.502833422	12.35639463	-6.584321125	1.30E-09	8.38E-08	down
HIBADH	-0.556936704	10.11215288	-6.581108514	1.32E-09	8.49E-08	down
LAMB3	-0.806573873	10.48042654	-6.570459334	1.39E-09	8.89E-08	down
SYTL4	0.779628795	7.665180921	6.563984212	1.44E-09	9.11E-08	up
LTF	-2.007083708	10.46988388	-6.55026613	1.54E-09	9.64E-08	down
SEC24D	0.62102464	7.936326544	6.539344323	1.62E-09	1.01E-07	up
C10orf107	0.78828311	9.802369982	6.536513121	1.64E-09	1.02E-07	up
RTN4RL1	-1.100483898	7.237863774	-6.515249016	1.83E-09	1.13E-07	down
CDC42EP5	0.990672617	10.74052083	6.511623517	1.86E-09	1.15E-07	up
FICD	0.549734298	9.07760439	6.498735394	1.98E-09	1.22E-07	up
LRRC10B	0.523098486	13.20921434	6.493860544	2.03E-09	1.24E-07	up
LINC00675	0.786972395	7.991461541	6.489847475	2.07E-09	1.26E-07	up
PP14571	1.024377453	7.53504105	6.484607873	2.12E-09	1.29E-07	up
GNAI1	-0.658835154	9.771795699	-6.479668257	2.17E-09	1.31E-07	down
ARHGAP29	-0.570637991	7.670461301	-6.475786625	2.22E-09	1.33E-07	down
ATP6V0A4	1.039176969	8.917395991	6.47553102	2.22E-09	1.33E-07	up
GULP1	0.518823936	10.45547106	6.47091398	2.27E-09	1.35E-07	up
KCNK6	0.694729617	7.37281878	6.47038933	2.27E-09	1.35E-07	up
MT1HL1	-0.837339452	11.58191313	-6.469127344	2.29E-09	1.35E-07	down
SAA2-SAA4	-1.601431123	8.792013931	-6.461724241	2.37E-09	1.40E-07	down
ASPHD2	0.678457612	6.889151782	6.446785778	2.55E-09	1.50E-07	up
LYPLA1	0.55678788	11.25578044	6.442847709	2.60E-09	1.53E-07	up
ATP12A	-1.18427863	10.42332418	-6.436653095	2.68E-09	1.57E-07	down
UGT1A3	1.325675301	8.715471549	6.423416922	2.86E-09	1.66E-07	up
UGT1A5	1.325675301	8.715471549	6.423416922	2.86E-09	1.66E-07	up
ITGB2-AS1	-1.050300859	6.703171382	-6.42104204	2.89E-09	1.67E-07	down
FOXN1	-0.994229111	6.093390409	-6.416927167	2.95E-09	1.70E-07	down
4-Mar	1.5401074	5.927633417	6.404027664	3.14E-09	1.81E-07	up
TOPORS	-0.651044309	9.307184758	-6.397542376	3.25E-09	1.86E-07	down
SPAG9	0.509487427	8.899114736	6.396872687	3.26E-09	1.86E-07	up
TNKS2	-0.648451325	9.533123118	-6.393510633	3.31E-09	1.89E-07	down
MEGF6	-1.239575989	6.350808642	-6.391686455	3.34E-09	1.90E-07	down
TFF3	1.119136216	13.14108199	6.391540097	3.34E-09	1.90E-07	up
CDR2	0.606098987	9.058361065	6.389068615	3.38E-09	1.91E-07	up
LOC101060399	0.606098987	9.058361065	6.389068615	3.38E-09	1.91E-07	up
ANXA3	0.868127816	10.165606	6.383485187	3.48E-09	1.96E-07	up
GALE	0.591559538	8.63487086	6.381345017	3.51E-09	1.97E-07	up
UGT1A7	1.349678923	9.393785646	6.377752053	3.57E-09	2.00E-07	up
SLC6A6	0.577473232	9.87841666	6.374273467	3.63E-09	2.03E-07	up
TRIB2	-0.704836478	9.246715583	-6.369035601	3.73E-09	2.08E-07	down
ADCY2	-0.649025332	8.753001987	-6.331164132	4.48E-09	2.47E-07	down
ZNF540	-0.892478349	6.647747219	-6.331095677	4.48E-09	2.47E-07	down
ADAM28	-0.684934537	9.559487441	-6.328479959	4.54E-09	2.50E-07	down
PCDH17	-0.972568158	7.964303222	-6.325347226	4.61E-09	2.53E-07	down
PAPLN	-0.822343502	6.640450733	-6.313999272	4.87E-09	2.65E-07	down
CYP4F3	1.296396	7.279744687	6.305142095	5.08E-09	2.75E-07	up
FTL	0.562681203	14.61855117	6.301431181	5.17E-09	2.79E-07	up
PSIP1	-0.529140696	10.3993798	-6.298556875	5.25E-09	2.83E-07	down
HN1	0.596580403	9.490270298	6.281510406	5.70E-09	3.05E-07	up
SHMT1	-0.548058661	7.96711913	-6.280629473	5.72E-09	3.05E-07	down
SLC30A1	-0.510318006	9.773387744	-6.265192312	6.16E-09	3.28E-07	down
RHOV	-0.661624107	8.938649343	-6.259398394	6.34E-09	3.37E-07	down
GPR34	1.010224851	7.195984658	6.227341615	7.40E-09	3.89E-07	up

GCLC	0.526379248	11.72484935	6.21505619	7.85E-09	4.10E-07	up
TCF4-AS1	1.436192294	6.472916545	6.214030703	7.89E-09	4.11E-07	up
PNRC2	-0.518019341	11.34883873	-6.210913222	8.01E-09	4.15E-07	down
DENND1C	0.699317935	7.516074003	6.209720967	8.05E-09	4.16E-07	up
TEF	-0.586286333	7.593985975	-6.209567261	8.06E-09	4.16E-07	down
FCGR2B	1.620536756	6.179826605	6.186688831	8.99E-09	4.64E-07	up
FOXO3B	-0.645629393	8.94885674	-6.179145347	9.32E-09	4.78E-07	down
PTGFR	-1.01748807	10.08064336	-6.177269365	9.41E-09	4.82E-07	down
NEURL3	-0.805225353	9.414327162	-6.172093144	9.64E-09	4.92E-07	down
CLEC10A	0.841778856	6.942110212	6.171743234	9.66E-09	4.92E-07	up
ZXDA	-0.522921196	7.002963659	-6.164709113	9.99E-09	5.08E-07	down
TMEM74B	1.058933326	7.648252218	6.16037066	1.02E-08	5.18E-07	up
LOC100288911	-0.664761479	6.554112147	-6.15748643	1.03E-08	5.24E-07	down
MAGI2	-0.573963307	8.630127266	-6.156999718	1.04E-08	5.24E-07	down
THBS1	0.739800991	5.472677374	6.150204085	1.07E-08	5.39E-07	up
SLC16A4	1.154006834	5.990953249	6.148608252	1.08E-08	5.42E-07	up
LOC338667	-1.243801504	6.873973363	-6.144561237	1.10E-08	5.50E-07	down
TMED3	0.528867726	8.418392081	6.140365544	1.12E-08	5.58E-07	up
TIGAR	0.583655634	8.673276481	6.138146223	1.13E-08	5.63E-07	up
KLHL13	0.621460181	10.08517873	6.121337807	1.23E-08	6.07E-07	up
KATNB1	0.539905657	9.925105539	6.11254591	1.28E-08	6.30E-07	up
CTDSPL2	-0.512821224	8.359402928	-6.110967004	1.29E-08	6.33E-07	down
TSC22D3	-0.544926532	9.536649178	-6.109597647	1.30E-08	6.36E-07	down
MRC1	1.721511649	9.165444026	6.108023678	1.31E-08	6.38E-07	up
PEG10	-0.844090581	7.572292411	-6.106635861	1.32E-08	6.41E-07	down
EME1	0.786688048	7.602769035	6.105099783	1.33E-08	6.44E-07	up
HS6ST2	0.97829009	8.492152019	6.081644706	1.49E-08	7.15E-07	up
RRN3P1	-0.657198505	7.803763004	-6.048899278	1.74E-08	8.31E-07	down
GABARAPL3	0.684008127	9.221015801	6.023596264	1.96E-08	9.31E-07	up
FZD8	-0.598873288	8.061496713	-6.019013264	2.00E-08	9.47E-07	down
MIR4683	-0.598873288	8.061496713	-6.019013264	2.00E-08	9.47E-07	down
KLHL6	0.637215528	10.32717583	6.011842093	2.07E-08	9.72E-07	up
RARG	-0.670008311	7.167638029	-6.008588424	2.10E-08	9.81E-07	down
ADAMTS15	-0.719264641	6.322856368	-6.008498847	2.10E-08	9.81E-07	down
HNRNPUL2-BSCL2	0.515815514	10.82376432	5.999076711	2.20E-08	1.02E-06	up
ARHGEF19	-1.036357861	7.457487313	-5.998545233	2.20E-08	1.02E-06	down
ACER2	0.635023452	8.164549467	5.996896246	2.22E-08	1.03E-06	up
SPATS2	0.523992776	7.50553475	5.988325559	2.31E-08	1.07E-06	up
SOCS6	-0.544546717	9.195237382	-5.984407297	2.35E-08	1.08E-06	down
CYP4F2	1.147347567	5.328310834	5.978387735	2.42E-08	1.11E-06	up
RPGRIP1L	0.585039467	9.271567292	5.973992815	2.47E-08	1.13E-06	up
AZGP1	0.949407917	7.762056727	5.969773005	2.52E-08	1.15E-06	up
LOC100505841	0.844188781	8.348347523	5.96967768	2.52E-08	1.15E-06	up
WIPF3	0.877116172	8.260183623	5.966526198	2.56E-08	1.17E-06	up
DNAJC22	0.564796843	7.440212556	5.961237309	2.63E-08	1.19E-06	up
SLCO4C1	-0.742410955	8.493930703	-5.959584758	2.65E-08	1.20E-06	down
DLGAP1	0.619521669	5.725505533	5.958325269	2.66E-08	1.20E-06	up
FKBP1A-SDCBP2	0.644383063	8.93192673	5.958317199	2.66E-08	1.20E-06	up
SDCBP2	0.644383063	8.93192673	5.958317199	2.66E-08	1.20E-06	up
POLA2	-0.551931061	7.731267446	-5.954725414	2.71E-08	1.22E-06	down
ATP6V1B1	-0.822039146	6.813115356	-5.953723336	2.72E-08	1.22E-06	down
MIR6845	-0.616370362	8.893187861	-5.941655243	2.88E-08	1.28E-06	down
NRBP2	-0.616370362	8.893187861	-5.941655243	2.88E-08	1.28E-06	down
GALNT5	0.709067985	7.870559404	5.941302025	2.88E-08	1.28E-06	up
C1orf168	-0.635824442	11.59311826	-5.923413902	3.14E-08	1.39E-06	down
GABPB1	-0.549278287	8.434980225	-5.921448944	3.17E-08	1.39E-06	down
PARVB	0.597975521	6.037865312	5.92125812	3.17E-08	1.39E-06	up
HOOK1	-0.555450081	10.4485496	-5.914781353	3.27E-08	1.43E-06	down
GMPPA	0.506628938	8.347637492	5.912585885	3.30E-08	1.44E-06	up
KCNE1	0.655934171	10.93024519	5.91126192	3.32E-08	1.44E-06	up
TMC7	1.103167624	4.87744923	5.887648299	3.71E-08	1.60E-06	up

TEAD1	-0.502642009	10.54295968	-5.88666963	3.73E-08	1.61E-06	down
MIR4435-2HG	0.627993164	7.20975348	5.881462247	3.82E-08	1.64E-06	up
RAB27B	0.594806275	9.398258725	5.880546526	3.84E-08	1.65E-06	up
HSPA2	-0.892138022	9.701418934	-5.876348142	3.91E-08	1.68E-06	down
BICDL1	0.581680507	7.757911843	5.867082033	4.08E-08	1.74E-06	up
TMCC3	1.132542448	6.152477316	5.86523388	4.12E-08	1.75E-06	up
SNRPF	-0.659295569	10.45724768	-5.862739755	4.17E-08	1.77E-06	down
SLC19A2	-0.535395228	9.935724909	-5.862248425	4.18E-08	1.77E-06	down
ZBTB16	-1.034610914	7.528844557	-5.859971691	4.22E-08	1.78E-06	down
ADD3-AS1	0.657462521	5.988774308	5.859573875	4.23E-08	1.78E-06	up
PGD	0.656108372	9.665691711	5.858755949	4.25E-08	1.79E-06	up
GMNN	-0.564570197	10.12970837	-5.856577354	4.29E-08	1.80E-06	down
DEPTOR	-0.637838741	7.936153717	-5.846480645	4.50E-08	1.88E-06	down
EPHX2	-0.512859679	9.661447902	-5.842113508	4.59E-08	1.92E-06	down
PCSK6	-0.527489511	6.721083137	-5.831207164	4.83E-08	2.00E-06	down
RIMS1	-0.865344018	7.851685723	-5.827886528	4.90E-08	2.03E-06	down
JAG1	-0.540343873	9.737551952	-5.823489306	5.01E-08	2.07E-06	down
KDM3B	-0.595890019	11.03104149	-5.820312848	5.08E-08	2.09E-06	down
BCL2A1	1.423222422	8.758724914	5.812884075	5.26E-08	2.16E-06	up
CYP4Z1	-0.76687288	8.451297125	-5.801111205	5.56E-08	2.27E-06	down
BCAT1	0.99960499	5.94299622	5.800936331	5.56E-08	2.27E-06	up
BACE2	0.582372003	10.32346898	5.794029878	5.74E-08	2.34E-06	up
SNED1	-0.632248556	8.141239103	-5.788724299	5.88E-08	2.39E-06	down
FAM3B	0.970298323	10.08015765	5.787570647	5.92E-08	2.40E-06	up
ABCC1	0.533138516	9.782842826	5.785883095	5.96E-08	2.41E-06	up
SLC7A5	1.187529506	6.484540589	5.780420888	6.12E-08	2.47E-06	up
DAB2	0.889408308	7.329997843	5.768412879	6.47E-08	2.61E-06	up
CHKA	-0.563507741	9.073729992	-5.763502535	6.61E-08	2.65E-06	down
LOC101926921	0.906521004	7.482843687	5.758795584	6.76E-08	2.69E-06	up
EMB	-0.796315352	10.90965979	-5.75582912	6.85E-08	2.72E-06	down
MALL	-0.756994632	9.606391886	-5.743178513	7.27E-08	2.87E-06	down
SPDEF	0.824180823	9.04474334	5.739485331	7.39E-08	2.91E-06	up
PFKFB3	0.579107988	9.762392478	5.737598954	7.46E-08	2.93E-06	up
FCGBP	-1.423989288	11.00002944	-5.73596852	7.51E-08	2.94E-06	down
SLC39A10	-0.600876132	9.427238354	-5.731120549	7.68E-08	2.99E-06	down
SPRY2	-0.613909016	9.785940603	-5.725636645	7.88E-08	3.07E-06	down
ALDH7A1	-0.502767857	9.566792893	-5.723229874	7.97E-08	3.09E-06	down
PDE7B	-0.513731689	7.307306036	-5.7229616	7.98E-08	3.09E-06	down
SERPINB3	-1.149283855	13.55446178	-5.719861013	8.09E-08	3.13E-06	down
TRAF5	-0.62143246	7.518097343	-5.711232906	8.42E-08	3.23E-06	down
MT2A	-0.597978823	12.21876151	-5.710178759	8.46E-08	3.24E-06	down
ZNF703	-0.633424165	8.809561605	-5.688446413	9.35E-08	3.54E-06	down
SH3D19	0.525090749	9.777213463	5.68685044	9.42E-08	3.56E-06	up
FBXL7	0.67741163	8.370220816	5.68362986	9.56E-08	3.59E-06	up
GALNT7	0.530629431	11.01065834	5.683526954	9.57E-08	3.59E-06	up
FXYD6-FXYD2	1.019820194	6.206379267	5.682174247	9.63E-08	3.60E-06	up
CGREF1	1.24347767	4.574276313	5.682131489	9.63E-08	3.60E-06	up
QPCT	0.788271345	6.92105591	5.68128826	9.67E-08	3.61E-06	up
LOC283177	-1.112587729	7.541612721	-5.677239398	9.85E-08	3.66E-06	down
ENC1	-0.569047095	9.30130826	-5.675902046	9.91E-08	3.68E-06	down
NTN1	-0.538811561	8.498252931	-5.671330733	1.01E-07	3.75E-06	down
MT1G	-0.669985949	9.243341421	-5.667317042	1.03E-07	3.81E-06	down
SLC39A8	0.612338415	7.39834485	5.659637845	1.07E-07	3.93E-06	up
AZU1	-0.862117632	7.323967748	-5.657928989	1.08E-07	3.94E-06	down
GPX3	0.7685672	10.92013298	5.651231497	1.11E-07	4.06E-06	up
FGFBP1	0.998935827	8.69443495	5.64470158	1.14E-07	4.16E-06	up
SETP4	-1.047119361	10.11748986	-5.641297007	1.16E-07	4.22E-06	down
INHBC	0.704012843	8.016550165	5.640639973	1.16E-07	4.22E-06	up
EVC	-0.692398745	6.218488571	-5.636623166	1.19E-07	4.27E-06	down
LAMP3	0.626040937	10.30925196	5.634022695	1.20E-07	4.32E-06	up
PIP5K1A	-0.524654459	7.917729154	-5.630896774	1.22E-07	4.37E-06	down

IL1B	1.067965115	8.274446271	5.62407174	1.26E-07	4.50E-06	up
SIX3-AS1	1.289041173	5.170506174	5.623321862	1.26E-07	4.51E-06	up
FHAD1	0.510802428	9.64704224	5.610318616	1.34E-07	4.77E-06	up
HEPACAM2	0.794518728	9.220405592	5.607439154	1.36E-07	4.82E-06	up
CPNE4	1.162638968	4.906662918	5.604859855	1.37E-07	4.86E-06	up
MYADM	0.56124536	10.57492889	5.6024348	1.39E-07	4.91E-06	up
PKIB	-0.737125614	10.66152805	-5.602013392	1.39E-07	4.91E-06	down
DUSP5P1	1.489425214	5.359615926	5.596549354	1.42E-07	5.03E-06	up
SCGB2A1	0.607835747	11.81219314	5.593939481	1.44E-07	5.08E-06	up
LINC00152	0.600240403	7.849997074	5.590250428	1.47E-07	5.11E-06	up
LOC101930489	0.600240403	7.849997074	5.590250428	1.47E-07	5.11E-06	up
PNLDC1	0.596095514	8.607898593	5.586301964	1.49E-07	5.20E-06	up
SLC51A	0.739934889	9.337660575	5.583878345	1.51E-07	5.23E-06	up
IL33	-1.043572203	10.24619126	-5.583696087	1.51E-07	5.23E-06	down
PLA2G4A	0.839875558	8.095460644	5.583613197	1.51E-07	5.23E-06	up
CYSRT1	0.938986368	7.353280254	5.582889182	1.52E-07	5.24E-06	up
CDH11	-1.014665902	6.237210225	-5.582495888	1.52E-07	5.24E-06	down
GPRASP1	-0.592536041	8.433983755	-5.576821151	1.56E-07	5.37E-06	down
FMO3	-0.928236389	10.31582959	-5.575561077	1.57E-07	5.39E-06	down
CFAP161	0.621169363	9.853841032	5.571454003	1.60E-07	5.48E-06	up
ALOX5	1.083063257	8.470574586	5.571246988	1.60E-07	5.48E-06	up
LINC01590	0.876185295	7.383186712	5.570810945	1.60E-07	5.49E-06	up
S100P	0.664163667	13.54832991	5.569732023	1.61E-07	5.50E-06	up
VDR	0.537665009	7.399615309	5.566900984	1.63E-07	5.56E-06	up
CLDN8	-0.829880133	10.91095454	-5.550166452	1.76E-07	5.97E-06	down
IZUMO4	-0.592282547	7.111517607	-5.543891226	1.81E-07	6.12E-06	down
PLA2G7	1.606268251	6.322720223	5.542925744	1.82E-07	6.13E-06	up
TMCO5A	0.674963002	6.678141764	5.542890021	1.82E-07	6.13E-06	up
LOC100996583	-0.74751967	7.299177619	-5.542381113	1.82E-07	6.13E-06	down
SLC39A14	0.530121598	6.828685388	5.534758764	1.89E-07	6.33E-06	up
C22orf42	1.042408932	5.618320525	5.527558301	1.95E-07	6.52E-06	up
LOC102724782	0.541196104	7.674816295	5.520679256	2.01E-07	6.71E-06	up
PRR4	1.251858436	7.658647863	5.517400498	2.04E-07	6.79E-06	up
ARHGAP28	-0.830064618	5.023445465	-5.514642912	2.07E-07	6.85E-06	down
MT3	-0.980190236	7.491056561	-5.511858282	2.09E-07	6.92E-06	down
GATSL3	-0.638241877	7.14653164	-5.510086363	2.11E-07	6.96E-06	down
ZBTB1	-0.5076452	9.184763632	-5.506634464	2.14E-07	7.05E-06	down
SESN3	-0.809179998	6.765209917	-5.502174737	2.19E-07	7.19E-06	down
ZNF680	-0.769211029	8.903149365	-5.499090828	2.22E-07	7.26E-06	down
CENPC	-0.50078162	8.907399789	-5.489035519	2.32E-07	7.55E-06	down
MYLK3	0.68981627	6.65495211	5.488302257	2.33E-07	7.55E-06	up
PTHLH	0.851264931	5.185739519	5.488163159	2.33E-07	7.55E-06	up
NANOS1	0.663301226	6.414270875	5.482050609	2.40E-07	7.73E-06	up
LINC01315	-0.533430195	9.907192335	-5.478870282	2.43E-07	7.81E-06	down
SLC2A3	0.94989896	6.925956088	5.478634731	2.43E-07	7.81E-06	up
FBXW10	-0.596860896	9.490337175	-5.477784263	2.44E-07	7.82E-06	down
LOC100996906	-0.596860896	9.490337175	-5.477784263	2.44E-07	7.82E-06	down
PAX1	-0.891015733	6.484400268	-5.477173365	2.45E-07	7.83E-06	down
PCDH7	-0.572917948	9.181334346	-5.474213903	2.48E-07	7.93E-06	down
NR4A3	1.128389148	7.125563779	5.470356752	2.53E-07	8.05E-06	up
MZB1	0.857614652	5.732717553	5.470010583	2.53E-07	8.05E-06	up
SOX5	-0.558806991	7.294707486	-5.466360757	2.57E-07	8.18E-06	down
MIER3	-0.56328471	7.954047906	-5.461423394	2.63E-07	8.34E-06	down
WNT2B	-0.767248761	6.894696501	-5.45320737	2.73E-07	8.61E-06	down
BTG4	-0.520418448	8.267967791	-5.453149601	2.73E-07	8.61E-06	down
F5	0.878199511	6.524125205	5.440904302	2.88E-07	9.03E-06	up
LYNX1	-0.664959309	6.794257521	-5.43757532	2.93E-07	9.13E-06	down
CCND1	-0.538102591	9.870828134	-5.435137309	2.96E-07	9.20E-06	down
C1QTNF9B-AS1	0.877452301	7.161771571	5.433275526	2.98E-07	9.26E-06	up
TRIM23	-0.849943297	7.68838031	-5.429728418	3.03E-07	9.39E-06	down
SIDT2	0.539416062	10.09256901	5.426136086	3.08E-07	9.50E-06	up

MCEMP1	1.854352687	8.479018001	5.426091578	3.08E-07	9.50E-06	up
VEPH1	-0.767131983	5.942296574	-5.425740448	3.09E-07	9.50E-06	down
SELENBP1	-0.516534022	12.66111608	-5.425374372	3.09E-07	9.50E-06	down
RPRM	0.78233306	8.351959992	5.417437525	3.20E-07	9.82E-06	up
CREB3L1	0.799538322	9.65007853	5.410463713	3.30E-07	1.01E-05	up
EPHX3	-0.657936895	7.735516247	-5.397974473	3.49E-07	1.06E-05	down
TCN1	1.671626928	8.958581907	5.388754599	3.64E-07	1.10E-05	up
IL5RA	-0.712464729	8.006639367	-5.380746327	3.77E-07	1.14E-05	down
FAM110C	0.869020347	8.105446654	5.377629476	3.83E-07	1.15E-05	up
GMNC	-1.327745987	6.022998744	-5.37559975	3.86E-07	1.15E-05	down
ATAD2B	-0.534791106	7.459642592	-5.375476121	3.86E-07	1.15E-05	down
EDARADD	-1.12760108	7.56718575	-5.371434517	3.93E-07	1.17E-05	down
CA12	0.822061523	8.152140891	5.369824958	3.96E-07	1.18E-05	up
COL4A5	-0.623361662	9.426763204	-5.367173116	4.01E-07	1.19E-05	down
MIPOL1	0.739013373	7.189093412	5.367046226	4.01E-07	1.19E-05	up
ZNF585B	-0.536220833	7.679983674	-5.366496956	4.02E-07	1.19E-05	down
CD101	0.812918018	6.276522812	5.363162474	4.08E-07	1.20E-05	up
PHTF2	0.509532635	8.170319208	5.343863071	4.45E-07	1.30E-05	up
LINC01558	-0.793371554	7.054777504	-5.323994501	4.86E-07	1.41E-05	down
LOC100507560	-1.313729077	7.057353792	-5.323470293	4.87E-07	1.42E-05	down
TREM2	1.518881684	6.864490739	5.323194973	4.88E-07	1.42E-05	up
IL19	1.136195853	6.214302416	5.322833066	4.89E-07	1.42E-05	up
ST8SIA1	0.713989606	6.909089499	5.321879774	4.91E-07	1.42E-05	up
BCL11A	-0.53802791	9.220039645	-5.315982527	5.04E-07	1.45E-05	down
CYP2A13	-0.875432844	7.632585896	-5.312051472	5.12E-07	1.48E-05	down
GOLGA8B	-0.889596119	9.815120006	-5.307161803	5.24E-07	1.50E-05	down
LOC101930583	-0.889596119	9.815120006	-5.307161803	5.24E-07	1.50E-05	down
ARHGAP33	-0.735418587	6.368922134	-5.307022138	5.24E-07	1.50E-05	down
MOB1B	-0.574149223	9.756495633	-5.304067476	5.31E-07	1.52E-05	down
LOC105376081	-0.906960944	9.032478151	-5.302887388	5.34E-07	1.52E-05	down
ATP8A1	-0.662650857	7.276524234	-5.299803595	5.41E-07	1.54E-05	down
SLAMF7	-0.969044028	7.840816864	-5.290064509	5.65E-07	1.59E-05	down
SNTB1	-0.525238582	8.964869746	-5.289798408	5.66E-07	1.59E-05	down
ZNF663P	-1.014108827	6.54500099	-5.286168557	5.75E-07	1.62E-05	down
EFHD1	-0.657716232	8.713180524	-5.28407775	5.80E-07	1.63E-05	down
GLUD2	-0.617536656	8.6621577	-5.277774275	5.96E-07	1.67E-05	down
DNASE2B	1.478747889	5.110037317	5.277005733	5.98E-07	1.67E-05	up
IL12A	-0.682532913	7.186829909	-5.265139415	6.31E-07	1.76E-05	down
LOC105375172	1.05845946	6.812333496	5.262184124	6.39E-07	1.77E-05	up
GGT7	-0.510662443	7.716825333	-5.258617329	6.49E-07	1.80E-05	down
COL8A1	0.891951118	5.084709942	5.257677416	6.52E-07	1.80E-05	up
DLG1-AS1	0.68069903	6.926414068	5.255526636	6.58E-07	1.81E-05	up
IRX3	-0.732412275	12.80014939	-5.254257033	6.62E-07	1.82E-05	down
GABARAPL1	0.521342952	9.24117736	5.248214706	6.79E-07	1.87E-05	up
ZNF818P	-0.610287202	7.870789375	-5.248158357	6.80E-07	1.87E-05	down
KLF2	0.535593291	9.382027654	5.239040696	7.07E-07	1.93E-05	up
USP21	-0.506403818	10.2788121	-5.235060872	7.20E-07	1.96E-05	down
HSPB8	0.736730666	6.29945188	5.233025928	7.26E-07	1.97E-05	up
SPRR3	1.902933933	6.325437123	5.224222338	7.55E-07	2.04E-05	up
C1QTNF4	-1.265237145	4.529189853	-5.223695156	7.57E-07	2.04E-05	down
SKAP1	-0.85114546	7.919828639	-5.221350238	7.65E-07	2.06E-05	down
STAB1	0.592352468	6.375658812	5.219681253	7.70E-07	2.07E-05	up
ACSL1	0.507441228	10.1995371	5.219564641	7.71E-07	2.07E-05	up
GBP6	0.550975052	9.092832539	5.215793803	7.84E-07	2.09E-05	up
ZNF416	-0.540555309	6.930691233	-5.209155214	8.07E-07	2.14E-05	down
ADM	1.034577808	7.458786783	5.205379013	8.20E-07	2.18E-05	up
LINC01267	-0.583309303	8.46184785	-5.202025231	8.32E-07	2.21E-05	down
BAALC	0.871451983	7.69741477	5.194296893	8.61E-07	2.26E-05	up
GLIS2	-0.69408428	7.653665541	-5.180229246	9.16E-07	2.40E-05	down
AJUBA	-0.634508528	8.231806611	-5.178361297	9.23E-07	2.42E-05	down
ZNF665	-0.573224683	10.22871257	-5.176157947	9.32E-07	2.43E-05	down

GAS6	-0.532481047	9.314992737	-5.176040072	9.33E-07	2.43E-05	down
EFNB2	-0.515496331	9.8196147	-5.174412078	9.39E-07	2.45E-05	down
PRKCDBP	0.54389679	8.796024917	5.174118047	9.40E-07	2.45E-05	up
IMPA2	0.606269358	10.11377222	5.16389037	9.83E-07	2.55E-05	up
GRM1	0.693228681	5.158200026	5.161098272	9.95E-07	2.58E-05	up
DNASE1L3	0.514101582	6.86503854	5.156065265	1.02E-06	2.63E-05	up
ZNF837	-0.636599484	7.389697361	-5.150236213	1.04E-06	2.69E-05	down
TMEM65	-0.55231084	8.197169826	-5.14225469	1.08E-06	2.78E-05	down
C3orf14	0.551222831	8.543169382	5.130939954	1.14E-06	2.90E-05	up
TSPAN5	0.699132966	6.933944025	5.125336661	1.16E-06	2.96E-05	up
SLC51B	0.727769159	8.194073595	5.118581231	1.20E-06	3.05E-05	up
HCG8	-0.612724686	6.924236564	-5.117357753	1.20E-06	3.06E-05	down
GOLGA8A	-0.793754402	10.16321236	-5.116789349	1.21E-06	3.06E-05	down
PRSS12	-0.501229867	10.94453179	-5.11607569	1.21E-06	3.07E-05	down
1-Mar	-0.905453003	7.716786322	-5.111833079	1.23E-06	3.12E-05	down
EDEM3	0.528415001	10.13986578	5.109827911	1.24E-06	3.14E-05	up
CXCL14	1.444904003	5.644039866	5.107357183	1.26E-06	3.16E-05	up
FERMT1	0.517153335	8.117953351	5.104155824	1.28E-06	3.20E-05	up
COL9A2	-0.526057107	7.917217339	-5.090428092	1.35E-06	3.37E-05	down
C6orf99	0.511494492	7.581446515	5.085952379	1.38E-06	3.43E-05	up
RIT1	0.515181294	8.705610259	5.072359634	1.46E-06	3.62E-05	up
H19	1.680117921	8.961738534	5.06982624	1.48E-06	3.65E-05	up
MIR675	1.680117921	8.961738534	5.06982624	1.48E-06	3.65E-05	up
SERPINB4	-1.299828271	12.0415147	-5.063674662	1.52E-06	3.74E-05	down
CACNB4	-0.836794797	5.47493508	-5.054544297	1.58E-06	3.86E-05	down
GXYLT2	-0.729200987	8.867295962	-5.053822747	1.59E-06	3.86E-05	down
LOC100130744	-1.369840286	4.420483143	-5.053301842	1.59E-06	3.86E-05	down
ANKRD20A5P	0.805747988	8.730570287	5.051770439	1.60E-06	3.88E-05	up
SCG3	1.605197444	4.10214928	5.05127666	1.60E-06	3.89E-05	up
HPGDS	0.894886162	7.835307815	5.04463792	1.65E-06	3.97E-05	up
C10orf10	-1.018708289	6.162298487	-5.043949292	1.65E-06	3.98E-05	down
PRH1-PRR4	1.091443643	7.243749133	5.042482173	1.66E-06	4.00E-05	up
ISLR	-0.944472546	7.059821814	-5.042209492	1.67E-06	4.00E-05	down
PIK3AP1	0.683442992	8.303117869	5.041025725	1.67E-06	4.02E-05	up
GSTT2	-1.456332949	6.371129952	-5.038595901	1.69E-06	4.05E-05	down
LCAT	-0.962976017	4.95679847	-5.032048384	1.74E-06	4.15E-05	down
BHLHA15	0.78911975	6.98872948	5.024981993	1.79E-06	4.25E-05	up
FAM193B	-0.503429815	8.387374756	-5.021785409	1.82E-06	4.29E-05	down
ZCCHC4	-0.553510156	6.76570636	-5.018083057	1.85E-06	4.35E-05	down
LOC730101	0.560084877	9.741541226	5.017904489	1.85E-06	4.35E-05	up
ANKRD20A11P	0.765154513	9.707860828	5.012110805	1.90E-06	4.43E-05	up
CPEB1	0.620196704	8.162763435	5.011977283	1.90E-06	4.43E-05	up
ASCL2	-0.802329896	6.49300748	-5.006789207	1.94E-06	4.53E-05	down
TSPAN13	0.530617597	13.7828883	4.99841228	2.01E-06	4.68E-05	up
ANO10	0.509795896	8.283922304	4.992425032	2.06E-06	4.78E-05	up
GPR155	-0.896783879	6.533514109	-4.990805693	2.08E-06	4.81E-05	down
CHPT1	-0.659806946	9.001617828	-4.989130375	2.09E-06	4.84E-05	down
SLC2A14	0.831025942	7.023658612	4.988237624	2.10E-06	4.85E-05	up
CLK4	-0.514414479	9.775573309	-4.986740474	2.11E-06	4.88E-05	down
TRPC1	-0.589943541	7.785405414	-4.986533928	2.12E-06	4.88E-05	down
SLCO3A1	0.619923811	8.14576893	4.983595634	2.14E-06	4.92E-05	up
ANO4	-0.642778418	5.387375371	-4.983539159	2.14E-06	4.92E-05	down
WNT9A	0.567138643	7.652077098	4.975568476	2.22E-06	5.05E-05	up
CYBRD1	-0.804741857	8.670960253	-4.971221761	2.26E-06	5.14E-05	down
WNK4	-1.026261591	6.431354337	-4.970616451	2.27E-06	5.15E-05	down
SLC35F2	0.631353467	6.992801464	4.968196679	2.29E-06	5.19E-05	up
CDH12	-1.097282063	7.164694648	-4.964341773	2.33E-06	5.26E-05	down
ALOX15	-0.670773507	11.80243269	-4.962358811	2.35E-06	5.30E-05	down
GK6P	-0.593596688	8.613756413	-4.961601864	2.35E-06	5.31E-05	down
AGPAT4-IT1	0.584784556	7.344577411	4.960991454	2.36E-06	5.32E-05	up
SAMD5	0.936577965	4.556379098	4.955201253	2.42E-06	5.42E-05	up

LINC01006	-1.044970255	6.066798816	-4.954141963	2.43E-06	5.43E-05	down
ITGAM	0.86012003	7.005849681	4.953928353	2.43E-06	5.43E-05	up
SAA4	-1.212776399	8.699457601	-4.927874727	2.72E-06	5.98E-05	down
POTEM	-0.875091755	6.126493	-4.923243443	2.77E-06	6.08E-05	down
RBMS3	-0.568037863	7.700239175	-4.911411473	2.91E-06	6.36E-05	down
LINC01187	0.690727853	6.066007522	4.906529937	2.97E-06	6.48E-05	up
PLEKHG3	-0.571867391	6.86298068	-4.904710215	3.00E-06	6.51E-05	down
SMIM10L2B	-0.933647246	6.256044883	-4.902877592	3.02E-06	6.55E-05	down
ACADM	-0.551366979	11.86289221	-4.899474048	3.06E-06	6.63E-05	down
PALM	-0.702307605	7.783386155	-4.893814091	3.14E-06	6.77E-05	down
PPM1L	-0.638556812	7.833751893	-4.891780356	3.17E-06	6.80E-05	down
CD163	1.12606097	8.301086134	4.890227007	3.19E-06	6.84E-05	up
EVA1A	-0.883154147	6.736767291	-4.890035575	3.19E-06	6.84E-05	down
ADH6	0.675186377	9.700334781	4.889343321	3.20E-06	6.85E-05	up
LHFPL2	0.580580404	8.271006156	4.888697902	3.21E-06	6.87E-05	up
SERTAD4-AS1	-0.56182742	9.835954987	-4.885102689	3.26E-06	6.96E-05	down
GPR183	0.882516594	7.131018446	4.884881354	3.26E-06	6.96E-05	up
CHAD	1.1765953	8.144700874	4.884007573	3.27E-06	6.98E-05	up
RETN	1.158843742	6.576187335	4.876938164	3.37E-06	7.17E-05	up
LINC01106	-0.513914464	6.132721079	-4.87115021	3.45E-06	7.30E-05	down
LINC01123	-0.513914464	6.132721079	-4.87115021	3.45E-06	7.30E-05	down
PTAFR	0.509592135	8.675007426	4.867958602	3.50E-06	7.37E-05	up
FGFRL1	-0.655672414	6.877434687	-4.866775607	3.52E-06	7.40E-05	down
LPCAT2	0.502831715	6.912412002	4.862478604	3.58E-06	7.51E-05	up
FN1	1.439774534	9.691231357	4.855907306	3.68E-06	7.70E-05	up
ANKRD20A9P	0.839751996	10.13703123	4.855499378	3.69E-06	7.70E-05	up
LOC102723891	0.839751996	10.13703123	4.855499378	3.69E-06	7.70E-05	up
LOC102725051	0.839751996	10.13703123	4.855499378	3.69E-06	7.70E-05	up
KRT40	1.090005409	5.070938994	4.8547858	3.70E-06	7.71E-05	up
VGLL3	-1.079382912	6.759817042	-4.850858817	3.76E-06	7.81E-05	down
DEGS2	-0.691032751	10.23556668	-4.839791545	3.94E-06	8.11E-05	down
APOBEC3B	0.839534796	7.040460417	4.836605938	3.99E-06	8.20E-05	up
PKP1	-1.391069028	6.156888934	-4.836555789	4.00E-06	8.20E-05	down
COL5A2	-0.506311143	7.328826497	-4.831262745	4.09E-06	8.37E-05	down
TGFBI	0.678790178	9.852447106	4.822841651	4.23E-06	8.65E-05	up
ST8SIA5	0.741222791	6.036587839	4.821891992	4.25E-06	8.68E-05	up
ANKRD22	0.601899914	6.891060051	4.819816787	4.29E-06	8.74E-05	up
CCR1	0.901156312	7.653186541	4.817776985	4.32E-06	8.81E-05	up
ANKRD20A1	0.798675048	8.670479256	4.810875907	4.45E-06	9.01E-05	up
ANKRD20A2	0.798675048	8.670479256	4.810875907	4.45E-06	9.01E-05	up
ANKRD20A3	0.798675048	8.670479256	4.810875907	4.45E-06	9.01E-05	up
ANKRD20A4	0.798675048	8.670479256	4.810875907	4.45E-06	9.01E-05	up
ALOX5AP	1.055984015	10.74443696	4.808349248	4.50E-06	9.08E-05	up
BPIFA2	1.233533776	5.797932076	4.807619181	4.51E-06	9.09E-05	up
TRPV2	0.810757818	6.68597992	4.805055078	4.56E-06	9.18E-05	up
OSM	0.960154028	5.355825858	4.801834518	4.62E-06	9.25E-05	up
CYBB	0.805919193	7.06155189	4.801585554	4.63E-06	9.25E-05	up
MINOS1-NBL1	0.572157198	10.65563687	4.800983572	4.64E-06	9.27E-05	up
LOC100653086	0.916680736	7.420631429	4.793943744	4.78E-06	9.52E-05	up
ZNF853	-0.555969198	7.538588439	-4.790662364	4.84E-06	9.63E-05	down
CPA3	1.52581457	10.24173727	4.790431922	4.85E-06	9.63E-05	up
ADGRE2	0.730362624	6.398337747	4.789412922	4.87E-06	9.67E-05	up
LOC100507472	-0.531413707	7.491292321	-4.786137465	4.93E-06	9.77E-05	down
FGR	0.934579598	8.287210888	4.784300697	4.97E-06	9.83E-05	up
FCN1	0.974132727	6.135174834	4.779122155	5.08E-06	9.99E-05	up
DACT2	-0.991995799	6.041792277	-4.772888877	5.22E-06	0.000102315	down
RAP1GAP	0.585024181	8.14639426	4.772236784	5.23E-06	0.000102506	up
SRD5A2	0.541212162	10.19736408	4.769817197	5.28E-06	0.000103455	up
NECTIN3	-0.832225224	6.122576154	-4.768164435	5.32E-06	0.000103991	down
FRAS1	-0.547536378	6.22614521	-4.764907626	5.39E-06	0.000105141	down
LINC00930	0.874356096	7.451801169	4.758891956	5.53E-06	0.00010753	up

PARM1	0.664066947	7.945132905	4.756950188	5.57E-06	0.000108309	up
RBM20	-0.55121503	10.614973	-4.747664835	5.79E-06	0.000112379	down
RTKN	-0.802704466	6.233085841	-4.741134181	5.95E-06	0.000115126	down
MAFG	0.585574541	7.601253867	4.741029755	5.95E-06	0.000115126	up
RASSF10	-0.552936769	9.143127874	-4.728578751	6.27E-06	0.000120511	down
PSMA3-AS1	-0.571434887	9.486285182	-4.725470506	6.35E-06	0.00012197	down
ACP5	0.981033894	9.069022109	4.724466109	6.38E-06	0.000122273	up
CYP2A7	-0.525994482	8.272541435	-4.720479133	6.48E-06	0.000123886	down
SAMSN1	0.915208737	7.354064912	4.718098316	6.55E-06	0.000124805	up
AGPAT4	0.630028146	7.272481454	4.716391563	6.59E-06	0.000125291	up
DSPP	0.785683995	6.162027166	4.714155027	6.66E-06	0.000126264	up
C8orf76	0.515159476	9.274951464	4.706274909	6.88E-06	0.000129616	up
FTO	-0.534003686	12.20133223	-4.70491704	6.91E-06	0.000129851	down
WNT4	-0.752919671	6.779513162	-4.704902254	6.91E-06	0.000129851	down
ST3GAL4	0.601975857	6.329557051	4.690997031	7.32E-06	0.000137068	up
BHMT2	0.673227946	4.781624245	4.690025596	7.35E-06	0.000137382	up
FPR3	0.815891647	7.152708033	4.687166575	7.44E-06	0.000138681	up
CDH2	0.778746198	6.390553315	4.686806916	7.45E-06	0.000138731	up
DHX38	-0.546028521	8.219239137	-4.686682781	7.45E-06	0.000138731	down
BANK1	-0.645387479	6.75577377	-4.685973865	7.48E-06	0.000138868	down
PMEPA1	0.551928264	6.938366026	4.678426058	7.71E-06	0.000142422	up
FCMR	-0.570719107	7.59204803	-4.676072173	7.79E-06	0.000143523	down
ATF6B	-0.623936201	6.4618297	-4.67257151	7.90E-06	0.000145136	down
HDAC5	-0.554030079	8.046174762	-4.671619189	7.93E-06	0.000145472	down
UHRF1	0.668006563	6.750587627	4.669862113	7.99E-06	0.000146291	up
GDF9	-0.508984664	7.636445735	-4.668962881	8.02E-06	0.000146481	down
SLC23A1	-0.691250504	9.700315588	-4.660232715	8.31E-06	0.000150984	down
ALKBH8	-0.523670941	7.270245048	-4.656467885	8.44E-06	0.000153127	down
MIR3945HG	0.731088339	6.071854976	4.656413224	8.44E-06	0.000153127	up
ITM2C	-0.520061763	9.112072304	-4.654681817	8.50E-06	0.000153974	down
FRG1KP	-0.753794854	8.577497276	-4.64308692	8.92E-06	0.000160452	down
LOC100134091	-0.753794854	8.577497276	-4.64308692	8.92E-06	0.000160452	down
TLR8	1.014867489	6.870471253	4.641805785	8.96E-06	0.000161043	up
COL12A1	-0.68444638	5.337366747	-4.638580673	9.08E-06	0.000162799	down
PIK3R1	-0.602388371	9.111633513	-4.6381696	9.10E-06	0.000162946	down
GABRE	-1.091416672	7.606496097	-4.632717978	9.30E-06	0.000165971	down
MIR224	-1.091416672	7.606496097	-4.632717978	9.30E-06	0.000165971	down
MIR452	-1.091416672	7.606496097	-4.632717978	9.30E-06	0.000165971	down
MFSD14C	-0.817912209	8.79674847	-4.620835365	9.77E-06	0.000173114	down
CD81	-0.562106321	13.01189276	-4.620505978	9.78E-06	0.000173114	down
LOC102723721	0.891874576	5.964638812	4.619730649	9.81E-06	0.000173529	up
TNNI3	0.653455496	8.106876802	4.619247819	9.83E-06	0.000173736	up
SCG2	0.886132774	6.623150182	4.617924881	9.88E-06	0.000174503	up
SPRR1B	1.418613131	5.692600886	4.617790624	9.89E-06	0.000174503	up
TMEM47	0.753374626	8.784453975	4.614908085	1.00E-05	0.000175889	up
SLC16A2	-1.023501906	6.376280116	-4.610724588	1.02E-05	0.000178642	down
MYO15B	-0.583449756	8.541561707	-4.608401452	1.03E-05	0.000180204	down
TESPA1	0.631843134	6.101172974	4.603661325	1.05E-05	0.000183438	up
CWH43	1.181373675	8.880642092	4.597897211	1.07E-05	0.000187365	up
CES1P1	-1.534367109	6.978787446	-4.595730605	1.08E-05	0.000188592	down
HRASLS2	0.503909189	8.972777798	4.595028913	1.08E-05	0.000188908	up
CARD6	0.57542466	8.806539598	4.592832888	1.09E-05	0.00018996	up
NR2F1	1.240669475	7.734122339	4.591758896	1.10E-05	0.000190647	up
GSTM1	-0.612639881	9.654904608	-4.590102676	1.11E-05	0.000191499	down
DCSTAMP	1.060030997	6.00384829	4.589072118	1.11E-05	0.000192158	up
CSAD	-0.50221554	7.91591093	-4.587915664	1.12E-05	0.000192627	down
TMEM200B	0.900619201	6.430113381	4.586186873	1.12E-05	0.000193548	up
PPARGC1A	-0.720244452	6.093219946	-4.584237588	1.13E-05	0.000194696	down
FAM110B	-0.512777802	6.078105717	-4.584175435	1.13E-05	0.000194696	down
DOK1	0.505821292	7.212066826	4.577173288	1.17E-05	0.000198967	up
SUMF2	-0.511907167	10.58359788	-4.576553014	1.17E-05	0.00019932	down

MANSC1	0.590956435	10.33153446	4.575493165	1.17E-05	0.000199732	up
LOC101928386	0.97642072	2.730995219	4.5736327	1.18E-05	0.000201097	up
SLC6A13	-0.734739432	8.139292632	-4.573421858	1.18E-05	0.000201112	down
MFAP2	-0.737553245	7.514077573	-4.569814855	1.20E-05	0.000203178	down
GTF2IRD2B	-0.782744031	7.51246342	-4.564700436	1.23E-05	0.00020681	down
SPRY1	-0.500748548	8.387820382	-4.562031901	1.24E-05	0.000208746	down
RHOBTB2	-0.667142106	6.141674775	-4.560588349	1.25E-05	0.00020968	down
CD163L1	0.951000392	5.415574086	4.558270366	1.26E-05	0.000211192	up
MSX1	0.703607513	5.455154494	4.554718141	1.28E-05	0.00021378	up
ARHGAP31	0.60496591	6.30818312	4.55414423	1.28E-05	0.000214121	up
LY96	1.036218564	7.83015664	4.553038899	1.29E-05	0.000214826	up
LOC100507053	0.724904283	6.073459605	4.552970174	1.29E-05	0.000214826	up
RASAL1	0.615779635	5.854704532	4.551633591	1.29E-05	0.000215833	up
STT3A	-0.635615015	9.761459882	-4.548250512	1.31E-05	0.000218648	down
MMP9	0.731757131	7.387508562	4.546987176	1.32E-05	0.000219607	up
RNASE6	1.121957605	7.239498019	4.544307498	1.33E-05	0.000221838	up
EGFR	-0.59783621	9.417027391	-4.53487614	1.38E-05	0.000228951	down
LOC100506558	0.85002532	6.368769369	4.530268417	1.41E-05	0.000232574	up
MATN2	0.85002532	6.368769369	4.530268417	1.41E-05	0.000232574	up
GSTM2	-0.554293309	10.20806061	-4.527917034	1.42E-05	0.000234452	down
LOC283588	-0.903860859	6.602392655	-4.527144493	1.43E-05	0.000235015	down
DOCK8	0.520371579	7.846499286	4.522347339	1.46E-05	0.000238915	up
NOVA1	0.859205278	5.489944483	4.519755817	1.47E-05	0.000240904	up
PLD4	0.763771005	5.332582418	4.518496965	1.48E-05	0.000241955	up
ADAMTS8	0.791879926	6.406061226	4.516986414	1.49E-05	0.000243257	up
CLCF1	-0.666607306	7.125625088	-4.516654281	1.49E-05	0.000243409	down
ANKRD36BP2	-0.59131566	6.24908799	-4.516336423	1.49E-05	0.000243546	down
SECTM1	0.709702451	8.199761871	4.511979093	1.52E-05	0.000247502	up
LOC340184	0.86798481	6.236555414	4.510427388	1.53E-05	0.000248697	up
FOXA3	0.704759895	8.497298484	4.509548109	1.53E-05	0.00024934	up
THSD4	-0.535196646	7.643184647	-4.504401579	1.57E-05	0.000252815	down
FJX1	-0.779760345	7.176040232	-4.5035338	1.57E-05	0.00025334	down
KAZALD1	0.559920923	6.39216171	4.502970875	1.57E-05	0.000253556	up
PRKAG2-AS1	-0.57658852	5.941741841	-4.50218341	1.58E-05	0.000254181	down
SEMA5B	-1.115152538	5.789815327	-4.501855568	1.58E-05	0.000254337	down
DCTN1-AS1	0.838535194	6.739323551	4.497345266	1.61E-05	0.000258441	up
CYTIP	0.752668104	8.117362035	4.49344631	1.64E-05	0.000261966	up
MIR302B	0.810555043	7.291690307	4.49325217	1.64E-05	0.000261987	up
FAM218A	-0.631070913	6.470864931	-4.488631527	1.67E-05	0.000266702	down
S1PR5	-0.861701895	4.85266079	-4.487971122	1.67E-05	0.000267036	down
LOC100505938	0.500014478	8.293444483	4.487686023	1.67E-05	0.000267154	up
LOC283454	0.776487145	6.088414822	4.486097694	1.68E-05	0.000268486	up
PLAUR	0.731509614	8.094103763	4.484905378	1.69E-05	0.000269584	up
RNF175	0.595437707	7.146444407	4.483674843	1.70E-05	0.000270521	up
VSIG4	1.325380399	9.204710016	4.472952325	1.78E-05	0.000280518	up
VMO1	-0.804278542	10.20383949	-4.465396819	1.83E-05	0.00028787	down
SCML1	-0.522248178	8.493759235	-4.463452043	1.84E-05	0.000289718	down
LAG3	-1.026303174	6.205237933	-4.462499964	1.85E-05	0.000290293	down
PI3	-0.968884971	8.804391281	-4.462439994	1.85E-05	0.000290293	down
VSIG2	0.526470218	9.324283243	4.459581294	1.87E-05	0.000293225	up
FOS	-1.454017341	11.12534301	-4.44850802	1.96E-05	0.000305013	down
DLGAP1-AS2	0.570158789	6.62220865	4.446982792	1.97E-05	0.000306665	up
FAM167A-AS1	-1.149601051	6.489724626	-4.44565564	1.98E-05	0.000308082	down
LOC100996741	-0.865946429	7.138830727	-4.436428419	2.05E-05	0.000318292	down
PRKG1	0.660486236	5.542646126	4.435933259	2.06E-05	0.000318336	up
LYPD6	-0.659132554	5.381928773	-4.43563807	2.06E-05	0.000318457	down
CCEPR	1.001916106	5.290135727	4.433126241	2.08E-05	0.000321439	up
DNAJC17	-0.527214265	8.277271587	-4.426202846	2.14E-05	0.000329745	down
PDZK1IP1	-0.676856868	9.126438583	-4.423367038	2.16E-05	0.000333258	down
TFEB	-0.502485508	8.447123891	-4.405977415	2.32E-05	0.000355873	down
MAGED4	0.760973194	6.771774304	4.401989459	2.36E-05	0.00035865	up

MAGED4B	0.760973194	6.771774304	4.401989459	2.36E-05	0.00035865	up
SNORA11D	0.760973194	6.771774304	4.401989459	2.36E-05	0.00035865	up
SNORA11E	0.760973194	6.771774304	4.401989459	2.36E-05	0.00035865	up
WTIP	-0.945186138	6.579280399	-4.399671623	2.38E-05	0.000361476	down
NTN5	-0.674341406	5.875830454	-4.397808638	2.40E-05	0.00036391	down
ITGAX	1.019026699	5.73415366	4.393453885	2.44E-05	0.00036901	up
ADIRF-AS1	-0.792605281	6.680372958	-4.389914296	2.47E-05	0.000373223	down
TMEM200A	0.7597652	7.217638094	4.389568692	2.47E-05	0.000373419	up
CPVL	0.514507006	8.707819826	4.381502712	2.55E-05	0.000383743	up
ITPRIPL1	-0.723922092	6.311729984	-4.379917001	2.57E-05	0.000385713	down
ACPP	0.531096536	6.060928256	4.37842467	2.59E-05	0.00038723	up
ZNF821	-0.560514855	5.596637864	-4.375626377	2.61E-05	0.000391274	down
CIC	-0.600058363	8.33995716	-4.374577119	2.63E-05	0.00039264	down
BACH2	0.558613054	6.030609266	4.372701254	2.65E-05	0.00039504	up
HEYL	-0.556987334	6.258254961	-4.370639021	2.67E-05	0.000397487	down
LINC01082	1.02504125	4.889548154	4.367170179	2.70E-05	0.000402435	up
PER3	-0.55974304	7.626925696	-4.363876949	2.74E-05	0.000407161	down
NPDC1	0.668751182	8.778737526	4.360573202	2.77E-05	0.000412226	up
OSTM1-AS1	0.967283097	3.314604959	4.353186955	2.86E-05	0.000423559	up
TNFRSF25	-0.593690136	7.044556789	-4.351564388	2.87E-05	0.000425718	down
CATSPERB	0.648236912	6.544249878	4.348154376	2.91E-05	0.000431184	up
LOC100287497	-0.61052616	8.040955102	-4.336549206	3.05E-05	0.000448675	down
CHSY3	-1.039419784	4.894227246	-4.332077141	3.10E-05	0.000455149	down
GEM	-0.86412168	7.826160605	-4.329176496	3.14E-05	0.00045947	down
AOC1	0.969484567	5.877266634	4.325308666	3.19E-05	0.000464721	up
AMDHD2	-0.866823088	6.095466752	-4.32481786	3.19E-05	0.000465197	down
LOC101927609	0.526348192	8.021392628	4.323134534	3.21E-05	0.000466885	up
IL1RN	0.802913025	6.998488853	4.312870327	3.35E-05	0.000482338	up
HSPB1	-0.505706895	12.55274677	-4.309544368	3.39E-05	0.000486806	down
SMIM1	-1.053159773	5.884575385	-4.306079383	3.44E-05	0.000492204	down
SCNN1G	-0.514780233	8.913374411	-4.303937435	3.46E-05	0.000495333	down
RAB5B	-0.525032464	10.03735306	-4.301063885	3.50E-05	0.000500056	down
BHLHB9	-0.524804161	7.155445431	-4.300194971	3.52E-05	0.000501442	down
ZNF251	-0.606102359	7.999816495	-4.29995661	3.52E-05	0.000501595	down
COCH	0.524047549	9.648855167	4.299706347	3.52E-05	0.000501772	up
POU5F1P4	-0.756076653	6.35434542	-4.29730302	3.56E-05	0.000506184	down
MEIS3	-0.66849391	6.566117217	-4.296438253	3.57E-05	0.000507578	down
PMP22	0.602202161	6.007051627	4.292768389	3.62E-05	0.000513641	up
CFD	-0.915548181	9.547483	-4.291189879	3.64E-05	0.000516142	down
ZNF649	-0.916206269	6.177360681	-4.287250413	3.70E-05	0.000523152	down
TBX6	-0.624465422	5.952690576	-4.283762266	3.75E-05	0.000528996	down
CSGALNACT1	-0.678334054	6.78058681	-4.283131841	3.76E-05	0.000529314	down
TMOD2	-0.615054441	6.425184759	-4.282191017	3.77E-05	0.000530275	down
FXYD1	-0.637958863	9.711001094	-4.277403188	3.84E-05	0.00053925	down
DLK2	-0.696831667	8.030915753	-4.274687987	3.88E-05	0.000544305	down
HS3ST3B1	0.51063059	7.913308346	4.274496805	3.89E-05	0.000544375	up
DOPEY2	0.503654183	7.264392598	4.268266566	3.98E-05	0.000554323	up
CD53	0.71494296	8.669981878	4.264674201	4.04E-05	0.000561417	up
CDH3	-0.689725087	9.31550432	-4.258242335	4.14E-05	0.00057212	down
ATP10B	0.6904204	7.579120245	4.255679941	4.18E-05	0.000575741	up
MTUS2	-0.548512486	6.030894773	-4.249470662	4.28E-05	0.000587635	down
ZNF527	-0.627873338	6.158104475	-4.245339503	4.35E-05	0.000596035	down
NBL1	0.510270656	10.18107945	4.244176669	4.37E-05	0.000598365	up
SAMD1	-0.837767691	6.988525337	-4.240717088	4.43E-05	0.000605701	down
SMIM10L2A	-0.582691106	5.887833694	-4.238121633	4.47E-05	0.00061108	down
SORCS1	0.605467857	5.438861125	4.237027788	4.49E-05	0.000612936	up
LRP1	-0.657961458	6.412943825	-4.235586133	4.52E-05	0.000615258	down
FUT3	0.55177729	8.588302841	4.232285706	4.58E-05	0.000622042	up
CNTD1	-0.5309285	10.08858901	-4.232080032	4.58E-05	0.000622166	down
FSCN1	0.626869699	5.627975467	4.227514974	4.66E-05	0.000632102	up
ATP6V0D2	0.510758981	6.339505787	4.226438438	4.68E-05	0.000633981	up

WDR72	0.607321175	5.943770577	4.220876387	4.78E-05	0.000645816	up
CFB	-0.571887577	10.25725576	-4.211163994	4.96E-05	0.000667679	down
LOC652276	-0.952434155	6.212301583	-4.208697589	5.01E-05	0.000671999	down
BCL2L15	0.50847215	7.153018291	4.206146969	5.06E-05	0.000677108	up
KRT6A	1.249608861	5.992884121	4.204681554	5.09E-05	0.000680537	up
ANXA8	-0.622651222	9.552582846	-4.203939932	5.10E-05	0.000680973	down
ANXA8L1	-0.622651222	9.552582846	-4.203939932	5.10E-05	0.000680973	down
LOC339803	0.550173581	7.008216495	4.202989092	5.12E-05	0.000682982	up
COLEC12	0.809358362	7.353608116	4.202079234	5.14E-05	0.000684177	up
TIMP3	0.540688028	6.991238895	4.199348997	5.20E-05	0.000688587	up
DCAKD	-0.524323544	7.658328989	-4.197026705	5.24E-05	0.000693953	down
ELF5	-0.622303227	8.552872573	-4.187578997	5.44E-05	0.000716263	down
BAIAP3	0.610442599	9.605632082	4.18620023	5.46E-05	0.000719232	up
CCL7	1.043846814	4.349059263	4.179043011	5.62E-05	0.000736689	up
CKB	-0.503114088	11.59592522	-4.171785478	5.77E-05	0.000754412	down
HSD17B2	-1.377046652	6.54337945	-4.166798301	5.89E-05	0.000765166	down
IER2	-0.646489171	11.87317228	-4.166699818	5.89E-05	0.000765166	down
TUBB6	0.821865812	7.671428447	4.166446326	5.89E-05	0.000765166	up
ZIK1	-0.513000182	6.632172763	-4.161631663	6.00E-05	0.000777493	down
SOX9-AS1	-0.63940167	6.27025112	-4.158910093	6.07E-05	0.000783512	down
MCC	-0.568184638	8.052557304	-4.156984107	6.11E-05	0.000788404	down
GHR	-1.095589911	6.515694995	-4.156577465	6.12E-05	0.000789123	down
NTF3	-1.161381077	6.189824803	-4.156282152	6.13E-05	0.000789123	down
ZC4H2	0.819129502	6.181936342	4.156154057	6.13E-05	0.000789123	up
LEPR	-0.602996962	6.200558617	-4.152345407	6.22E-05	0.000798786	down
PYGO1	-0.714808416	5.391408772	-4.151645289	6.24E-05	0.000799828	down
FGFBP2	0.941580069	6.783245696	4.145789028	6.38E-05	0.000813153	up
TEX22	-0.724542516	6.01245978	-4.140400352	6.51E-05	0.000828168	down
CCL17	-0.766110002	6.466995605	-4.136847788	6.60E-05	0.000838044	down
BRSK2	-0.679269937	4.422357786	-4.136080063	6.62E-05	0.000839881	down
PDE8B	0.518982527	9.262713803	4.135891504	6.62E-05	0.000839881	up
MCOLN2	-0.577434014	6.553987787	-4.123408115	6.94E-05	0.000874677	down
TSHB	-0.927346386	5.165858388	-4.122299607	6.97E-05	0.000877883	down
KIR2DL3	-0.759604277	5.056018135	-4.121286885	7.00E-05	0.000880294	down
FCGR2A	0.823499138	6.895371855	4.114877415	7.17E-05	0.000898501	up
KCNJ1	-1.047077193	5.60850489	-4.107716173	7.37E-05	0.000921706	down
STATH	1.431872904	5.218306162	4.104480913	7.46E-05	0.000931104	up
LINC00494	-0.794609001	6.104426806	-4.103065941	7.50E-05	0.000935518	down
PHLDA2	0.648361335	7.079921022	4.099965179	7.59E-05	0.000943983	up
IKZF3	-0.670416841	5.632503369	-4.097994474	7.65E-05	0.000947948	down
REEP1	0.630315797	10.30147197	4.093505801	7.78E-05	0.000962084	up
TRIM7	0.708042116	7.37826836	4.090629724	7.86E-05	0.000971018	up
ARHGEF26-AS1	0.549363441	8.784384693	4.089926367	7.88E-05	0.000973075	up
ATAD2	-0.508843001	6.820660629	-4.087525262	7.95E-05	0.000981408	down
PANX3	1.035904091	3.88541776	4.08681604	7.98E-05	0.000982978	up
FIGF	0.944147452	5.493857132	4.078983296	8.22E-05	0.00100433	up
PIR-FIGF	0.944147452	5.493857132	4.078983296	8.22E-05	0.00100433	up
PHYHIPL	0.660912384	6.084497982	4.074577064	8.35E-05	0.001019298	up
PAQR5	0.580250724	6.103426867	4.07449087	8.36E-05	0.001019298	up
C3orf67	0.579913577	8.317588957	4.072915592	8.41E-05	0.001024602	up
CEACAM19	-0.990275403	6.16565178	-4.072829958	8.41E-05	0.001024602	down
C15orf48	0.959368821	9.541664446	4.070377157	8.49E-05	0.001033008	up
C16orf54	0.641626584	9.544699873	4.063853673	8.70E-05	0.00105251	up
CROCCP3	-0.583324505	7.141411962	-4.059507268	8.84E-05	0.001068154	down
MEF2C	-0.521155494	6.752243953	-4.058777675	8.86E-05	0.001069423	down
PLAG1	-0.600405307	8.374373818	-4.057860448	8.90E-05	0.001072515	down
SIGLEC11	0.640084484	5.811037178	4.057611579	8.90E-05	0.001072952	up
POU5F1P3	-0.689061616	6.322618038	-4.055298977	8.98E-05	0.001081173	down
UMODL1	0.739801109	6.72640104	4.053532285	9.04E-05	0.001084538	up
VANGL2	-0.585908613	8.746054065	-4.052931088	9.06E-05	0.001085282	down
DQX1	0.670560343	6.387042614	4.052888868	9.06E-05	0.001085282	up

CHCHD10	-0.604688764	10.41667886	-4.052603276	9.07E-05	0.001085877	down
ZFP2	-0.78695637	6.935584424	-4.051616652	9.11E-05	0.001088224	down
SERPINA6	1.215781136	7.421747682	4.048984206	9.20E-05	0.001096704	up
TTN-AS1	-0.54074511	7.470781525	-4.04771418	9.24E-05	0.001099645	down
NCF1	0.973503719	6.67512755	4.042813183	9.41E-05	0.001115391	up
EHBP1L1	-0.529361081	7.786525883	-4.041728506	9.45E-05	0.001118684	down
NLRP12	0.680966397	6.043110993	4.039669501	9.52E-05	0.001125688	up
ALDH4A1	-0.651448813	6.943560836	-4.036554868	9.64E-05	0.001135957	down
LOC101929180	-0.886461576	5.284913962	-4.031875976	9.81E-05	0.001153645	down
UFSP1	-0.832745317	6.425953317	-4.030860906	9.84E-05	0.001157438	down
SULT2B1	-0.520116138	8.660498717	-4.027891349	9.95E-05	0.001167359	down
BIRC3	-0.554848046	8.122575656	-4.027348347	9.97E-05	0.001167932	down
RRN3P2	-0.503732905	7.945074782	-4.018821076	0.00010296	0.001201612	down
LINC00595	0.744592399	6.693582959	4.018075182	0.000103247	0.001202934	up
LOC101928100	0.605064463	7.682586008	4.016071076	0.000104022	0.001211348	up
IFITM3	-0.565049793	11.7489966	-4.014567898	0.000104607	0.001217541	down
FHL1	0.732354579	8.407464293	4.007529719	0.000107389	0.001245985	up
MIRLET7D	-0.648846372	8.205830943	-4.004426313	0.000108638	0.00125804	down
COL4A6	-0.788887706	6.991342686	-3.996346657	0.000111955	0.001292516	down
CARF	-0.559571682	7.935974663	-3.991296821	0.000114077	0.001315683	down
EPHB1	0.68267052	5.433107193	3.990441124	0.00011444	0.001318542	up
MPEG1	0.66454918	7.728004104	3.981096218	0.00011848	0.001358925	up
GLS2	-0.608448058	5.79055663	-3.980838913	0.000118594	0.001359341	down
BTG2	-0.623284679	10.29053507	-3.978808239	0.00011949	0.001367075	down
DNAJC6	0.593946331	8.644244367	3.976699012	0.000120429	0.00137643	up
CEL	0.859927329	8.985172981	3.976339411	0.000120589	0.001376888	up
TAZ	-0.51648066	7.371837751	-3.970107076	0.000123406	0.001404523	down
LOC280665	0.724800073	5.803272875	3.970033217	0.00012344	0.001404523	up
XXYLT1	-1.104005161	6.095091182	-3.965997208	0.000125298	0.001422124	down
MIR205HG	-0.923381202	8.105681774	-3.963681628	0.000126376	0.001432208	down
FAM153B	0.576307801	7.098892434	3.960453514	0.000127894	0.001443694	up
LOC100507387	0.576307801	7.098892434	3.960453514	0.000127894	0.001443694	up
FIGN	0.521797239	5.210097185	3.9574327	0.00012933	0.001457894	up
TEPP	0.701718072	6.15169584	3.957100952	0.000129489	0.001458811	up
SERPINB9	0.575703039	7.004864929	3.955600201	0.000130209	0.001464034	up
CTHRC1	0.982945511	4.94683267	3.954372943	0.000130801	0.001469962	up
CCRL2	0.983104267	6.692467905	3.953271908	0.000131334	0.0014745	up
SCEL	0.911131135	5.113231785	3.951560578	0.000132166	0.001482389	up
PIK3R2	0.617517553	10.38390454	3.950898711	0.000132489	0.001484557	up
LOC101243545	0.703973397	9.262475427	3.947767219	0.000134029	0.001499605	up
SNORD4A	0.703973397	9.262475427	3.947767219	0.000134029	0.001499605	up
CASQ2	0.832795092	4.678385376	3.944038554	0.000135885	0.001514435	up
LOC100288675	0.828647782	6.240510039	3.943493408	0.000136159	0.00151456	up
FBP1	0.680714368	9.812045143	3.943190652	0.000136311	0.00151456	up
ZNF76	-1.027279031	6.823546733	-3.940957533	0.000137437	0.00152379	down
CLDN16	-0.701035223	7.726967158	-3.931656513	0.000142226	0.001568261	down
TMPRSS11E	0.922099975	5.187320297	3.931483606	0.000142316	0.001568502	up
SFN	0.689102408	10.88191375	3.927793117	0.000144261	0.001587639	up
JAK2	-0.501726879	8.074379259	-3.927577245	0.000144376	0.001587641	down
SLC13A2	-0.756654989	6.698619627	-3.926804643	0.000144787	0.001591119	down
MGC32805	-0.615027621	5.3679696	-3.926322451	0.000145043	0.001592409	down
ANHX	-0.871245226	5.414265364	-3.9174841	0.000149829	0.001636301	down
PTRF	-0.601488063	7.350719171	-3.917052351	0.000150067	0.001638113	down
FAM186A	0.703236387	4.881638385	3.913318233	0.000152137	0.00165754	up
SYT4	0.849136424	5.492513877	3.911842832	0.000152963	0.001664392	up
IRF8	0.573261385	8.463920888	3.907479341	0.000155429	0.001688569	up
FAM153A	0.525848253	6.9005049	3.905247549	0.000156705	0.001698391	up
HSF1	-0.631351919	7.620075726	-3.901320055	0.000158974	0.001718098	down
OR10J1	-0.791619839	5.237758731	-3.899098897	0.000160272	0.001730482	down
POU5F1	-0.878978505	6.644860659	-3.897443934	0.000161245	0.001738525	down
KCNH8	1.023099206	4.594905426	3.896676987	0.000161698	0.001740524	up

PATL2	-0.50493518	8.560107551	-3.896613877	0.000161735	0.001740524	down
CHRNA9	-0.579748659	7.530239712	-3.889612968	0.000165927	0.001781337	down
TM4SF1	0.638863815	9.368179019	3.888362677	0.000166687	0.001786458	up
EXD1	0.916126956	3.596621329	3.887073134	0.000167474	0.001792141	up
LINC00939	-0.618071547	7.419025623	-3.884350404	0.000169146	0.001806159	down
TMEM45A	-1.109348412	11.09473691	-3.882332649	0.000170396	0.001813221	down
REG3G	0.86374666	4.814464652	3.88173083	0.000170771	0.001814259	up
FLJ25758	-0.732875767	5.71881727	-3.877326907	0.000173535	0.001838058	down
MGC70870	0.570323637	7.773166484	3.868361149	0.000179296	0.00188595	up
ZGLP1	-0.562959333	7.393969546	-3.852154091	0.000190173	0.001983919	down
WAS	0.534913193	8.259249424	3.851637116	0.00019053	0.00198583	up
PRKCB	-0.785966293	7.190917015	-3.85125218	0.000190797	0.001986792	down
TTPA	-0.933753381	4.740589478	-3.84352149	0.000196221	0.002035846	down
PTPRH	0.987260355	6.177671868	3.84288002	0.000196677	0.002039657	up
BTBD19	-0.506767836	5.928442036	-3.842272083	0.000197111	0.002043226	down
LOC102725271	0.6490135	5.128261458	3.840042051	0.00019871	0.002055132	up
CPNE9	0.641562204	6.47172423	3.836302672	0.000201418	0.002079375	up
STAP1	0.55774362	7.190153233	3.830363636	0.000205791	0.002110929	up
LRP2	-0.73122624	6.733379996	-3.830101132	0.000205987	0.002110979	down
LRP4	0.547466889	8.088355786	3.829089084	0.000206742	0.00211711	up
MSMB	0.535841322	14.73543605	3.828854678	0.000206917	0.002117954	up
SLAMF8	0.77847951	6.500994931	3.823827035	0.000210709	0.002149562	up
TEX41	0.6784354	4.493779502	3.821507107	0.000212482	0.002163278	up
FRMD8P1	-0.725047204	6.044919393	-3.810038053	0.000221453	0.002235988	down
SGTB	0.572013227	6.641613908	3.809081303	0.000222218	0.002241414	up
FAM166A	0.677044291	5.989052984	3.807804712	0.000223242	0.002249754	up
SRGN	0.823033145	10.62761005	3.802180216	0.000227806	0.002286676	up
LOC100505874	-0.869393609	6.837794564	-3.797562885	0.00023162	0.002319858	down
NTS	-1.351553544	11.02500739	-3.796350329	0.000232632	0.002326647	down
GTF2A1L	0.760837697	4.303470016	3.793973167	0.000234627	0.002337932	up
CA14	-0.994864078	5.237281003	-3.793941812	0.000234653	0.002337932	down
LOC101928614	0.91556477	4.482455663	3.79226508	0.00023607	0.002345596	up
LOC103611081	0.503881991	7.901762448	3.791671513	0.000236574	0.002348641	up
ZFPM2	-0.982578041	4.796203441	-3.790396468	0.000237659	0.002355552	down
SYT8	0.76902867	7.404173499	3.789515301	0.000238412	0.002360964	up
CD83	0.528238498	8.760223634	3.78673433	0.000240803	0.002380356	up
NINJ1	-0.508751453	8.765029473	-3.784988012	0.000242316	0.002392361	down
CACNA2D3	0.800347588	5.991643258	3.784231939	0.000242974	0.002397819	up
CORO1C	0.550360997	9.451098791	3.783300646	0.000243787	0.0024048	up
CDH10	0.731145038	5.593997332	3.781364138	0.000245485	0.002418416	up
ZNF619	-0.653436542	6.473044748	-3.778434081	0.000248075	0.002438677	down
P3H3	-0.968892781	5.242851283	-3.777308106	0.000249078	0.002447477	down
POLD1	-0.53547296	7.698660077	-3.774962857	0.000251178	0.002464933	down
C4orf17	0.778060341	5.769403492	3.774151732	0.000251908	0.002469977	up
MIR205	-0.639858731	8.015162527	-3.772328366	0.000253557	0.002482948	down
NRSN2-AS1	0.55740823	7.274539048	3.77111477	0.000254661	0.002490547	up
APBA2	-0.606421465	6.126563284	-3.770192483	0.000255502	0.002497707	down
HLA-DOB	-0.632148582	5.445077568	-3.76741351	0.000258053	0.002518337	down
AAAS	-0.567649205	8.198187957	-3.759766904	0.000265199	0.002572683	down
LINC00630	0.690186082	4.867659885	3.755825491	0.000268956	0.002592602	up
TAS2R1	0.855856812	5.435088204	3.748456284	0.000276115	0.002647823	up
NRCAM	0.51024112	5.782880965	3.748378194	0.000276192	0.002647823	up
NCF1B	1.18253855	6.271517906	3.746136905	0.000278405	0.00266457	up
NCF1C	1.18253855	6.271517906	3.746136905	0.000278405	0.00266457	up
MST1	-0.572887916	8.744023564	-3.745224948	0.000279311	0.002670997	down
HLA-DQB2	-0.600105657	7.451635326	-3.744005879	0.000280526	0.002680369	down
EIF5A	-0.676288805	9.7822934	-3.743167345	0.000281364	0.002687257	down
RGN	-0.590499669	6.674626222	-3.742514173	0.000282019	0.00269126	down
SERPINB10	-1.036396311	7.275973874	-3.740488465	0.000284059	0.002707332	down
BCAM	-0.555923276	8.010058152	-3.739096449	0.000285469	0.002716577	down
TFEC	0.87736254	5.403394397	3.72960214	0.000295264	0.002793226	up

CXCL8	0.74280864	11.27468429	3.729267229	0.000295615	0.002795306	up
STAC	0.809068084	7.379243043	3.72623091	0.000298818	0.002820918	up
PDGFD	-0.600470569	6.198984657	-3.724316148	0.000300854	0.002837098	down
NCAPG	0.611539854	5.711671733	3.721460312	0.000303916	0.002859594	up
TMEM117	0.882597024	6.665656075	3.717911168	0.000307763	0.002889833	up
JAML	0.56848334	8.373902836	3.716796176	0.000308981	0.002898886	up
LINC01410	0.571832133	6.895739609	3.712153449	0.000314102	0.002940067	up
ORM2	0.7408392	4.273706767	3.708156148	0.000318575	0.002975449	up
LONRF2	-0.507916247	7.929930693	-3.704754941	0.000322428	0.003005295	down
MMP10	-1.283032724	8.476298438	-3.701423731	0.000326245	0.003037154	down
NR2F2-AS1	-0.994907389	7.796937239	-3.698744565	0.000329346	0.003056056	down
PXN-AS1	-0.788141433	6.554060599	-3.689382217	0.000340403	0.003142058	down
TMEM25	-0.508332902	6.641798782	-3.68727415	0.000342941	0.00315528	down
LOC100506125	-0.872449697	4.667914649	-3.685740233	0.000344799	0.003167268	down
MPPED2	0.660240995	6.260172585	3.685494866	0.000345097	0.003168731	up
C10orf128	0.601034826	7.142935556	3.683543092	0.000347477	0.003186254	up
RUSC2	-0.867051876	5.804536714	-3.683472081	0.000347563	0.003186254	down
LINC01393	0.828026497	4.647594146	3.682538148	0.000348708	0.003193179	up
AKAP4	0.89021055	4.7424086	3.681239786	0.000350305	0.003204062	up
RPL39L	-0.554633177	7.225080799	-3.68029754	0.000351468	0.00321302	down
PCDH8	0.997438056	3.631217625	3.678583136	0.000353594	0.003228577	up
SLC34A2	-0.592469814	11.534741	-3.677567681	0.000354859	0.003236245	down
LIPF	-0.809088515	4.9513023	-3.67687892	0.000355719	0.003242796	down
PXDN	0.574023743	6.547686255	3.67109823	0.000363019	0.003297493	up
MCF2L-AS1	-0.536852854	7.302785581	-3.664106555	0.000372037	0.003364688	down
SLC28A3	-0.542671475	7.185437926	-3.66345605	0.000372887	0.003371006	down
IFI30	0.796311151	11.71686434	3.66313188	0.000373311	0.003372071	up
FST	-0.572767022	6.154608132	-3.662917504	0.000373592	0.003372071	down
SNAI2	-1.01591794	7.903329276	-3.655525013	0.000383396	0.003444229	down
NOC4L	-0.844415702	7.323858928	-3.655303514	0.000383693	0.003445546	down
GAPLINC	0.848488326	5.594710342	3.654161219	0.000385231	0.003456636	up
SPRR1A	0.79105029	6.265296399	3.65354556	0.000386062	0.003462733	up
LOC283194	-0.718286137	5.339985531	-3.652792488	0.000387081	0.003469751	down
TBXAS1	0.507867686	6.272718902	3.646933745	0.000395096	0.003521629	up
BTK	0.8707159	6.640570395	3.646132402	0.000396204	0.003528753	up
IL31RA	0.609451436	4.284807396	3.645970412	0.000396429	0.003529375	up
SCD	-0.570661022	8.461483585	-3.643512798	0.000399848	0.003554276	down
FRRS1	0.928520973	4.411554245	3.638373796	0.000407089	0.003613012	up
TNFAIP2	-0.545966707	8.931267967	-3.635322572	0.000411446	0.003647433	down
PIRT	0.762792382	4.54149987	3.63392283	0.00041346	0.003662441	up
LOC284630	-0.833754581	6.263686767	-3.63217856	0.000415982	0.003679076	down
TTC34	-0.833754581	6.263686767	-3.63217856	0.000415982	0.003679076	down
UBD	-0.717523822	10.08801935	-3.627471171	0.000422863	0.003734143	down
ADGRE5	0.501675692	10.56317411	3.626220927	0.000424708	0.003746092	up
CLCNKA	0.551078867	6.982918631	3.621707478	0.000431433	0.003796159	up
LOC340357	-0.752617693	3.844742863	-3.621631181	0.000431548	0.003796159	down
DPP10	0.769744646	6.466205793	3.619349148	0.000434987	0.003817793	up
SLC6A20	-0.676880553	6.550739209	-3.61932565	0.000435023	0.003817793	down
DPY19L2	-0.586694054	9.149945454	-3.619223468	0.000435178	0.003817793	down
GOLT1A	-0.789838406	6.083113855	-3.617846899	0.000437266	0.00382729	down
LOC254028	-0.722383836	4.905618556	-3.615202322	0.000441304	0.003858199	down
HDAC7	-0.59765161	6.603592743	-3.610312651	0.000448863	0.0039138	down
RNASE2	0.760640554	5.803358711	3.609531354	0.000450082	0.00391994	up
POLQ	-0.527273109	7.812372964	-3.605917074	0.000455763	0.003966489	down
PTPRD	0.533104966	5.354034803	3.60590979	0.000455775	0.003966489	up
ZNF154	-0.599476741	5.188445596	-3.603812063	0.000459103	0.003989372	down
MS4A4A	0.874382778	7.711632868	3.603450368	0.000459679	0.003992859	up
LOC647115	0.762771571	6.741435892	3.602711287	0.000460858	0.004000061	up
BMP4	1.259798524	5.508147437	3.601305701	0.000463109	0.004015718	up
PFKFB4	-0.606709006	5.897637171	-3.60109134	0.000463453	0.004016479	down
BIRC7	0.588403472	6.712612759	3.59847681	0.000467671	0.004044591	up

FAM153C	0.620319746	8.166854865	3.597808087	0.000468756	0.004045541	up
LOC101928349	0.620319746	8.166854865	3.597808087	0.000468756	0.004045541	up
LOC101930363	0.620319746	8.166854865	3.597808087	0.000468756	0.004045541	up
TTTY1	0.569470807	5.700808811	3.592462503	0.000477511	0.004108681	up
TTTY1B	0.569470807	5.700808811	3.592462503	0.000477511	0.004108681	up
CD44	0.537823092	9.217764267	3.591877366	0.000478479	0.004113908	up
GPR153	0.502266043	7.333865283	3.589778157	0.000481966	0.004141841	up
SPATA45	-0.71170673	6.152130006	-3.589703381	0.00048209	0.004141841	down
FAM178B	0.566193128	6.936513985	3.589124511	0.000483056	0.004148039	up
DPY19L2P4	-0.95998564	5.140323307	-3.586511718	0.000487439	0.004178364	down
NCKAP5	-1.099969427	3.640190996	-3.586145866	0.000488056	0.004180512	down
C15orf56	-0.689383186	6.397163999	-3.584858201	0.000490232	0.004192246	down
HELLS	-0.522033954	6.523606916	-3.583963474	0.00049175	0.004201124	down
ITGA5	0.956924198	6.451480258	3.580143689	0.000498279	0.004241039	up
LOC105372526	-0.528288385	7.612535256	-3.579892746	0.000498711	0.004243133	down
GAS1RR	-0.72031711	5.342824897	-3.575621762	0.000506115	0.004299715	down
PLG	0.892571897	6.392922535	3.570481714	0.000515162	0.004363593	up
A2M	0.636420007	8.096209565	3.566559643	0.000522168	0.004414749	up
CNTFR	0.587955561	6.696524551	3.566058313	0.00052307	0.004420739	up
DUOXA2	0.994531222	6.217512614	3.560967589	0.000532313	0.004480615	up
GUCY2F	-0.736338925	4.354100233	-3.559190512	0.000535576	0.004502447	down
SLC44A5	-0.995484353	7.286968285	-3.557654043	0.000538412	0.004521949	down
STAU2-AS1	-0.524145587	6.784140176	-3.554110468	0.000545007	0.004572294	down
GPR65	0.70964742	7.246396133	3.553244067	0.000546631	0.004584235	up
PTGS2	0.740275587	6.661501054	3.547457648	0.000557595	0.004662485	up
NLRP3	0.608537004	3.997566695	3.546742888	0.000558964	0.004672219	up
SHF	-0.782983936	5.288321101	-3.543788999	0.000564654	0.004712878	down
ADRA2A	0.703719267	9.297850252	3.539111371	0.000573776	0.004775052	up
TRAIP	-0.870984577	4.268699772	-3.536702366	0.000578528	0.004802347	down
DOC2A	-0.729842969	7.726001673	-3.536018057	0.000579884	0.004808364	down
SCG5	1.007436358	5.337944297	3.532343462	0.00058722	0.004858609	up
SOWAHA	0.753026298	7.221630148	3.528250277	0.000595495	0.004911255	up
USP27X-AS1	-0.531972061	6.980032332	-3.528027284	0.000595949	0.004911255	down
TM6SF1	0.622609432	6.823311207	3.526690281	0.000598677	0.004930182	up
SHCBP1	0.858622878	4.979797686	3.524662772	0.000602838	0.004960864	up
CLEC7A	0.588101678	6.848235242	3.522167168	0.000607996	0.004994312	up
ZNF549	-0.957385485	4.243785721	-3.513238217	0.000626793	0.005124079	down
TMEM221	-0.601931544	5.151510468	-3.512363632	0.000628663	0.005130864	down
PRIM2B	-0.514596839	6.216837032	-3.508713318	0.000636527	0.005183925	down
LOC285423	0.827206681	4.106256585	3.508351743	0.000637311	0.005188459	up
PROK2	1.191610646	6.221756168	3.505931305	0.000642582	0.005222063	up
LRRN3	0.555629046	5.578884947	3.504264807	0.000646235	0.005245841	up
PLAT	0.830764092	7.418081949	3.504019374	0.000646774	0.0052468	up
C20orf187	-0.717674712	5.112839195	-3.503732906	0.000647405	0.005250049	down
MTTP	0.502601727	6.509526874	3.503513777	0.000647887	0.005252097	up
LILRB2	0.768988015	6.060613803	3.499691361	0.000656361	0.005309477	up
NAB2	-0.514174382	6.674139888	-3.494794626	0.000667368	0.005387855	down
STON1-GTF2A1L	1.000077404	3.564987139	3.494508545	0.000668016	0.005388595	up
FUT1	-0.57549821	6.749630037	-3.492019429	0.000673683	0.005430366	down
SH2D1A	-0.506831612	4.914184322	-3.486511388	0.000686384	0.005511846	down
DMBT1	-0.572494747	8.191589582	-3.486484624	0.000686446	0.005511846	down
LOC100131043	-0.706561752	5.224936511	-3.483493998	0.000693437	0.005556257	down
FGF13	0.500140753	9.030567955	3.479079592	0.000703879	0.005626102	up
CD52	0.81198238	9.79902407	3.478041537	0.000706355	0.005639761	up
LOC105371920	0.508040786	7.796372244	3.476369699	0.000710361	0.005666153	up
MIR924HG	0.810613876	4.838322263	3.468794994	0.000728782	0.005796746	up
SLA	0.635869591	8.36481806	3.468567731	0.000729341	0.005799177	up
TP63	-0.855697139	7.556002918	-3.46731041	0.000732444	0.005819796	down
NR1I3	-0.583467279	4.676906892	-3.462437474	0.000744588	0.00590191	down
HES6	-0.81315123	7.822140327	-3.462273192	0.000745	0.005903133	down
LOC100128198	-0.670087118	5.012398002	-3.460635088	0.000749127	0.005923499	down

LOC107985971	-0.537218443	9.220955928	-3.446830349	0.000784768	0.006158654	down
TNFSF4	-0.602920535	6.20663188	-3.446468259	0.000785724	0.006161672	down
ST7-AS1	-0.692738252	5.825114233	-3.442833391	0.000795381	0.006226713	down
ADAM3A	0.68136268	4.635629528	3.44202506	0.000797544	0.006240616	up
INHBA	0.725595917	6.357544654	3.441773642	0.000798217	0.006242498	up
FGF20	-0.957244606	4.420104095	-3.440997803	0.0008003	0.006256643	down
DRD1	-0.832912768	6.009980707	-3.440061486	0.00080282	0.006272051	down
KDR	0.592011887	7.280892635	3.437620277	0.000809426	0.006317175	up
ZNF815P	-0.701897914	4.54782701	-3.434939429	0.000816739	0.00635254	down
ZNF890P	-0.701897914	4.54782701	-3.434939429	0.000816739	0.00635254	down
GFRA3	0.659179886	5.063495485	3.433685788	0.00082018	0.006377133	up
FHOD3	-0.728448538	9.139729418	-3.432294762	0.000824014	0.006400702	down
NFKBIL1	-0.859258692	6.192723437	-3.431662624	0.000825762	0.006405269	down
SPTY2D1-AS1	-0.608461405	4.715873042	-3.430372933	0.000829339	0.006428647	down
ACSM3	-0.606978593	5.950512124	-3.429445265	0.000831921	0.006444286	down
SEMA3E	-0.626071722	6.721865435	-3.429336539	0.000832224	0.006444448	down
PITX1	-0.535370642	7.983561602	-3.427580141	0.000837135	0.006475425	down
IQCH-AS1	-0.557533193	7.176133209	-3.425589977	0.000842732	0.006510365	down
ACAP3	-0.5738721	7.150715588	-3.41981269	0.000859181	0.006619517	down
LOC101929552	-0.772316571	5.341414001	-3.41338845	0.000877825	0.006733625	down
LOC101927869	1.041545089	4.26833327	3.412678203	0.000879909	0.006742818	up
DPF1	0.51488305	6.303657463	3.411162626	0.000884372	0.006767937	up
HCG11	-0.705002801	6.510678521	-3.408395641	0.000892576	0.00681701	down
HYAL1	0.706901726	6.414632066	3.407003414	0.00089673	0.006844163	up
BLOC1S1-RDH5	-0.784828859	6.138945165	-3.40652664	0.000898157	0.006850475	down
NAT6	-0.823377458	6.256128433	-3.405266427	0.000901939	0.006870142	down
TRIM72	0.717495523	5.421107943	3.401586106	0.00091307	0.006941033	up
FCER1G	0.825141693	9.476129082	3.39858275	0.000922248	0.006999158	up
CHP2	-1.410575829	9.878713165	-3.396840145	0.000927614	0.007032864	down
PNMA2	-0.856618118	6.302834184	-3.396598381	0.00092836	0.007036189	down
MYOZ3	-0.511692191	5.27067291	-3.393626758	0.000937584	0.007094324	down
CYP4F30P	-0.86560744	4.851627467	-3.393351389	0.000938443	0.007098472	down
LINC00862	0.927730008	4.16450713	3.391144796	0.000945354	0.007134641	up
ITK	-0.639562764	6.874047437	-3.390671	0.000946844	0.007143087	down
EREG	0.62804637	4.715155758	3.388234915	0.00095454	0.007191644	up
ERMAP	-0.599294145	7.416925911	-3.387958328	0.000955417	0.007195881	down
EVI2A	0.583046263	8.449522026	3.384866377	0.000965278	0.007253405	up
TSHR	0.582920328	3.977904782	3.381899825	0.000974829	0.007309316	up
DFNB59	-0.576721418	7.355522591	-3.376583453	0.000992167	0.007418323	down
PDYN	-0.604832044	4.073635611	-3.376505636	0.000992423	0.007418323	down
SGCE	-0.610022489	8.529309168	-3.376105757	0.000993739	0.007423301	down
ADH1C	-0.558598395	12.48104646	-3.375020305	0.00099732	0.007447612	down
1-Mar	0.551308808	5.868150268	3.374774741	0.000998132	0.007451237	up
CYAT1	-0.786456635	7.003079737	-3.370914276	0.001010974	0.007534795	down
CNTN4	-0.621279013	5.740840827	-3.369791373	0.001014739	0.007557918	down
FLT3LG	-0.582709622	5.35422886	-3.368616745	0.001018691	0.007579937	down
PPARG	0.924417109	6.590259989	3.361293384	0.001043657	0.007730445	up
FAHD2B	-0.877399335	4.594602747	-3.360099921	0.00104778	0.007755951	down
LY86	0.568681269	8.681635045	3.357426903	0.001057069	0.00781964	up
TRPC6	0.75674665	6.983432146	3.355218617	0.001064801	0.007864101	up
IGLC1	-0.655700567	6.818293781	-3.349300553	0.001085785	0.007983446	down
ASPM	0.509012351	5.188203971	3.348224025	0.001089643	0.00800872	up
GALNT15	0.52720538	5.211930508	3.347476256	0.001092331	0.008018152	up
PDXP	-0.539841519	8.105228681	-3.334350618	0.00114053	0.008323794	down
TFAP4	-0.718564449	6.299864527	-3.331632037	0.001150759	0.008387722	down
PRX	0.552509313	6.53533468	3.329264997	0.001159735	0.008447754	up
PLPPR1	0.671221191	5.186325749	3.327988223	0.001164604	0.008475109	up
PCAT19	0.916229512	8.515708503	3.327222075	0.001167535	0.008488827	up
SIAH3	-0.787702882	7.196946355	-3.32701434	0.00116833	0.008488827	down
ZNF684	-0.522996106	6.565375821	-3.326749032	0.001169348	0.00849069	down
TNS4	-0.609112373	7.064758004	-3.325414617	0.001174476	0.008514391	down

LINC00964	-0.583271138	7.0844878	-3.32135114	0.001190223	0.008605958	down
KIF12	-0.806928172	5.264454227	-3.321120219	0.001191124	0.008605958	down
DUSP27	0.749771357	4.632785752	3.320620316	0.001193076	0.00861096	up
BLK	-0.586251206	4.805399299	-3.320340258	0.001194171	0.008613418	down
CILP	-0.564537171	6.957646306	-3.319383995	0.001197917	0.008637709	down
EXO1	-0.762795849	5.409800135	-3.318958346	0.001199587	0.008645303	down
CLEC18A	-0.723679397	5.810558688	-3.31776458	0.001204285	0.008667211	down
CLEC18C	-0.723679397	5.810558688	-3.31776458	0.001204285	0.008667211	down
LOC101927020	0.696698566	5.837694115	3.31758961	0.001204975	0.008669444	up
RAC2	0.558657133	9.092289556	3.315611355	0.001212802	0.008717518	up
ASIC2	0.643197066	4.858672334	3.313697321	0.001220421	0.008761246	up
ARIH2OS	-0.713292051	6.151082028	-3.31022483	0.001234356	0.008836288	down
PPP4R4	-0.512117288	7.091519988	-3.30948909	0.001237328	0.008854785	down
FOXI1	0.704420526	6.101375501	3.307180098	0.001246697	0.008905036	up
GAPDHS	0.62392439	4.13316294	3.307054453	0.001247209	0.008905036	up
UGT2B28	-0.653793933	5.867495971	-3.305825505	0.001252225	0.008925195	down
HERC2P2	-0.504689692	10.11706521	-3.305462191	0.001253711	0.008925195	down
HERC2P9	-0.504689692	10.11706521	-3.305462191	0.001253711	0.008925195	down
MROH7	-0.656187963	7.388355706	-3.301579841	0.001269699	0.009024226	down
OR51E1	0.723764855	5.219658359	3.299416294	0.001278691	0.009065492	up
LOC101928429	0.848786641	5.168386017	3.298152777	0.001283969	0.009088879	up
GYPC	-0.753618672	8.889443095	-3.296362235	0.001291485	0.009139246	down
MYOM2	0.612103972	7.937275362	3.294727856	0.001298381	0.009173831	up
S1PR4	0.592488957	7.333612798	3.291121666	0.001313718	0.009267769	up
SERPINE1	0.549916165	5.437328651	3.287953046	0.001327334	0.009343918	up
CD200R1	-0.691710803	7.513345624	-3.287851482	0.001327773	0.009343918	down
EWSAT1	-0.813050836	4.802997496	-3.28644367	0.001333866	0.009383909	down
KRT12	0.817567116	3.320053233	3.28600011	0.001335792	0.00939456	up
ERC2-IT1	0.797657678	5.247025885	3.284784843	0.00134108	0.009423046	up
CD14	0.643451368	8.746251516	3.28438163	0.001342839	0.009432503	up
LILRB3	0.615652826	7.478898044	3.28357566	0.001346361	0.00945143	up
EPHA4	-0.560879057	7.403358411	-3.281476941	0.001355574	0.009498583	down
KCNJ2-AS1	-0.606093778	6.370416676	-3.279621542	0.001363767	0.009541356	down
ZNF366	0.613835936	5.112973737	3.278861403	0.001367137	0.009559078	up
TRY2P	-0.65522335	4.003288563	-3.27548139	0.001382217	0.009638431	down
FBXO17	-0.820861972	4.819767827	-3.274391438	0.001387113	0.009666193	down
LENG8	-0.581109115	9.588037953	-3.271208174	0.001401505	0.00974813	down
PSG7	0.745643325	5.218109365	3.268677637	0.001413044	0.009819928	up
HOPX	-0.579052219	7.263442225	-3.264316338	0.001433141	0.009922444	down
GPR1	0.980383742	5.926607877	3.263228322	0.001438196	0.00994328	up
SPATA31A1	0.763898628	4.320297603	3.259469675	0.001455788	0.010031494	up
SPATA31A3	0.763898628	4.320297603	3.259469675	0.001455788	0.010031494	up
SPATA31A5	0.763898628	4.320297603	3.259469675	0.001455788	0.010031494	up
SPATA31A6	0.763898628	4.320297603	3.259469675	0.001455788	0.010031494	up
SPATA31A7	0.763898628	4.320297603	3.259469675	0.001455788	0.010031494	up
PARTICL	-0.508861637	6.345013437	-3.25927666	0.001456697	0.010034728	down
AMELY	0.801666626	5.318599795	3.258600683	0.001459883	0.010053648	up
LOC101927690	0.817476223	4.691614721	3.25822122	0.001461675	0.01005992	up
FCGR1B	0.735480701	8.01921018	3.257977092	0.001462829	0.010064827	up
SULT1E1	-1.009322566	6.584774997	-3.255968555	0.001472354	0.010124265	down
OSGIN1	0.694969251	6.287396616	3.25555967	0.0014743	0.010132085	up
CASP5	0.721842706	5.856011199	3.25550705	0.001474551	0.010132085	up
IP6K3	0.536507978	7.531709145	3.252421698	0.001489316	0.01020403	up
LAPTM5	0.631011131	11.09808186	3.251695899	0.00149281	0.010218767	up
AATK	0.530022364	6.921579162	3.248197923	0.001509754	0.010316198	up
AHCTF1P1	-0.842970425	6.332320409	-3.247012033	0.001515539	0.010346439	down
MS4A14	0.824129302	5.423904495	3.246033833	0.001520326	0.01037292	up
HSD17B3	-0.521961888	7.688919807	-3.242619948	0.001537144	0.010472025	down
CSF1R	0.516780234	8.077154151	3.240622999	0.001547063	0.010527032	up
LL22NC03-75H12.2	-0.603092028	6.775411652	-3.237126355	0.001564573	0.010617709	down
CELF2-AS1	-0.830972655	4.691668843	-3.234118931	0.001579781	0.010701835	down

CYP3A5	0.548046701	6.465369455	3.233097124	0.00158498	0.010730684	up
WFDC12	-0.804124335	4.79116069	-3.231990566	0.001590627	0.01075967	down
CDKAL1	-0.51840594	6.280346546	-3.231981318	0.001590675	0.01075967	down
DUSP1	-0.510289051	11.88431539	-3.229064918	0.00160565	0.010841696	down
FAM19A2	-0.618404774	6.463683106	-3.228705971	0.001607502	0.010844583	down
LOC100130278	0.661097404	5.361088068	3.228155154	0.001610348	0.010856404	up
COX3	0.510520566	12.07078741	3.227066434	0.001615987	0.01087331	up
LOC101929612	0.510520566	12.07078741	3.227066434	0.001615987	0.01087331	up
LOC105376072	0.616272928	5.722156393	3.226972951	0.001616472	0.01087331	up
CSTA	0.572842163	11.48347996	3.226816493	0.001617284	0.010875238	up
LY6G6D	0.505047933	5.631500435	3.220739467	0.001649125	0.011053543	up
LY6G6F	0.505047933	5.631500435	3.220739467	0.001649125	0.011053543	up
PVRL3-AS1	-0.806007059	4.620060893	-3.220237051	0.001651783	0.011068112	down
NKX2-8	0.685256729	5.669952782	3.220005737	0.001653009	0.011073074	up
CSF2RB	0.733571417	8.17691827	3.218639586	0.001660263	0.011107961	up
BCORP1	0.848008149	4.776142785	3.218566979	0.001660649	0.011107961	up
SLC26A9	0.608742975	6.582852643	3.21600467	0.001674337	0.011192962	up
LOC101927719	0.574521363	7.508305946	3.213992147	0.001685162	0.01124886	up
C2CD4A	0.768820405	5.549245189	3.210874625	0.001702058	0.011338449	up
CEMP1	-0.526240102	5.868994925	-3.209007574	0.001712253	0.011393068	down
ARHGAP20	-0.549405321	6.28022399	-3.207419636	0.001720968	0.011441058	down
PCDHB6	-0.508020258	5.136788631	-3.207240342	0.001721955	0.011444286	down
TLR6	0.504683636	5.586256741	3.207045046	0.00172303	0.011448101	up
ATXN8OS	0.820595147	4.678942814	3.203378398	0.001743335	0.011569158	up
EPHA7	0.59812468	5.247118034	3.202180829	0.001750015	0.011607131	up
LOC101928818	0.518104679	4.174473764	3.201453112	0.001754086	0.011627377	up
PWRN1	0.518104679	4.174473764	3.201453112	0.001754086	0.011627377	up
THEMIS2	0.563901265	7.64273151	3.201004638	0.001756599	0.011634149	up
HADHAP1	-0.550829599	7.252536022	-3.198913938	0.001768359	0.01168468	down
ENAM	0.616043519	4.194962975	3.198543777	0.001770449	0.011688343	up
BICC1	0.508779362	8.564271731	3.198260011	0.001772053	0.011695548	up
ENG	0.676116031	5.780365098	3.194754918	0.001791972	0.01179633	up
CLEC4A	0.536696477	6.912664513	3.193500925	0.001799149	0.01183334	up
CASKIN2	-0.513352405	6.643315913	-3.192776268	0.001803308	0.011850458	down
FZD9	-0.707602244	4.450159883	-3.192161579	0.001806843	0.011866861	down
TOP3B	-0.512967394	6.51230916	-3.191982281	0.001807876	0.011870228	down
ARHGEF33	-0.566037745	6.333882235	-3.190361161	0.001817235	0.011912575	down
SLITRK5	0.549065267	5.482352288	3.187740829	0.001832459	0.011997145	up
DPY19L2P3	-0.775406135	4.421004283	-3.186657401	0.001838789	0.012018091	down
EID3	-0.523262282	6.727031531	-3.180943081	0.001872509	0.012203371	down
LOC100506860	0.545889336	4.799339554	3.180743138	0.001873699	0.012204162	up
IGK	-0.819063991	7.764426878	-3.176848888	0.001897021	0.012320928	down
FAT2	-0.808787464	7.856917521	-3.17384949	0.001915168	0.0124046	down
UPK1B	0.809889323	10.63061838	3.171166991	0.001931535	0.012484722	up
RNU6-501P	0.867644025	4.649183076	3.169650219	0.001940846	0.012529148	up
ZBTB20-AS1	-0.580216354	4.707988768	-3.169600647	0.001941151	0.012529148	down
FLJ20712	-0.618993282	4.89676708	-3.16937613	0.001942533	0.012534526	down
PF4	-0.796730085	6.745813965	-3.168924862	0.001945314	0.012545378	down
LRRC2-AS1	-0.776737031	5.137946409	-3.168362662	0.001948783	0.012560658	down
CD1A	0.583094947	6.255996971	3.167200501	0.001955973	0.012596334	up
GCSAML	0.780941013	4.85777035	3.16643976	0.001960693	0.01260895	up
LPAR5	-0.50871548	6.132713538	-3.165058353	0.001969291	0.01265355	down
HSD17B6	0.660977025	6.224503924	3.160668753	0.001996844	0.012794592	up
KCNN3	0.644350577	7.748879253	3.158721349	0.002009182	0.012860798	up
MGC12488	0.518811709	6.810747883	3.150241951	0.002063735	0.013142068	up
LOC100289058	0.521271582	6.438391088	3.148803803	0.002073123	0.013187141	up
SFTPA1	1.733798977	7.539272146	3.147248225	0.002083322	0.013228429	up
SFTPA2	1.733798977	7.539272146	3.147248225	0.002083322	0.013228429	up
IFI6	-0.605225258	9.406003312	-3.144731788	0.00209992	0.01332049	down
COX6A2	0.552138247	6.141946051	3.144031587	0.00210456	0.013342511	up
GPR82	0.556723636	5.016666342	3.140708989	0.002126708	0.013471707	up

LINC00113	-0.670070655	5.902035881	-3.140575701	0.002127601	0.013473626	down
LOC102724888	0.731974716	4.051880055	3.138517028	0.002141438	0.013547704	up
LOC102725420	0.731974716	4.051880055	3.138517028	0.002141438	0.013547704	up
ID1	-0.576534924	10.2718291	-3.137757953	0.002146561	0.013574871	down
CYS1	-0.815661747	5.891944087	-3.137098271	0.002151022	0.013599319	down
NXPH3	-0.567487657	5.971949954	-3.131980174	0.002185929	0.01378566	down
ZNF711	-0.533513162	6.682724092	-3.123517708	0.002244801	0.014079175	down
PCSK1N	0.634384842	7.850904515	3.123062125	0.002248012	0.014091572	up
GPR146	-0.747283592	5.454544116	-3.121659669	0.002257923	0.014149813	down
C14orf132	-0.523863613	9.951167932	-3.11928931	0.002274765	0.014228031	down
MYC	-0.508612501	9.074153401	-3.119093573	0.002276161	0.014228968	down
AQP10	0.740504437	5.718002172	3.116298859	0.002296181	0.014326661	up
FAM46B	-0.511313562	6.941908074	-3.115625134	0.002301031	0.014353003	down
ALAS2	-0.896951137	6.042650468	-3.113658649	0.002315243	0.014421955	down
LINC00544	-0.694354499	3.95513623	-3.113537254	0.002316123	0.014423502	down
PGLYRP4	-0.551830916	6.986381119	-3.111973429	0.002327487	0.014478477	down
FERMT3	0.736359674	6.980169733	3.110892384	0.002335373	0.014514432	up
C10orf71-AS1	0.785090362	4.910134716	3.11083266	0.002335809	0.014514432	up
LOC101928739	0.788645342	3.878650179	3.110247095	0.002340092	0.01453264	up
C17orf50	-0.736183981	6.181602505	-3.106590147	0.002367002	0.014660393	down
SIX2	-0.545906387	9.410090969	-3.106281278	0.002369288	0.014666595	down
C1orf162	0.690358821	8.095828522	3.10393802	0.002386698	0.014752544	up
LOC105373383	-0.502468819	7.048030074	-3.103746679	0.002388125	0.0147552	down
C3AR1	0.592289705	8.020093737	3.100682715	0.002411081	0.014876909	up
DHRS2	-0.501802647	4.710087214	-3.098328965	0.002428854	0.01495022	down
ECHDC3	-0.575632811	8.432197274	-3.097457857	0.002435463	0.01498282	down
RGMB-AS1	0.509603537	6.617667339	3.096905513	0.002439662	0.01500057	up
GMFG	0.64531287	8.168322832	3.095081481	0.002453576	0.015069892	up
SSC5D	0.622067643	6.949882631	3.093168291	0.002468249	0.015143722	up
IGLV1-44	-0.617186175	6.470153446	-3.091968745	0.00247749	0.015188179	down
TIFAB	0.556854768	5.064865136	3.091305054	0.002482617	0.015215525	up
CCNJL	0.631472406	5.939979038	3.090431075	0.002489383	0.015244719	up
IGKC	-0.729041377	7.402003719	-3.089038647	0.002500199	0.015302744	down
HTR2B	0.892617135	5.166966911	3.087858577	0.002509399	0.015354938	up
OR8D2	0.676657687	4.419564988	3.087555301	0.002511768	0.015362807	up
DDC	0.56238518	5.693657645	3.087293396	0.002513816	0.015362807	up
LINC01619	-0.870782669	4.40863219	-3.087263678	0.002514048	0.015362807	down
LOC101928554	0.77718895	4.123961794	3.087165433	0.002514817	0.015363169	up
GPR143	-0.734487273	5.765334115	-3.085812695	0.002525423	0.015401407	down
DNASE1L2	-0.611477264	5.93030804	-3.077657509	0.002590245	0.015723239	down
NEIL2	-0.590645586	7.314751176	-3.075583033	0.002606978	0.015812202	down
STMN2	0.56207509	4.184761218	3.075307073	0.002609211	0.015813079	up
LOC105373878	-0.827909325	6.976318661	-3.075275015	0.002609471	0.015813079	down
TGIF2LY	0.72012532	4.967082088	3.075222975	0.002609892	0.015813079	up
NPIPB8	-0.552789067	7.725376697	-3.074083293	0.002619137	0.015852269	down
LOC105373150	-0.733206947	4.413500661	-3.072841962	0.002629241	0.015882866	down
LOC100653149	0.689083243	5.72196054	3.072704604	0.002630361	0.015882866	up
SERPINH1	-0.902497923	7.422432364	-3.072546334	0.002631653	0.015885915	down
LINC01416	0.718566391	5.321716623	3.067297789	0.00267481	0.016095381	up
LOC102723845	-0.58997582	6.180544647	-3.062317346	0.002716368	0.016279595	down
ANO1	-0.531033153	5.760518201	-3.062163556	0.002717661	0.016279595	down
CELA1	0.59193875	5.223571956	3.060629242	0.002730589	0.01631495	up
TLR7	0.516265754	5.764671865	3.059602911	0.002739269	0.016345427	up
ASB16-AS1	-0.603316049	6.875131403	-3.058681744	0.002747081	0.016371829	down
LOC652993	0.642282511	5.566187727	3.056718811	0.002763797	0.016440235	up
FAM24B	0.513852551	6.423749132	3.055954741	0.002770329	0.016466222	up
IGHA2	-0.512012932	6.037585518	-3.055007662	0.002778445	0.016505871	down
EGOT	-0.695526469	5.028330251	-3.054465989	0.002783097	0.016525106	down
C2orf61	-0.674454525	3.884823548	-3.054225952	0.002785161	0.016528568	down
IGFBP4	-0.52725645	8.80686585	-3.052116002	0.002803362	0.016619309	down
CYP11A1	-0.775787325	4.579175974	-3.051502031	0.002808679	0.01664219	down

LINC00528	0.62458846	4.311459589	3.051412414	0.002809456	0.016642475	up
CYP27B1	0.617083621	5.789510863	3.046703412	0.002850558	0.016827481	up
CREG2	0.611350893	4.075686137	3.046025839	0.002856518	0.016855671	up
PRG3	-0.657369917	4.292807631	-3.043484608	0.002878973	0.016953125	down
ZNF876P	0.841747213	3.296481382	3.041857445	0.002893436	0.017016354	up
GAS1	-0.522460237	6.207500368	-3.040655023	0.002904167	0.017062608	down
LINC00189	0.612946228	4.159038231	3.038901397	0.002919883	0.017132182	up
PKIA	-0.583241161	6.917247298	-3.038303066	0.002925263	0.017154935	down
LINC00163	-0.726456881	5.213404128	-3.036892245	0.002937986	0.017185488	down
GPNMB	0.785638998	9.642652501	3.035678691	0.002948971	0.017232009	up
LOC441052	-0.675997999	5.152137475	-3.03554244	0.002950206	0.01723482	down
CTCFL	0.785569373	4.196196666	3.031280386	0.002989101	0.017399722	up
LOC100507277	0.69255157	4.982763169	3.030240249	0.002998665	0.017439493	up
FGL2	0.558215397	8.219202359	3.030231457	0.002998746	0.017439493	up
KIAA1211	0.639438471	7.302310691	3.029912745	0.003001682	0.017446273	up
ABHD8	-0.679780167	6.74433632	-3.029643716	0.003004162	0.017456247	down
FSTL3	-0.633546498	6.010122325	-3.028044582	0.003018946	0.01752877	down
MIR8071-1	-0.975380833	6.985155843	-3.027801406	0.0030212	0.01753116	down
MIR8071-2	-0.975380833	6.985155843	-3.027801406	0.0030212	0.01753116	down
TMEM72	0.578674942	5.863843907	3.023846194	0.00305808	0.017716354	up
C2orf82	-0.633054363	5.379243384	-3.023466656	0.00306164	0.017727084	down
FSHR	0.521214948	3.54864614	3.021985725	0.003075571	0.017789699	up
LINC00410	0.660295319	4.719460993	3.021161283	0.003083351	0.017816651	up
MSR1	0.668044805	6.633584221	3.020043054	0.003093933	0.017855207	up
EDNRA	-0.64426071	6.673602458	-3.016016081	0.003132319	0.018017542	down
ZNF835	-0.632414563	3.658978324	-3.012770724	0.003163574	0.018165296	down
LOC101927701	0.745166852	4.56863744	3.012045081	0.003170602	0.018191927	up
SMOC1	0.656920235	6.391941021	3.009421833	0.003196128	0.018310783	up
TIMD4	-0.776189082	4.469430248	-3.007414861	0.003215785	0.018399099	down
KRT13	1.223843739	5.606529356	3.005050962	0.003239081	0.018504199	up
LINC00174	-0.776328382	6.013633759	-3.004563229	0.003243907	0.018519467	down
PSD2	-0.611091586	5.37231113	-3.002464625	0.003264748	0.018617716	down
ABCD2	-0.635662187	6.718396503	-3.001007458	0.003279291	0.018678779	down
ERAP2	-0.63838941	7.235092514	-2.996145887	0.003328245	0.01889702	down
RTKN2	0.610713569	4.440853302	2.996046486	0.003329253	0.018898044	up
DCAF12L2	-0.813786496	4.795529438	-2.995792029	0.003331835	0.018902133	down
GCNT3	0.527488043	8.060462435	2.992884197	0.003361466	0.019047753	up
LINC00893	-0.509032635	7.789550001	-2.992161839	0.003368865	0.019079976	down
HTR1F	-0.726407029	5.471656811	-2.992031327	0.003370203	0.019079976	down
HBA1	-0.663765407	14.9036553	-2.986399926	0.00342842	0.019337534	down
HBA2	-0.663765407	14.9036553	-2.986399926	0.00342842	0.019337534	down
LOC100287525	-0.603504219	6.362908997	-2.986373519	0.003428695	0.019337534	down
NT5M	-0.678358089	4.562669986	-2.98609035	0.003431647	0.019339853	down
FAM150B	0.786152494	3.891657149	2.98439223	0.003449398	0.019425282	up
EVC2	-0.512130505	5.529058837	-2.984260586	0.003450778	0.019425282	down
DUSP2	-0.976224537	7.300659795	-2.98170571	0.003477655	0.019536462	down
LINC00173	-0.53685191	5.135891509	-2.981068808	0.003484385	0.019569456	down
GDF15	0.555211823	8.257965825	2.980351791	0.003491976	0.019598481	up
GZMB	0.807110167	7.255488327	2.98031646	0.00349235	0.019598481	up
SERPINA2	0.649488187	4.085385478	2.978125123	0.003515646	0.019706259	up
LOC100288152	-0.796472515	8.477333477	-2.977591901	0.003521337	0.019723631	down
LRRC75A	-0.602348128	3.902779401	-2.975433792	0.003544454	0.019814236	down
C3orf56	0.774120488	4.682277966	2.97378699	0.003562188	0.019893894	up
LOC100270804	-0.527126236	7.184828737	-2.973493306	0.00356536	0.019906736	down
ACTA1	0.652218056	4.880778889	2.971632348	0.003585515	0.019980453	up
SLC35F1	-0.588201781	6.154663588	-2.96783005	0.003627023	0.020157385	down
LOC105379426	0.51218185	8.264461393	2.963250139	0.003677607	0.020379325	up
MAGI1-IT1	-0.736961348	4.916083738	-2.962670557	0.003684054	0.020409769	down
IGHG2	-0.681672524	6.43518693	-2.961065216	0.003701967	0.02049409	down
PPM1J	-0.597568899	6.282862545	-2.958400902	0.003731873	0.020623502	down
LY6E	-0.502612351	10.81880511	-2.958259048	0.003733472	0.020623502	down

LOC646626	-0.717247812	4.816410884	-2.956375067	0.003754761	0.020716048	down
LOC101928847	0.783743597	3.936954616	2.9506061	0.003820649	0.02100345	up
C17orf47	-0.578307944	4.056743204	-2.947793586	0.003853155	0.021136354	down
HRH4	0.577444601	4.563506349	2.946680868	0.003866085	0.021181843	up
HK3	0.913728625	4.89374725	2.943118057	0.003907756	0.021369139	up
SLC22A3	0.517567327	8.576858728	2.941689187	0.003924584	0.021450888	up
NPVF	0.776813751	4.055089489	2.941520437	0.003926576	0.021456639	up
GAPT	0.679869167	6.016745433	2.941324977	0.003928884	0.021458982	up
CHGA	0.566438385	6.481487836	2.94031489	0.003940833	0.02151395	up
BMS1P21	0.682866888	5.576024975	2.938260966	0.003965231	0.021626468	up
MBL1P	0.682866888	5.576024975	2.938260966	0.003965231	0.021626468	up
TMPO-AS1	-0.645760054	5.416777291	-2.937933119	0.003969139	0.021642609	down
LRRC66	0.611748125	5.46529006	2.932246618	0.004037477	0.021904218	up
PTN	-0.581764621	8.549901113	-2.929365513	0.004072512	0.022034937	down
CLIC2	0.607186952	6.048309619	2.929331163	0.004072932	0.022034937	up
APBB1IP	0.504666151	7.41879798	2.926904456	0.004102661	0.022170558	up
LINC01362	0.584090772	5.302576695	2.925208675	0.004123554	0.022250821	up
LOXL3	0.636359088	6.034856097	2.922891205	0.004152264	0.022379304	up
LOC100288069	-0.607029832	6.497697315	-2.921835614	0.004165403	0.022413085	down
LOC100996442	-0.607029832	6.497697315	-2.921835614	0.004165403	0.022413085	down
LOC101928344	-0.607029832	6.497697315	-2.921835614	0.004165403	0.022413085	down
LOC101928670	-0.607029832	6.497697315	-2.921835614	0.004165403	0.022413085	down
LOC101929540	-0.607029832	6.497697315	-2.921835614	0.004165403	0.022413085	down
ERP27	-0.602360831	8.610759986	-2.918193742	0.004211024	0.022615801	down
MAGEA6	0.512665326	4.113596934	2.91481453	0.004253766	0.02281865	up
LOC100507534	-0.657156478	3.615473294	-2.912661264	0.004281208	0.02291741	down
LINC01563	0.640722072	5.333312297	2.911943973	0.004290386	0.022950399	up
DNM1	-0.727835795	5.422645257	-2.910934368	0.004303334	0.023008884	down
ASRGL1	0.615882099	8.050029259	2.909057926	0.004327496	0.023100208	up
LOC102724009	-0.570820098	3.790466569	-2.908271141	0.004337663	0.023138257	down
DPP4	-0.501814605	8.148033444	-2.908181776	0.004338819	0.023139019	down
LOC101927494	0.688100852	3.063403241	2.903592443	0.004398581	0.023397621	up
SERPINA1	0.569003449	10.2745817	2.902835894	0.004408505	0.023434435	up
HOXA1	0.942629452	6.768655612	2.902830154	0.00440858	0.023434435	up
CLCA4	-0.924848049	8.115197273	-2.901353155	0.004428014	0.023515842	down
LOC101927787	0.646498625	5.123195369	2.900418591	0.004440351	0.023570397	up
NPHP3-AS1	0.503439767	4.914763738	2.896447938	0.00449312	0.023795195	up
RRS1-AS1	-0.767024954	4.721806316	-2.89382548	0.004528286	0.023964762	down
DOCK2	0.53683184	6.088410114	2.890765218	0.004569643	0.02415564	up
LAIR1	0.505909934	7.312946886	2.890138568	0.004578154	0.024195031	up
C1QA	0.703092813	9.27568769	2.889190136	0.004591063	0.024246421	up
INSL4	-0.550581841	5.05522593	-2.887804039	0.00460999	0.024318257	down
PGLYRP3	0.637841868	4.078092002	2.887318878	0.004616632	0.024336426	up
LOC221272	-0.518084832	6.792664039	-2.886614235	0.004626293	0.024370481	down
IL4	0.546946534	4.337120773	2.884975973	0.004648828	0.024466917	up
OR51B6	0.633251367	5.311938604	2.883936205	0.004663183	0.024536507	up
ADORA3	0.523712412	5.100724078	2.88365574	0.004667062	0.024545605	up
LHB	0.670773544	6.770551529	2.882909368	0.004677399	0.024582986	up
LINC00473	-0.703683363	5.306536739	-2.88236604	0.004684937	0.02461324	down
MRGPRG-AS1	-0.530938588	6.474435017	-2.88181092	0.00469265	0.024634793	down
LRRTM1	0.709464487	4.800252244	2.878212577	0.00474293	0.024875874	up
NOL4	0.553471175	4.37419195	2.878128864	0.004744106	0.024876326	up
OXER1	-0.649828408	6.852842765	-2.877252151	0.004756434	0.024928482	down
CA2	0.81624097	6.298039582	2.876796149	0.004762857	0.024938323	up
BTBD11	0.555700094	6.3876962	2.874098077	0.004801028	0.025105627	up
SHISA7	0.523384214	5.109606071	2.870151827	0.004857362	0.025326205	up
CCDC141	0.607330419	4.319779846	2.869228109	0.004870635	0.025382434	up
NLRC4	0.544799308	5.496535794	2.868727716	0.00487784	0.025414179	up
CACNG4	0.634185845	7.693990388	2.865740329	0.004921054	0.025580965	up
CAMP	-0.709509313	6.178704028	-2.865526079	0.004924166	0.02559132	down
OR2W1	0.716337623	3.993392656	2.865361835	0.004926554	0.025597902	up

KRT15	-0.870021847	10.55524677	-2.864671937	0.004936593	0.025644232	down
SLC17A6	0.705470212	4.15138365	2.86222758	0.004972316	0.025788735	up
LINC01149	0.583924696	3.50231332	2.856548542	0.005056226	0.026140813	up
LOC105379499	-0.758387783	7.479484685	-2.853627895	0.005099883	0.0263248	down
LOC101928702	-0.754259544	5.030199012	-2.853545007	0.005101127	0.026325271	down
LINC01087	0.813044813	3.651075442	2.853463849	0.005102345	0.026325609	up
SHROOM4	-0.548865816	5.106840731	-2.853367642	0.005103789	0.026327114	down
GAFA3	0.734646837	3.275441636	2.848911072	0.005171117	0.026602303	up
LOC101927382	-0.818818625	5.489417375	-2.848303832	0.005180353	0.026643817	down
OLR1	0.712731185	8.143491073	2.847559008	0.005191703	0.026688624	up
MILR1	0.724631618	4.586783002	2.845952028	0.005216268	0.026792331	up
ZPLD1	0.597172309	4.012491952	2.845236514	0.00522724	0.026830582	up
EEF1A2	0.708046343	5.300534893	2.843607555	0.005252298	0.026919064	up
LOC101927363	0.635575983	3.423406032	2.838863095	0.005325908	0.027223064	up
LINC00319	-0.696942364	4.311607788	-2.838834446	0.005326355	0.027223064	down
CA10	0.54278564	4.020983169	2.837389968	0.005348955	0.027301721	up
PIWIL1	0.71257683	4.88422426	2.837089107	0.005353673	0.027301721	up
LINC01153	-0.706573285	3.335089225	-2.835594274	0.005377171	0.027403053	down
LOC101929312	0.676366209	3.864892934	2.833207486	0.005414886	0.027582945	up
ISL1	-0.70436635	8.341080296	-2.831822116	0.005436888	0.027664167	down
USP44	0.664757817	5.120406965	2.830750149	0.005453969	0.027732539	up
COX7B2	-0.694322899	4.801785812	-2.830465514	0.005458512	0.027749464	down
LOC101928271	0.766010598	3.259597588	2.828544823	0.005489263	0.027887164	up
HMGCS2	0.564116332	6.277999126	2.825700019	0.005535099	0.028082542	up
CD300LF	0.604165438	7.851105787	2.823120637	0.005576961	0.028282362	up
REN	-0.604407576	5.191592742	-2.822364975	0.005589279	0.028322667	down
RPL13AP17	-0.655641923	7.515296333	-2.821935444	0.005596293	0.028348921	down
SYT2	-0.737781774	5.901934551	-2.821643513	0.005601064	0.028360507	down
GPR83	0.652556105	5.066418895	2.821211605	0.005608129	0.028389989	up
EPO	0.538766816	5.272497271	2.817459244	0.005669858	0.028619998	up
ZSCAN4	-0.592628207	5.435977111	-2.816079514	0.005692711	0.028709969	down
LOC339539	-0.604270596	5.017102272	-2.81461208	0.005717109	0.028811477	down
MAS1	-0.696271451	4.516755816	-2.814430869	0.005720128	0.028816423	down
NAPSA	0.759726339	6.921916022	2.811842177	0.005763422	0.028997611	up
OR13C4	-0.564585444	5.565701965	-2.811319023	0.005772208	0.029021166	down
P2RY14	0.667447841	6.434453891	2.810523623	0.005785589	0.029082039	up
CASC23	-0.626287874	5.060257995	-2.81044674	0.005786884	0.029082147	down
LOXHD1	0.648020965	3.874424167	2.808340568	0.00582246	0.029209512	up
HRES1	0.698079048	5.202777228	2.807054956	0.005844274	0.029267543	up
SPINK1	0.713324095	5.1881991	2.805008958	0.005879144	0.029403445	up
SAGE1	0.714565544	3.642838846	2.802108489	0.005928902	0.029639318	up
TAS2R40	0.738226206	4.507728375	2.800341765	0.005959398	0.029765709	up
CD72	-0.555522574	5.882590973	-2.799867657	0.005967607	0.029800189	down
KIAA0226L	0.515903416	5.904009593	2.797961541	0.006000711	0.029923584	up
LOC441179	-0.636583872	5.871155162	-2.79795885	0.006000758	0.029923584	down
LOC105370888	-0.624536955	5.0622134	-2.797916844	0.006001489	0.029923584	down
CENPT	-0.650745366	5.536234794	-2.796815013	0.006020704	0.029986652	down
CRTAM	0.854770548	5.001902944	2.794306134	0.006064666	0.030172704	up
DKFZP586B0319	0.545045695	6.979927687	2.794146028	0.006067481	0.030180135	up
NR1I2	-0.506520153	5.490425565	-2.793565796	0.006077694	0.030211194	down
LOC101929897	-0.631064713	5.766394144	-2.791872795	0.006107583	0.030339956	down
LOC101927914	-0.685196708	4.214007033	-2.789851516	0.006143444	0.030491563	down
APOA4	0.689845921	5.715349288	2.789723316	0.006145725	0.030496256	up
MIR5188	0.599060569	5.202212655	2.787418056	0.006186871	0.030653807	up
CLUL1	-0.730163906	5.998723327	-2.787090131	0.006192745	0.030676253	down
C5AR2	0.687837016	4.208422702	2.784157064	0.006245506	0.030837271	up
TREML3P	0.558159846	4.537311198	2.782774502	0.006270518	0.030920655	up
PRAC1	0.7827868	4.668907002	2.778446945	0.006349399	0.031252006	up
AURKC	-0.601829232	6.709985229	-2.77784208	0.006360496	0.031296767	down
C6orf201	0.804325989	5.006574873	2.777511691	0.006366565	0.031313134	up
PLIN1	-0.559783934	4.411006753	-2.776276617	0.006389299	0.031411416	down

OLFM2	-0.528932368	5.451371947	-2.773487848	0.006440903	0.031631067	down
DAPK1-IT1	-0.510469724	6.711665102	-2.772625359	0.00645694	0.031696189	down
TCEAL2	-0.788477349	5.877249263	-2.772542372	0.006458485	0.031696959	down
UBBP2	0.623584777	7.171622198	2.771904905	0.006470363	0.031748434	up
HMGB4	0.653869582	4.36067623	2.769377645	0.006517652	0.031939293	up
CABS1	0.546626123	2.520810233	2.765672237	0.006587555	0.032219622	up
LOC101927417	0.660185814	4.114725027	2.765083126	0.006598732	0.032267375	up
SSMEM1	0.513588851	4.463193138	2.76031175	0.006689891	0.032573634	up
EHMT1-IT1	-0.611151554	5.102630697	-2.756762665	0.006758439	0.032865357	down
NOS3	0.620929027	4.864984787	2.751338055	0.006864448	0.033253402	up
NDNF	0.760641578	10.31754702	2.748493358	0.006920642	0.033440497	up
FLJ11710	-0.547260756	6.407424251	-2.74753067	0.006939754	0.033492253	down
LOC101559451	-0.595700009	5.769299032	-2.744674787	0.006996733	0.033743904	down
C20orf78	-0.67084312	5.310052185	-2.742637294	0.007037643	0.033891103	down
HFM1	0.559342084	4.124307921	2.741201486	0.007066603	0.034009049	up
MFAP4	-0.565211279	3.934026976	-2.740651562	0.007077723	0.034037536	down
LOC101929064	-0.638066559	5.091656285	-2.740613963	0.007078484	0.034037536	down
SLIT2-IT1	-0.70403899	4.619080823	-2.739712289	0.007096754	0.034111022	down
TPSB2	0.84620747	9.848104605	2.739444365	0.007102191	0.034118007	up
LOC105378470	0.552384024	4.570463365	2.737718358	0.007137307	0.034226699	up
KRTAP3-2	-0.668314533	4.536566648	-2.735246843	0.007187866	0.03440413	down
TDRD12	-0.642212001	4.080502415	-2.73481874	0.007196656	0.034424559	down
LOC105370737	0.827147536	3.625199679	2.733870085	0.007216171	0.034489006	up
IL1R2	0.740006974	6.777106161	2.732127242	0.007252147	0.034617481	up
HBB	-0.519652059	14.70775889	-2.731867608	0.00725752	0.034632133	down
LOC101927534	0.531750327	6.274841493	2.731282501	0.007269643	0.034675094	up
MS4A2	0.649426967	6.441400709	2.731010805	0.007275278	0.034679435	up
RAX2	0.53401225	7.333066186	2.729744775	0.00730159	0.034766288	up
PRKAR1AP1	-0.646556278	5.342685468	-2.726078475	0.007378276	0.03507292	down
LINC00993	0.521262723	5.592352345	2.724122243	0.007419492	0.035217525	up
LOC100996263	0.526411233	3.835621221	2.718824593	0.00753216	0.035596937	up
LOC147791	-0.519427859	5.556279617	-2.71789312	0.00755213	0.035654421	down
ADGRF5	0.754791492	9.623696542	2.71721572	0.007566683	0.035715744	up
COL6A5	0.505131767	4.318813942	2.715603617	0.007601419	0.035864878	up
CLMP	0.525353323	4.390850712	2.714258321	0.007630517	0.035972439	up
FLJ21369	0.569066373	5.407663593	2.712513839	0.0076684	0.036106307	up
LOC100507336	0.698648957	6.900764209	2.711891976	0.007681946	0.036155176	up
AGER	-0.651952613	5.571281255	-2.71147489	0.007691043	0.036190533	down
POU4F3	0.611966671	3.35090087	2.709813751	0.007727372	0.036326691	up
CCNT2-AS1	-0.687835622	4.194617975	-2.701893498	0.007902736	0.036996079	down
SYNDIG1	0.550649817	5.14761285	2.701498109	0.007911584	0.037014738	up
LOC101927069	0.620936628	3.10114476	2.701398347	0.007913818	0.037017605	up
LOC152225	0.626952298	5.139905711	2.698508678	0.007978775	0.03726801	up
ADGRE1	-0.670927529	6.268012687	-2.697323543	0.008005555	0.037362147	down
LOC79999	-0.58827254	5.312398268	-2.694246668	0.008075463	0.037581266	down
CLSTN3	-0.601145066	6.161386759	-2.690655265	0.008157761	0.037856255	down
ERVMER61-1	0.701620426	3.065059419	2.688710397	0.008202645	0.03801287	up
KIR2DL1	-0.532802827	4.575305637	-2.688688014	0.008203163	0.03801287	down
SATB2-AS1	0.560022924	4.390737203	2.681962863	0.008360105	0.038620787	up
CCL22	0.725310792	5.246223102	2.681492684	0.008371179	0.038642732	up
GTF2IP12	-0.517259037	7.584117212	-2.681221024	0.008377583	0.038664204	down
SERPINB2	0.795838338	8.649327088	2.677965275	0.00845468	0.038957035	up
LOC283335	-0.717817496	5.582165681	-2.677831387	0.008457864	0.038963845	down
GRP	0.839271692	7.717220254	2.676453493	0.008490697	0.039083181	up
LOC100506446	-0.541529138	5.894638616	-2.673692765	0.008556828	0.03931916	down
FPR1	0.643050158	7.819451991	2.673202102	0.00856863	0.039347123	up
B3GALT5	-0.604180033	6.006814462	-2.672873132	0.008576551	0.039367664	down
MGAT4D	0.576562017	5.221542965	2.672702288	0.008580668	0.039370731	up
EVI2B	0.582739499	8.552493814	2.670282133	0.008639171	0.039527973	up
CXorf67	0.532172986	3.970050234	2.666853581	0.008722669	0.039832754	up
ARL5C	-0.626106365	5.535672145	-2.666667607	0.008727219	0.039835054	down

LOC101929154	0.62941712	4.763215917	2.665438732	0.008757338	0.039948577	up
LOC101928389	-0.621731321	3.406018492	-2.663696988	0.008800189	0.040087993	down
TPH2	-0.687708187	3.64690537	-2.663194156	0.008812595	0.040128497	down
MMP7	-0.634676057	8.258324255	-2.66202375	0.008841532	0.040228416	down
PP2D1	0.728848579	4.331097804	2.66098011	0.008867408	0.040321812	up
FAM101A	-0.624977713	5.41235906	-2.657464796	0.008955072	0.04059917	down
ZNF664-FAM101A	-0.624977713	5.41235906	-2.657464796	0.008955072	0.04059917	down
HCK	0.503015057	8.371863572	2.654891791	0.009019732	0.040851766	up
PTPRCAP	-0.604376181	6.852143558	-2.654700516	0.009024556	0.040865508	down
FLJ27354	-0.586929657	3.965863976	-2.650193128	0.009138901	0.041293223	down
MMRN1	0.745260578	3.717611551	2.650093958	0.009141432	0.041296485	up
C15orf52	-0.523734157	6.832898068	-2.649183549	0.009164692	0.041385188	down
NOS2	-0.831735959	6.735801195	-2.645116803	0.009269247	0.04174177	down
KIAA1586	-0.571023725	5.696054339	-2.644705353	0.009279885	0.041756738	down
CTSW	-0.715984058	5.842107615	-2.644130298	0.009294771	0.041815484	down
TRMT2A	-0.575379874	7.4290922	-2.642938133	0.009325702	0.041905102	down
TPRG1	-0.667203712	6.780861058	-2.64194685	0.009351491	0.041987939	down
LOC100127974	0.592197469	4.829232294	2.640739867	0.00938298	0.042119326	up
DMP1	0.520129717	4.431991543	2.640524646	0.009388604	0.042129725	up
TPSAB1	0.810077345	9.464580525	2.638255255	0.009448101	0.042305261	up
SPTA1	0.610945088	4.518960342	2.638181635	0.009450037	0.042305634	up
ZNF831	0.522398904	4.553118058	2.633718542	0.009568061	0.042758561	up
LOC101928631	0.534693463	6.04319993	2.632677637	0.009595778	0.042865648	up
KRT5	-0.767570638	11.0783118	-2.632134706	0.009610264	0.04288841	down
LOC101928303	-0.569763988	5.522821258	-2.630698326	0.009648683	0.042984369	down
TSSK3	-0.648432989	5.021886722	-2.630627189	0.009650589	0.042984369	down
TNIP3	0.784219244	4.101251024	2.629075644	0.009692252	0.043144691	up
PHEX-AS1	0.667016812	4.880566105	2.628992008	0.009694502	0.043146298	up
SFTPC	1.393858823	7.601226006	2.628895144	0.009697109	0.04314949	up
PCNX3	-0.691794941	7.502269421	-2.624284986	0.009821918	0.043611379	down
LOC101928583	0.520087839	4.651981582	2.624070614	0.009827757	0.043628821	up
CLVS1	-0.533999475	3.938829839	-2.622851649	0.009861017	0.043750957	down
KIF21B	-0.564816503	4.487516311	-2.617768728	0.010000803	0.044225083	down
SRPX	0.608804515	5.429016789	2.617503184	0.010008154	0.044231897	up
TAAR2	-0.635946242	4.913878888	-2.617114186	0.010018933	0.044270965	down
GTSE1-AS1	-0.521893671	5.634895288	-2.615487046	0.010064131	0.044401949	down
PITX2	0.711357358	4.17875235	2.615296367	0.010069439	0.044416789	up
AVPR1A	-0.712180937	6.386213866	-2.614281582	0.010097734	0.044532997	down
MRGPRF	-0.59099723	4.914309207	-2.608459077	0.010261471	0.045011707	down
PMEL	-0.50248931	6.571215676	-2.607308897	0.010294097	0.045111494	down
KIF15	-0.59519171	5.570369684	-2.606907544	0.010305504	0.045144156	down
NWD2	0.687200659	3.892496831	2.605574868	0.010343462	0.045252823	up
ESRG	0.63185865	3.481710575	2.600443018	0.010490808	0.045754028	up
LOC105371967	0.707286189	4.53194353	2.596764334	0.010597592	0.04615804	up
CCL20	-0.961578836	7.03163431	-2.595168283	0.010644225	0.046319276	down
ANKRD36C	0.546641531	6.515846129	2.594588071	0.010661223	0.046364445	up
SP6	-0.551769413	5.731305194	-2.592262189	0.01072961	0.046582011	down
CT55	-0.535096602	4.258951202	-2.590476667	0.010782377	0.046740006	down
LOC101928700	0.664214653	4.469598777	2.589230634	0.010819338	0.046846872	up
LOC105372881	-0.614158087	5.689644515	-2.588540583	0.010839856	0.046917922	down
PHACTR3	0.66272817	5.596184314	2.587697879	0.010864961	0.046964272	up
LOC100505878	0.688931131	3.048053595	2.58712925	0.01088193	0.047002036	up
ADRB3	0.513818742	4.696327836	2.584676159	0.010955411	0.047256851	up
FCGR1CP	0.5985291	8.021460149	2.582080469	0.011033649	0.047504603	up
C8A	0.633130678	4.576732166	2.581120811	0.011062702	0.047602762	up
SH3RF3-AS1	0.504742872	3.404735092	2.579399692	0.011114979	0.047764709	up
SNORA5B	-0.644010641	7.238654571	-2.578798306	0.011133298	0.04783443	down
TDRD5	-0.580817155	4.203716352	-2.577366195	0.011177031	0.047968179	down
SNX31	-1.027543534	7.102775243	-2.575783846	0.011225532	0.048122065	down
GLIS3-AS1	-0.528281816	6.091497451	-2.572846119	0.011316078	0.048455645	down
TAF1L	-0.605468598	5.708493903	-2.572163579	0.011337209	0.0485017	down

FXYD7	-0.517248325	4.61938226	-2.571819833	0.011347864	0.048528059	down
PRR34	-0.654342471	4.951192229	-2.570717431	0.011382098	0.048628922	down
LOC101928475	0.730142129	3.902813327	2.56872261	0.01144428	0.048848893	up
LINC00639	0.679212054	4.431068769	2.568015118	0.011466407	0.048920735	up
LINC00474	0.521832531	3.918072961	2.567741696	0.011474969	0.048943292	up
OPN4	-0.502030827	4.588961981	-2.567415178	0.011485201	0.048977785	down
LINC00029	0.57699159	5.274638178	2.567051391	0.01149661	0.049015257	up
GNA14-AS1	0.533142023	5.85340282	2.566998149	0.011498281	0.049015257	up
LINC00327	0.649415653	4.250680212	2.565443472	0.01154716	0.049168554	up
LOC101927060	-0.631775457	5.088574892	-2.563085507	0.011621653	0.049402841	down
RMI2	-0.556374684	7.279368612	-2.5612549	0.011679782	0.049585332	down
HCAR1	-0.633114734	4.446173069	-2.560869044	0.011692068	0.049619041	down
HMHB1	0.62605373	5.044259512	2.560595161	0.011700795	0.049646854	up
LOC101927460	-0.575221729	4.627768697	-2.558717244	0.011760795	0.049864374	down
GDF10	0.723602332	4.107896511	2.557781084	0.011790809	0.049945262	up
